# Multiplexed deep visual proteomics resolves spatial heterogeneity and rare endocrine states in human pancreatic islets

**DOI:** 10.1038/s41467-026-77890-6

**Published:** 2026-09-25

**Authors:** Mariya Mardamshina, Nicolai Dorka, Marvin Thielert, Frida Björklund, Frederic Ballllosera Navarro, Anna Martinez Casals, Ferenc Kovacs, Christophe Avenel, Jocelyn E. Manning Fox, Peter Horvath, Carolina Wählby, Patrick E. MacDonald, Matthias Mann, Emma Lundberg

**Affiliations:** 1 Department of Bioengineering, Stanford University, Stanford, CA USA; 2 Proteomics and Signal Transduction, Max Planck Institute of Biochemistry, Martinsried, Germany; 3 Synthetic and Systems Biology Unit, Biological Research Centre, HUN-REN, Szeged, Hungary; 4 Single-Cell Technologies Ltd, Szeged, Hungary; 5 Department of Information Technology and Science for Life Laboratory, Uppsala University, Uppsala, Sweden; 6 Department of Pharmacology and Alberta Diabetes Institute, University of Alberta, Edmonton, AB Canada; 7 Institute for Molecular Medicine Finland (FIMM), University of Helsinki, Helsinki, Finland; 8 Institute of AI for Health, Helmholtz Zentrum München, Neuherberg, Germany; 9 Science for Life Laboratory, School of Engineering Sciences in Chemistry, Biotechnology and Health, KTH - Royal Institute of Technology, Stockholm, Sweden

**Keywords:** Cellular imaging, Proteomic analysis, Proteomics

## Abstract

Understanding tissue function requires connecting spatial cellular context with molecular depth, yet such integration remains challenging in complex organs like the pancreas. We develop multiplexed Deep Visual Proteomics (mxDVP), an end-to-end workflow combining high-plex imaging, automated computational analysis, and spatially guided ultra-sensitive mass spectrometry. Powered by PIPΣX, an open-source image analysis framework enabling whole-slide membrane-aware segmentation, annotation, and laser microdissection export, mxDVP achieves >6000 protein identifications from as few as 100 small islet cells. Applied to human pancreatic islets, mxDVP segments over 860,000 cells and resolves twelve endocrine subtypes, including rare polyhormonal and intermediate-state populations that exhibit spatial organization patterns, co-expression of INSM1 and SCG3, and hybrid α/β/δ signatures. These findings reveal a spectrum of endocrine heterogeneity and spatial organization. By integrating imaging and deep proteomics in an accessible framework, mxDVP enables discovery of rare cell states whose biological roles depend on tissue architecture.

## Abstract (web summary)

Linking spatial context to molecular depth in tissue remains difficult. Here, the authors combine high-plex imaging, open-source image analysis and ultra-sensitive mass spectrometry to profile single endocrine cell types in human pancreatic islets, including rare bi-hormonal and polyhormonal states.

## Introduction

Spatially resolved proteomics requires integrating two powerful but disconnected technologies: multiplexed tissue imaging for spatial context and mass spectrometry for molecular depth. While multiplexed imaging platforms can visualize dozens of markers across millions of cells, they provide limited molecular information. Conversely, mass spectrometry-based proteomics achieves remarkable depth (>8000 proteins per sample) but loses spatial relationships. Deep Visual Proteomics (DVP) has bridged this gap for selected tissue regions^1–4^, but extending spatially resolved proteomics to rare, individual cells within well-mixed complex tissues has remained technically prohibitive. The challenge lies not in the capabilities of imaging or proteomics individually, but in the lack of an integrated workflow that seamlessly connects spatial identification with precise molecular profiling.

Achieving this integration demands overcoming multiple technical barriers: (i) computational processing of whole-slide multiplexed images containing hundreds of thousands of densely packed cells, (ii) accurate segmentation and annotation in challenging tissue architectures, (iii) seamless coordinate transfer between imaging and laser microdissection systems and (iv) precise spatial targeting of rare cell populations identified through multiplexed markers. Current workflows fragment these steps across multiple software platforms, requiring manual data transfer, coordinate system reconciliation, and programming skills, creating bottlenecks that limit spatial proteomics to specialized laboratories.

For tissues like pancreatic islets, where rare polyhormonal cells, often excluded as artifacts in single-cell sequencing^5,6^, may play critical roles in disease, these technical limitations prevent investigation of biologically important populations. Accumulating evidence from orthogonal approaches supports the biological reality of such rare cell populations in healthy adult islets, including their detection at the protein level by single-cell western blot^7^ and their documentation across the human lifespan^8^.

Here we present multiplexed Deep Visual Proteomics (mxDVP), an integrated end-to-end workflow that connects high-plex tissue imaging with spatially guided, ultra-sensitive mass spectrometry for deep proteomic profiling. While recent work has demonstrated the principle of coupling multiplexed imaging with DVP for tumor microenvironment profiling^4^, no prior implementation has provided a fully open-source, end-to-end computational pipeline capable of whole-slide-scale analysis with automated LMD export, nor has the approach been applied to the systematic characterization of rare cell states in complex endocrine tissues. mxDVP combines three innovations: (i) optimized multiplexed imaging protocols compatible with downstream proteomics, (ii) PIPΣX, an open-source computational framework that unifies image preprocessing, whole-slide segmentation (>800,000+ cells), interactive annotation, and automated coordinate export for laser microdissection, all without requiring programming expertise, and (iii) refined laser microdissection and sample preparation enabling >6000 protein identifications from as few as 100 small islet cells. Unlike fragmented workflows requiring multiple software tools and manual coordination, mxDVP provides a unified, accessible pipeline where spatial identification directly guides molecular profiling.

We demonstrate mxDVP by comprehensively mapping human pancreatic islets, analyzing 864,485 cells through multiplexed imaging and profiling targeted populations at high proteome depth. This reveals twelve endocrine subtypes including organized polyhormonal populations with distinct spatial distributions and proteomic signatures, demonstrating how integrated spatial molecular analysis enables investigation of rare cell states inaccessible to conventional approaches. mxDVP establishes a generalizable platform for spatially resolved proteomics across diverse tissue types, with PIPΣX and all protocols openly available to facilitate community adoption.

## Results

**Fig. 1:**
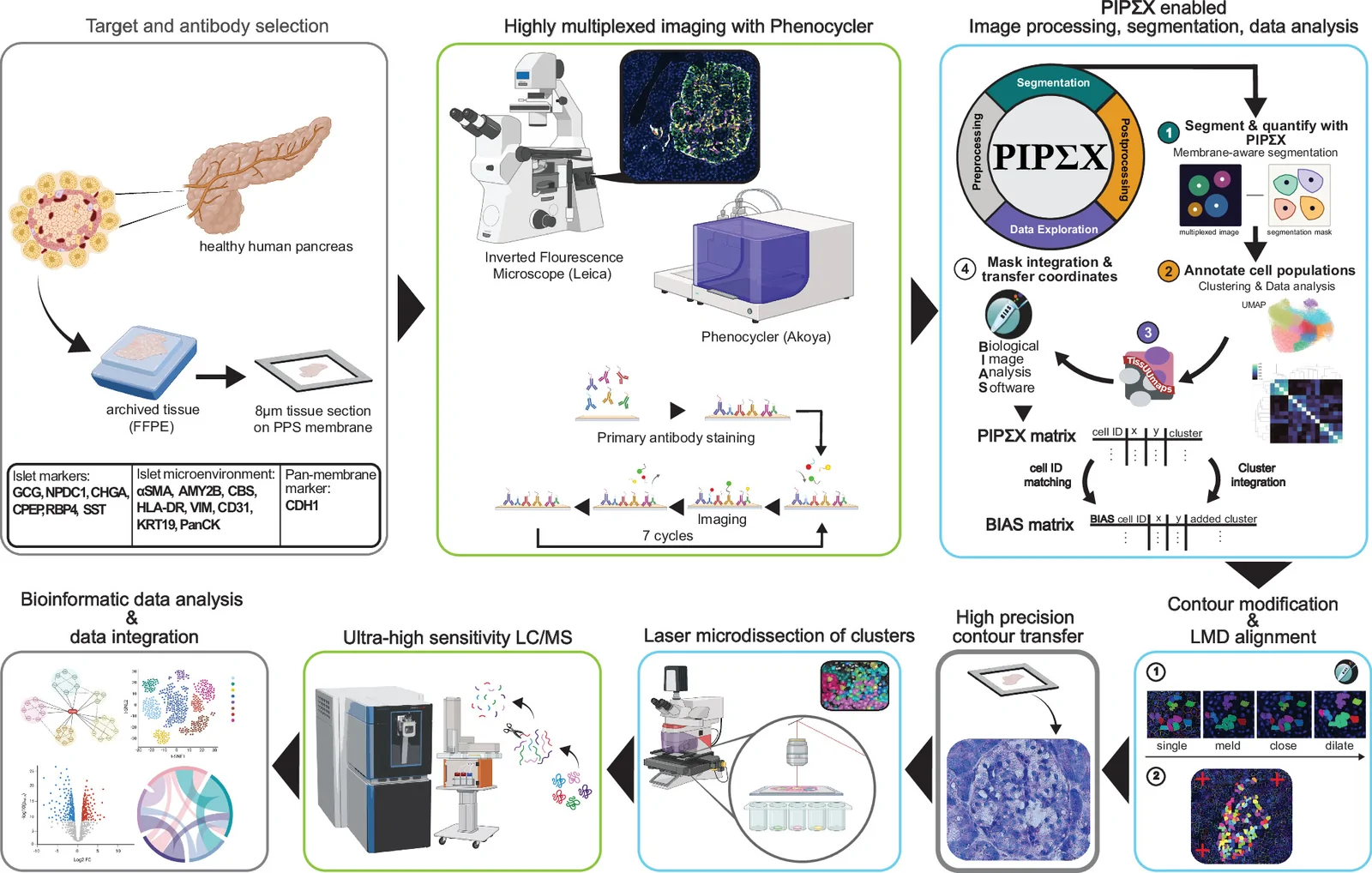
Workflow of the integrated mxDVP pipeline. Schematic of the integrated mxDVP pipeline; no statistical analysis. The pipeline consists of the following key steps (1) development of a customized antibody panel, (2) generation of high-plex spatial proteomic images, (3) image and data analysis through an open-source image analysis suite PIPΣX, (4) contour modification and alignment point selection, (5) introduction of polychromatic stain for high-precision contour transfer, (6) laser microdissection of selected clusters, (7) MS-based proteomic profiling of isolated cell populations, and (8) integrated data analysis. Gray borders represent steps introduced to complete the end-to-end pipeline. Green borders showcase fully automated experimental processes within the pipeline. Cyan borders represent steps in the pipeline that require significant customization. Figure created in BioRender. Mardamshina, M. (2026) https://BioRender.com/t1z7llc and Affinity Designer (Serif). Microscopy and tissue images within the figure were acquired by the authors from the tissue sections analyzed in this study. No generative AI was used to create any element.

We developed a robust and fully integrated pipeline for mxDVP combining automated multiplexed imaging, integrated image analysis by an open-source Python software suite called PIPΣX, laser microdissection (LMD), and ultra-sensitive mass spectrometry (MS) (Fig. 1). This pipeline is optimized for archival formalin-fixed paraffin-embedded (FFPE) tissues, enabling deep single-cell proteomic analysis while preserving spatial context. However, the mxDVP pipeline can also be used for any tissue preservation, tissue type, diseased/non-diseased state, and other spatial omic analysis. By leveraging the same tissue section for both imaging and mass spectrometry, we ensure high data fidelity.

### Open-source PIPΣX enables versatile image analysis and integration

A critical component of the mxDVP pipeline is PIPΣX, an open-source, end-to-end software suite that streamlines multiplexed tissue image analysis and exploration. Unlike existing workflows that require coding expertise or fragmented toolchains, PIPΣX offers a user-friendly graphical interface accessible via desktop installation or a web-portal version requiring no local setup, guiding users through each step with built-in explanations, recommended parameters, and quality control checkpoints (Fig. 1; Supplementary Figs. 1, 2).

PIPΣX improves segmentation accuracy in densely packed pancreatic islet cells by combining StarDist-based nuclear detection with membrane-aware watershed refinement (Supplementary Figs. 1a, 2b). To handle gigabyte-scale whole-slide images, PIPΣX incorporates automatic downscaling for segmentation followed by upscaling and reintegration, maintaining coordinate alignment across all downstream steps. A typical run completes in about 10 minutes for a tissue microarray core (~10,000 cells) and around one hour for a whole-tissue section of about 260,000 cells (Supplementary Fig. 1b). Adaptive preprocessing modules correct illumination gradients, tiling artifacts, and background noise, ensuring robust performance even for archival or low-quality tissue sections (Supplementary Figs. 1a, 2a). Multi-metric single-cell quantification, computing mean marker intensity, 90th percentile intensity of non-zero pixels, fraction of pixels above threshold, and a normalized Otsu separation score, combined with clustering and annotation refinement tools, enables the reliable identification of both abundant and rare cell populations. The 90th percentile metric is particularly effective for low-abundance, spatially restricted signals such as granule-localized hormones or transcription factors that may be missed by conventional mean-based quantification. (Supplementary Fig. 2d–f).

The pipeline integrates with TissUUmaps and QuPath, allowing visualization, quality control, and exploration of marker distributions (Supplementary Fig. 2b)^9,10^. External segmentation masks from Cellpose, DeepCell, or CellSAM can also be imported at user-defined pipeline stages, making PIPΣX model-agnostic and compatible with custom segmentation strategies without bypassing other analysis steps. Importantly, PIPΣX supports direct export of single-cell contours and seamless integration with Biology Image Analysis Software (BIAS), pyLMD, and Leica LMD software^11,12^, introducing a post-segmentation adjustment tool that enables high-precision laser microdissection of annotated populations for downstream proteomics (Supplementary Fig. 2c). This tight integration between open-source image analysis and proteomic sampling ensures reproducibility, minimizes tissue loss, and enables unbiased capture of rare or transitional states. Crucially, the pyLMD export includes automated assignment of calibration coordinates for LMD software compatibility, eliminating manual calibration steps and supporting multiplexed calibration within a single tissue section for parallel isolation of distinct cell populations.

Beyond its role in mxDVP, PIPΣX was developed as a standalone software package, designed to be broadly useful for spatial analysis of diverse tissue landscapes and cellular compositions, including complex pathological states such as cancer, necrosis, or fibrosis. Performance benchmarking across pancreas, kidney, mammary gland, tonsil, esophageal, and multiple cancer tissue types, spanning 2 to 40+ channel panels, confirmed robust preprocessing, accurate segmentation, and cell-wise quantification with minimal user intervention across all datasets (Supplementary Fig. 1b). Its semi-automated annotation strategies simplify and accelerate cell type identification without requiring custom-trained segmentation models for each tissue type. This flexibility increases throughput and significantly lowers the barrier for adopting high-dimensional spatial proteomics in diverse research settings.

Together, these innovations establish PIPΣX both as an enabling backbone of the mxDVP workflow and as a broadly applicable, open-source platform for multiplexed image analysis. While established tools such as QuPath with StarDist or Cellpose plugins provide robust segmentation capabilities, they do not integrate membrane-aware refinement constrained by morphological priors, multi-metric quantification including 90th percentile intensity and Otsu separation scoring, rule-based annotation refinement with confidence scoring, or automated LMD export with calibration coordinate assignment within a single no-code environment. PIPΣX addresses this gap while remaining interoperable with QuPath/TissUUmaps for visualization and with BIAS for contour processing, ensuring that users can adopt it alongside or in place of existing tools without workflow disruption.

### Creating an end-to-end automated multiplexed DVP workflow for high-precision proteomics

Pancreatic islets exhibit tightly packed cells, with an intricate spatial organization and phenotypic variability, making their profiling particularly challenging. To address this, we designed and validated a 15-plex DNA-barcoded antibody panel tailored for islet cells and their surrounding microenvironment (Fig. 1, Supplementary Fig. 3a). This panel enables visualization of hormone-secreting cells (CHGA, GCG, NPDC1, RBP4, CPEP, SST) alongside exocrine (CBS, AMY2B), immune (HLA-DR), ductal (PanCK, KRT19), and vascular components (VIM, CD31, αSMA), providing a holistic spatial view of pancreatic islet architecture (Fig. 1, Supplementary Fig. 3a).

For multiplexed imaging, we used the CODEX (Co-detection by indexing; Phenocycler System, Akoya Biosciences) platform^13,14^ that leverages DNA-barcoded antibody technology. To ensure the compatibility with mass spectrometry we compared the proteome content in CODEX-stained tissue and naive consecutive tissue sections (Supplementary Fig. 3b) and confirmed that structural and membrane proteins were preserved.

In this dense tissue context, high-quality laser microdissection is key which relies on precise contour transfer and isolation as well as optimal membrane slide selection. We found that PPS membranes outperformed PEN membranes in offering superior signal-to-noise ratios (Supplementary Fig. 4a, PEN: 5.7, PPS: 40.3) and lower autofluorescence. PPS membranes also demonstrated superior durability, and better preservation of tissue integrity throughout the multiplexed staining and cell dissection (Supplementary Fig. 4a). To improve the precision of the contour transfer and single-cell isolation, we implemented a polychromatic staining step using Toluidine Blue after the multiplexed imaging, similar to H&E staining but with better water resistance and long-term stability. This staining maintained tissue integrity and was compatible with downstream proteomic analysis, as evidenced by the consistent identification of >6000 protein groups from Toluidine Blue-stained tissue sections (Supplementary Fig. 4b–d). Lastly, key proteins regulating pancreatic cell function, such as transcription factors, are notoriously challenging to detect due to their low abundance. To overcome this, we optimized cell pooling strategies, progressively increasing the number of cells per sample. We found that the pooling of 250 two-dimensional cell contours (equivalent to the volume of 100 cells) per MS injection provided the optimal balance between rare cell population coverage and proteomic depth, significantly enhancing the number of unique proteins identified while keeping coefficient of variation values below 10% (Supplementary Fig. 4e–g).

### High-resolution spatial profiling unveils diverse endocrine subtypes in normal pancreatic islets

**Fig. 2:**
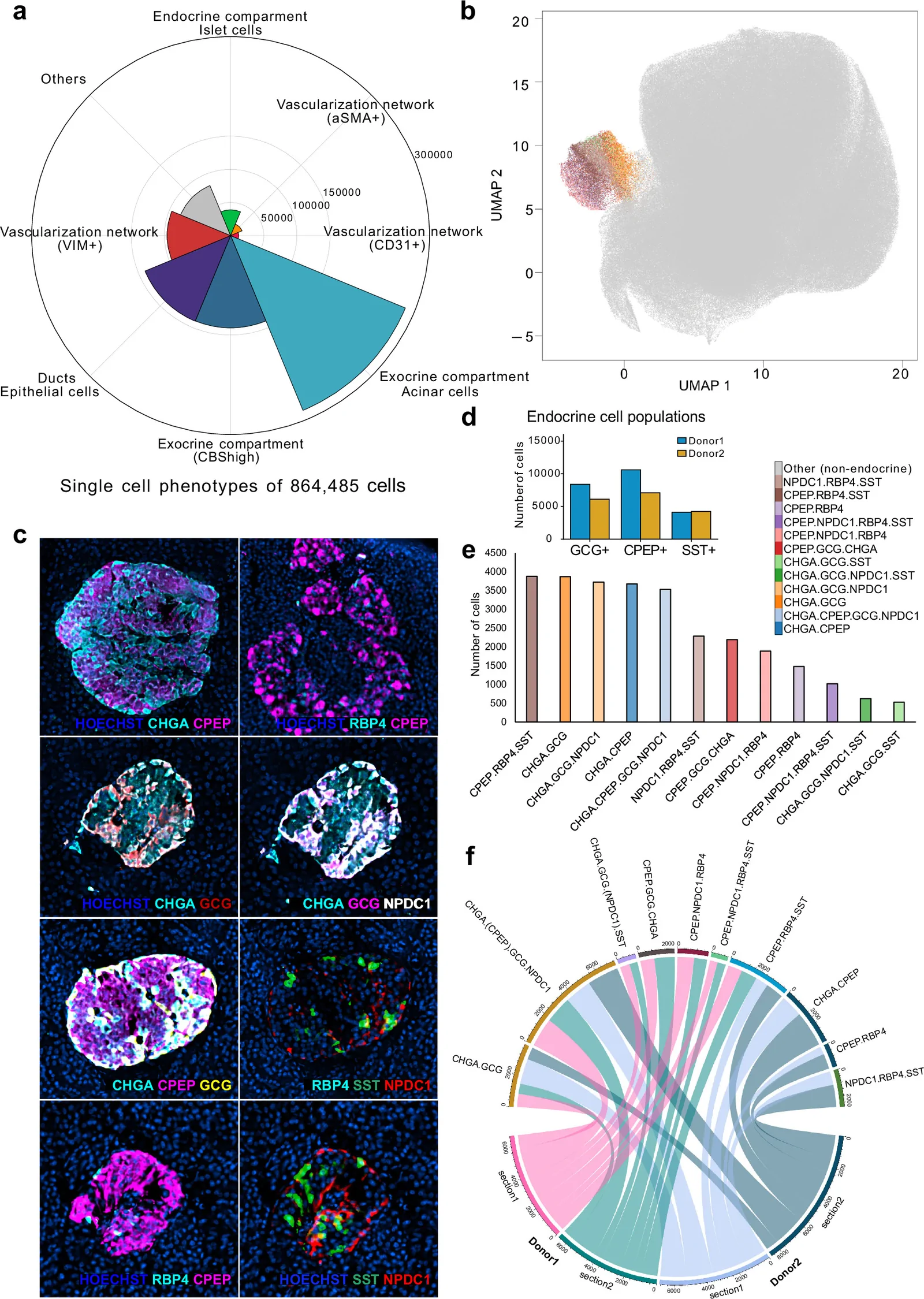
High-plex spatial proteomic profiling reveals distinct endocrine cell populations. **a** Rose diagram displaying the number of cells per annotated cell type for the full combined multiplex imaging dataset. **b** UMAP representation of the annotated high-plex imaging data of the combined dataset, where the endocrine cell population is highlighted and colored by identified subpopulations. **c** Representative multiplex images of pancreatic islets with markers distinguishing co-expression patterns identified by K-means clustering. **d** Quantification of endocrine cells expressing GCG, CPEP, and SST across donors. The bar plot shows the number of cells per donor. **e** Number of cells per identified endocrine cell populations, showcasing both rare and more abundant populations. **f** Circos plot comparing the identified endocrine subpopulations between donors and consecutive sections.

Using our mxDVP pipeline, we generated a detailed spatial profile of endocrine cell populations in normal pancreatic tissue. To ensure reproducibility, two biological replicates with two sections from two donors were included (See Supplementary Table 1). The multiplexed imaging data was processed using the PIPΣX pipeline, enabling precise segmentation and annotation of 864,485 individual cells (Fig. 2a). The dataset included 285,169 acinar cells expressing AMY2B and 139,204 acinar cells expressing CBS and AMY2B. Epithelial (ductal) regions constituted 139,662 cells, while vascular components were represented by 95,925 VIM^+^ cells, 18,726 αSMA^+^ cells, and 12,211 CD31^+^ cells. A further 53,216 cells were assigned to annotated categories outside these compartments (“Others”, Fig. 2a), while a separate nonislet subset of 81,620 cells (9.4%) remained unannotated due to limited marker availability. Among the annotated cells, 38,753 (5.0%) were identified as islet cells (Fig. 2b).

The pancreatic endocrine component includes insulin-producing beta cells (β), glucagon-secreting alpha (α) cells, somatostatin-secreting delta cells (δ), pancreatic polypeptide-producing gamma (PP) cells, and ghrelin-secreting epsilon cells (ε). To explore heterogeneity within the endocrine compartment, we applied K-means clustering to the islet cell subset. The clusters were annotated based on their expression profiles, with reference images carefully reviewed for segmentation artifacts and staining imperfections (Supplementary Fig. 5a–d). In total, we identified 14,491 GCG^+^ cells, 17,689 CPEP^+^ cells, and 8340 SST^+^ cells (Fig. 2c, d). These counts are not mutually exclusive, as cells co-expressing more than one hormone marker are included in each corresponding category, and therefore sum to more than the total number of annotated islet cells. Established coexpression patterns, such as CHGA and CPEP, CPEP and RBP4, and CHGA and GCG, were readily identified (Fig. 2c; Supplementary Fig. 6a). These patterns were consistently observed in mass spectrometry data, with marker abundance varying systematically between imaging-defined subtypes and showing modest spread among biological replicates of the same subtype (Supplementary Fig. 5e). The graded, rather than binary, marker abundance across imaging-defined clusters suggests that endocrine cell identities exist along continuous expression spectra rather than as discrete categories. Additionally, we observed a distinct CPEP⁺RBP4⁺SST⁺ co-expressing population in which the relative imaging-level intensities of CPEP, RBP4, and SST varied from cell to cell within the same annotated cluster (Supplementary Fig. 6a). Intriguingly, we also detected rare marker combinations, including SST and NPDC1, as well as triple-marker profiles such as GCG, SST, and NPDC1, underscoring the sensitivity of this approach in identifying rare cell states (Supplementary Fig. 6b). The subclustering revealed endocrine subtypes, characterized by diverse receptor coexpression patterns. The most abundant subtypes included CPEP⁺RBP4⁺SST⁺ (3875 cells), CHGA⁺GCG⁺ (3870 cells), and CHGA⁺GCG⁺NPDC1⁺ (3721 cells), along with CHGA⁺CPEP⁺ (3673 cells). Rare subtypes, such as CHGA⁺GCG⁺SST⁺ (622 cells) and CHGA⁺GCG⁺NPDC1⁺SST⁺ (527 cells), represented the least abundant populations in the cohort (Fig. 2e). Polyhormonal populations represented 1.11% of cells in donor R322 and 0.57% in donor R348, with CPEP⁺NPDC1⁺RBP4⁺SST⁺ showing the greatest inter-donor variability. The presence of polyhormonal populations was further confirmed in publicly available CODEX imaging data from three additional independent donors via the Human Pancreas Analysis Program, with consistent co-expression patterns observed across samples processed by independent laboratories (Supplementary Fig. 7a–c). Notably, classical bi-hormonal classification based solely on GCG, CPEP, and SST co-expression identified mixed-lineage cells in only one of the two donors (R322: 0.70%; R348: 0%), whereas inclusion of NPDC1 as an alpha cell lineage marker revealed additional bi-hormonal and polyhormonal populations in both donors (R322: 1.11%; R348: 0.57%), highlighting the value of expanded marker panels in capturing the full spectrum of endocrine cell heterogeneity.

A comparison between the two donors revealed that the more common subtypes, such as CHGA⁺GCG⁺ and CPEP⁺RBP4⁺SST⁺, were reproducibly detected in both. In contrast, rare subtypes, including those with NPDC1 coexpression, were predominantly donor-specific (Fig. 2f). These observations are consistent with patterns identified in publicly available single-cell transcriptomics datasets^15^ (Supplementary Fig. 6c). While α (GCG⁺GC⁺TTR⁺), β (INS⁺IAPP⁺), and δ (LEPR⁺PRG4⁺SST⁺) cells are traditionally defined by distinct marker expression^16^, accumulating evidence suggests that this rigid classification falls short in capturing the underlying biology. Studies have shown that metabolic stressors such as high-fat diet or GLP-1R signaling can induce β-like traits in α-cells, altering their electrophysiology and marker expression^17–19^. Similar plasticity has been observed in type 1 diabetes, where reduced ARX and DNMT1 levels in α-cells coincide with β-like transitions^20^. Our deep proteomic profiling and refined islet cell annotation, identifying both canonical subtypes and hybrid states, including CPEP⁺RBP4⁺SST⁺ and CPEP⁺NPDC1⁺RBP4⁺, highlight the dynamic and adaptive nature of endocrine cell identity.

### Spatial analysis links islet size to vascularization patterns and polyhormonal cell states

**Fig. 3:**
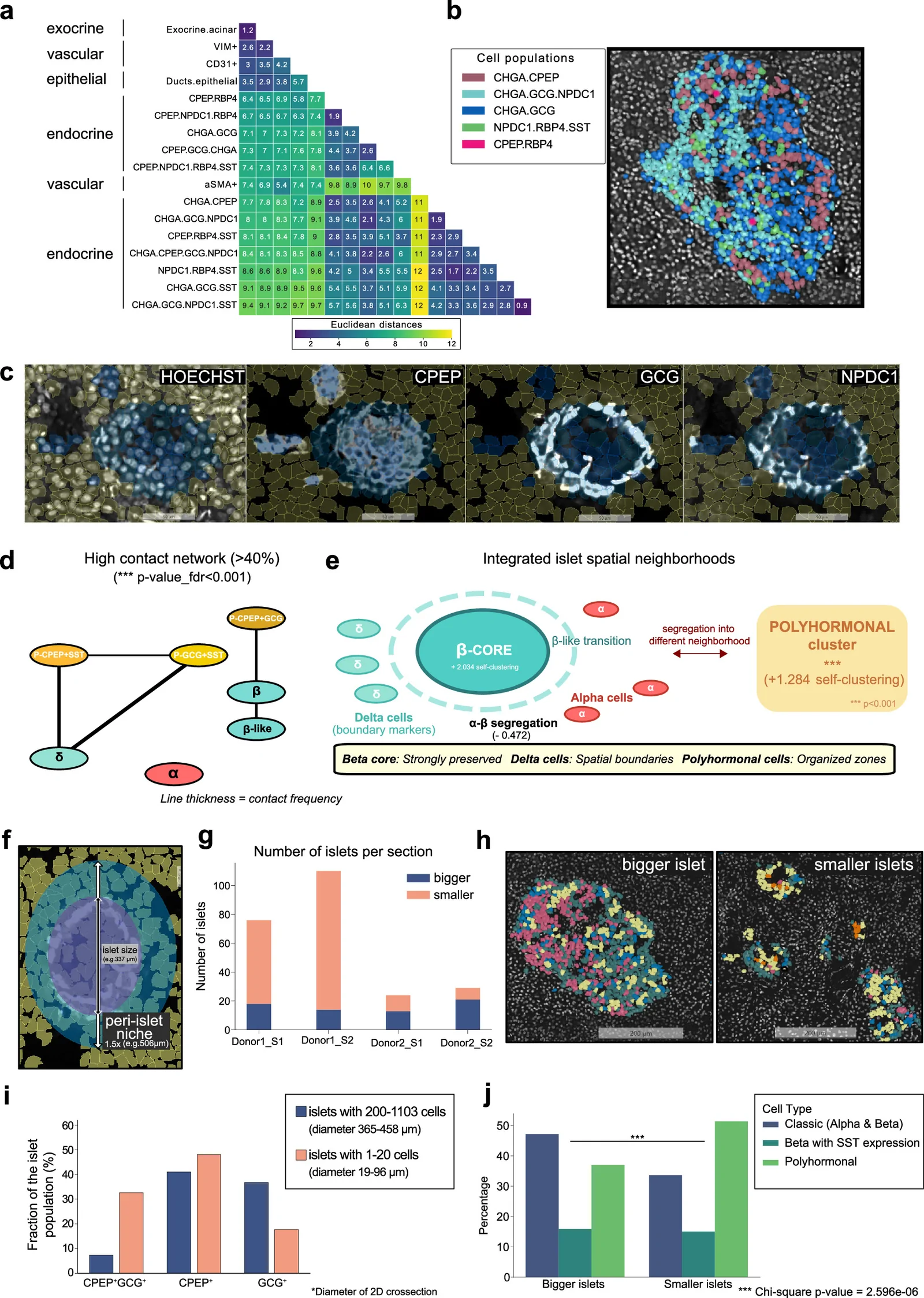
Islet-centered spatial analysis shows that islet size correlates with cell type composition of islets and the vascular bed. **a** Heatmap displaying the Euclidean distances between all annotated cell populations, quantifying the spatial relationships in the tissue microenvironment. **b** Example image of observed islet cell type compartmentalization. Cell segmentation masks are colored by the annotated cell type and overlaid on the nuclear stain image. **c** Illustrative example images of observed compartmentalization of β-cells (CPEP) and α-cells (GCG). Cell segmentation masks overlaid the images showing endocrine cells (blue) and other cell types (yellow). **d** High-contact network analysis reveals preferential spatial associations among endocrine subtypes. Network diagram shows cell populations with >40% contact frequency (two-sided binomial test against a 10% random-contact expectation, Benjamini–Hochberg FDR correction; *n* = 119 displayed edges, all FDR-adjusted *p* < 3.92 × 10⁻⁴). Exact adjusted p values for all edges are provided in the Source Data file. Node colors indicate cell identity: yellow (polyhormonal populations), blue (β-cells and β-like cells), pink (α-cells). Line thickness represents contact frequency between populations. Polyhormonal populations, like CPEP⁺GCG⁺SST⁺, show high mutual contact frequency. α-cells exhibit lower contact frequency with other endocrine populations. **e** Integrated spatial model of islet architecture reveals organized polyhormonal zones and preserved β-cell core structure. Schematic representation synthesizing neighborhood enrichment analysis across all analyzed islets. β-cells form a strongly self-clustering core (mean enrichment +2.034 across four tissue sections, range 1.03–3.41, all FDR-adjusted *p* < 0.001), with δ-cells positioned at compartment boundaries. α- and β-cells showed reciprocal spatial avoidance (mean enrichment −0.472 across both directions and four sections, *n* = 8 comparisons, 7 of 8 with FDR-adjusted *p* < 0.05; the remaining comparison, α → β in R348_S1, FDR-adjusted *p* = 0.34). Polyhormonal cells form spatially segregated zones with significant self-enrichment (mean +1.284 across four polyhormonal subtypes and four sections, *n* = 16, range 0.632.56, all FDR-adjusted *p* < 0.001). Two-sided permutation test with 1000 iterations, Benjamini–Hochberg FDR correction; the resolution limit of the permutation test is *p* < 0.001. Enrichment scores calculated as log₂((observed +1)/(expected +1)); positive values indicate spatial attraction, negative values indicate avoidance relative to random spatial distribution. **f** Representative image of the investigated peri-islet microenvironment. The central ellipse superimposed on the image (purple) highlights the investigated islet, and the surrounding ellipse (blue) demarcates the peri-islet microenvironment. **g** Quantification of the number of islets per sample, showcasing the difference in islet size between sections. **h** Example images of representative bigger and smaller islets segmentation masks colored by integrated AI-driven annotation of islet cell type populations. **i** Proportions of GCG-, CPEP-, and GCG/CPEP-expressing cell populations in small islets (fewer than 20 cells) and large islets (more than 200 cells). **j** Comparison of cellular composition in different-sized islets. Statistical significance was determined by the Chi-square test (*p*-value = 2.596 ⨯ 10^−6^; two-sided).

Leveraging the spatial resolution of our multiplexed image dataset, we performed Euclidean distance-based spatial analysis to quantify the proximity of the annotated cell types and their microenvironmental relationships (Fig. 3a). This analysis revealed distinct organizational patterns reflective of functional compartmentalization. Endocrine cell populations exhibited closer spatial distances among themselves compared to their proximity to exocrine or ductal cells, underscoring their cohesive niche organization into islets of Langerhans. Similarly, exocrine cell types displayed lower Euclidean distances within their compartment, highlighting their spatial clustering and potential cooperative roles in digestive processes. In contrast, vascular and ductal compartments were spatially segregated from other cell types, indicating distinct structural organization within the tissue (Supplementary Fig. 6d).

Within the vascular compartment, we observed a correlation between marker expression and vessel caliber. Specifically, αSMA⁺ cells were predominantly associated with larger-caliber vessels (>80 μm in diameter), whereas CD31⁺ endothelial cells were enriched in capillary-sized vessels (<10 μm in diameter) (Supplementary Fig. 6e). Spatial analysis further supported this distinction, as αSMA⁺ cells were positioned at greater distances from endocrine cell clusters, suggesting potential spatial segregation between these populations. Compartmentalization was also evident within pancreatic islets. Similar to the patterns reported by Bosco et al.^16^, α-cells, identified by GCG expression, were predominantly localized to the islet periphery, whereas β-cells, marked by CPEP expression, were concentrated toward the islet center (Fig. 3b, c; Supplementary Fig. 6f). Despite this spatial partitioning, α- and β-cells remained in close proximity, as indicated by low Euclidean distances, likely facilitating coordinated interactions crucial for metabolic regulation^16,21^. Rare cell populations exhibited two distinct spatial behaviors. NPDC1⁺, RBP4⁺, and SST⁺ cells were sparsely dispersed throughout the endocrine compartment but remained in close proximity to α- and β-cells (Euclidean distances 2.2–4.5; Fig. 3a), consistent with embedding within the classical endocrine niche. In contrast, the more complex polyhormonal populations (CHGA⁺GCG⁺SST⁺ and CHGA⁺GCG⁺NPDC1⁺SST⁺) showed consistently higher Euclidean distances to other endocrine cell types (range 8–12; Fig. 3a), forming spatially segregated zones detailed below. Beyond compartment-level organization, we examined finer spatial relationships among endocrine cell populations. Neighborhood enrichment analysis revealed that polyhormonal cells exhibit distinct spatial patterning (Fig. 3d, e, Supplementary Fig. 8a). These polyhormonal populations formed spatially segregated zones with significant self-enrichment (enrichment score +1.284, *p* < 0.001) and showed spatial avoidance of pure α-, β-, and δ-cells (mean neighbor enrichment −0.374, *p* < 0.001). Notably, despite the substantial presence of polyhormonal cells, β-cells maintained strong core clustering (enrichment score +2.034, *p* < 0.001), with δ-cells positioned at compartment boundaries between polyhormonal zones and the β-cell core. All *p*-values derived from permutation test (1000 permutations), Fisher’s combined method across sections, FDR-corrected (Benjamini–Hochberg). Those findings were also reflected in the contact frequency of the pancreatic cell populations (Supplementary Fig. 8b). This spatial architecture suggests organized microdomains within islets rather than random cellular intermixing. Building on these spatial observations, we next examined the relationship between islet size (i.e. two-dimensional cross-section) and cell type composition (Fig. 3f, Supplementary Fig. 8c, d), both across donors and within consecutive tissue sections from the same donor (Fig. 3g). For this comparison, islets were categorized as small (<20 cells) or large (>200 cells) (Fig. 3h). It is important to note that the size classification was based on two-dimensional tissue sections, and some small islets may represent cross-sections of the top of larger islets. First, we examined the relationship between islet size and cellular composition by assessing the distribution of GCG-, CPEP-, and GCG/CPEP-expressing cells. Consistent with the findings of Lehrstrand et al.^22^ and Murrall et al.^23^, we found that GCG-expressing cells were predominantly localized within larger islets, whereas smaller islets contained a higher proportion of CPEP-expressing cells (Fig. 3i). Notably, the highest percentage of GCG/CPEP co-expressing cells was observed in smaller islets. To further quantify this relationship, we analyzed islet size in relation to endocrine cell populations, revealing a significant association between the two variables (Chi-square test, Fig. 3j). Larger islets primarily consisted of classical α- and β-cells, with CHGA⁺ and CPEP⁺ cells predominantly localized within them. In contrast, smaller islets were enriched in polyhormonal populations, often displaying a relatively uniform representation of specific endocrine subtypes (Fig. 3i). This size-dependent enrichment of polyhormonal cells in smaller islets provides spatial context for the organized polyhormonal zones observed in our neighborhood analysis, suggesting that these architectural features are particularly prominent in smaller islet structures. This suggests that smaller islets may reflect localized environmental demands or dynamic shifts in endocrine cell mass. This structural heterogeneity within the pancreatic microenvironment prompted us to investigate the peri-islet niche. We expanded analysis regions to 1.5 times the diameter of each islet across our dataset (Fig. 3f) and examined the peri-islet microenvironment and its relationship to islet size. Large islets exhibited lower vascular representation, particularly of αSMA⁺ cells, and a higher proportion of acinar cells with elevated CBS expression. This pattern may indicate hypoxic states due to sparse vascularization, as previously suggested^24^. In contrast, small islets displayed increased vascular representation, including larger vessels containing αSMA⁺ and VIM⁺ cells. This enhanced vascularization could be critical for meeting the metabolic and oxygen demands of smaller, potentially newly forming or expanding islets, providing a structural framework for nutrient and oxygen delivery^25^.

### Deep proteomic characterization of endocrine cell populations

To uncover the molecular diversity of pancreatic endocrine cells, including rare polyhormonal states, we leveraged the mxDVP pipeline for deep proteomic profiling. Using laser microdissection, we precisely isolated subpopulations from the same multiplexed tissue sections, integrating AI-driven annotation and automated cell targeting. Each subpopulation was processed independently across two donors and tissue sections to ensure robust representation (Supplementary Table 4).

**Fig. 4:**
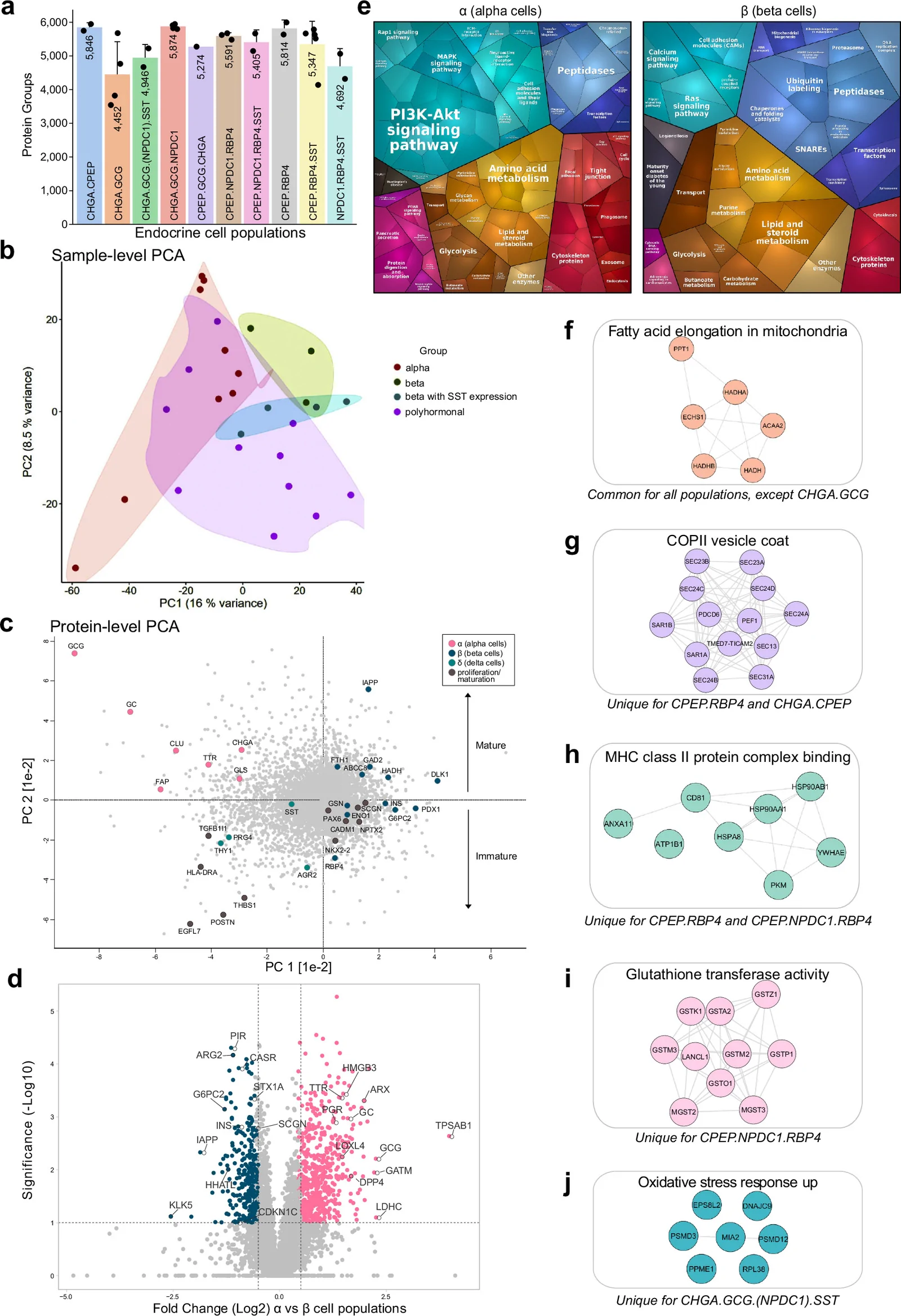
Integrated mxDVP pipeline allows for proteomic characterization of distinctive islet cell populations. **a** Number of protein groups identified by MS for each isolated endocrine cell population across both donors, with the mean number of protein groups indicated above each bar. Each dot represents one independent MS sample (*n* = 28 samples in total). The unit of study is a single MS sample, each derived from 250 individually excised, pooled contours (~100 cell equivalents) of a given annotated endocrine subtype from a given donor and tissue section. Samples are biological replicates in the sense that they derive from independent contour pools; both donors are represented. Error bars show standard deviation. No statistical comparison between populations is performed in this panel. **b** Sample-level principal component analysis of the entire cohort after outlier removal, colored by population (alpha, beta, beta with SST expression, polyhormonal). Shaded regions indicate the extent of each population. Variance explained by each component is indicated on the axes. **c** Protein-level principal component analysis showing protein loadings, with markers of α-cells (pink), β-cells (blue), δ-cells (turquoise), and proliferating/maturing cells (dark gray) highlighted. PC1 separates α- and β-cell markers; PC2 corresponds to cellular maturity, indicated by the mature/immature axis. **d** Volcano plot comparing α-cell (pink) and β-cell (blue) proteomic profiles. The x-axis shows log₂ fold change between α- and β-cell populations and the y-axis shows significance (−log₁₀). Welch *t*-test, two-sided, with Benjamini–Hochberg correction (FDR < 0.05). Dashed lines indicate the fold-change and significance thresholds. **e** Proteomaps^92^ pathway enrichment analysis illustrating functional processes enriched in α-cells (left) and β-cells (right). Each polygon represents a KEGG pathway, with size correlating with the ratio between the groups. This visualization highlights distinct functional processes between the two endocrine cell types. **f**–**j** Protein-protein interaction networks of the endocrine subpopulations, generated using the STRING database and colored by enriched pathway (Fisher exact test, Benjamini–Hochberg FDR *q* < 0.02). The populations in which each network is enriched are indicated beneath each panel.

Across both donors, we identified about 6000 unique proteins, revealing distinct and deep proteomic signatures among classical islet cell types (Fig. 4a; Supplementary Fig. 8e-g). Principal component analysis (PCA) revealed segregation of α- and β-cells along PC1, while the beta-delta population was separated from β-cells along PC2. Polyhormonal cells displayed the greatest heterogeneity, with a broad spread overlapping all other populations, consistent with their mixed hormonal identity (Fig. 4b, c). Protein loadings showed clear separation between α- and β-marker proteins along PC1, while PC2 correlated with cellular maturity markers (Fig. 4c). For instance, α-cells were enriched for GCG, GC, CLU, TTR, CHGA, GLS, and FAP, reinforcing their roles in glucagon secretion and metabolic regulation. Delta (δ) cells, marked by SST, PRG4, AGR2, and THY1, suggested a proliferative, maturing population^26^. β-cells exhibited biological heterogeneity at the subpopulation level, with mature β-cell pools expressing IAPP, HADH, DLK1, FTH1, and ABCC8, while immature β-cells were distinguished by PDX1, ENO1, G6PC2, and RBP4^27,28^. Notably, markers linked to β-cell differentiation, PAX6, NKX2-2, and CADM1, were enriched in immature populations, suggesting ongoing maturation or potential cycling between insulin-high and insulin-low states^29–31^. It should be noted that this heterogeneity reflects differences between imaging-defined subpopulations and not single-cell variation.

To explore functional differences across cell types, we performed one-dimensional enrichment analysis (Supplementary Fig. 9a). β-cells were enriched in metabolic processes, including hexose biosynthesis, carboxylic acid metabolism, and ER-to-Golgi transport. α-cells, in contrast, were associated with hormone activity, focal adhesion, and ER lumen function^32,33^. A comparative analysis (Welch *t*-test; FDR < 0.05) identified hundreds of subtype-specific proteins, with α-cell markers (GCG, TTR, GC, ARX, LOXL4) and β-cell markers (INS, IAPP, PIR, G6PC2, SCGN) showing distinct clustering (Fig. 4d). Pathway analysis further revealed PI3K-Akt signaling, amino acid metabolism, and PPAR signaling in α-cells^34^, while calcium signaling, Ras signaling, and lipid metabolism were enriched in β-cells^35,36^ (Fig. 4e, Supplementary Fig. 9b).

Distinct metabolic demands were observed among endocrine subtypes: β- and δ-cells relied on fatty acid oxidation for sustained ATP production, whereas α-cells prioritized glucose sensing and glucagon secretion (Fig. 4f). Within β-cells, CPEP-expressing cells, identified as insulin-high cells based on our mass spectrometry data, exhibited enhanced COPII vesicle transport, a pathway critical for efficient proinsulin processing and secretion (Fig. 4g). This suggests that CPEP-expressing cells may upregulate COPII transport under metabolic stress to sustain insulin output^37^. Beyond metabolic adaptation, pancreatic islet cells co-expressing CPEP and RBP4 exhibited enrichment in the MHC class II protein complex binding pathway, suggesting a potential link between metabolic regulation and immune modulation (Fig. 4h). While C-peptide itself is not traditionally known for direct involvement in immune presentation, its presence alongside RBP4 may indicate metabolic stress or inflammation-associated changes in antigen processing. RBP4 is essential for retinol transport, and elevated levels have been linked to insulin resistance, raising the possibility that RBP4-expressing islet cells contribute to local immune signaling^38^. Although their direct role in diabetes pathogenesis remains unclear, the enrichment of MHC class II components suggests a potential function in antigen presentation within the islet microenvironment, warranting further investigation.

Beyond classical islet cell types, we identified rare populations exhibiting unique metabolic and stress-adaptive signatures, including gluconeogenesis, oxidative stress responses, and glutathione transferase activity (Fig. 4i, j)^39^. Notably, elevated glutathione synthesis has been linked to stressed β-cell states^40^, and shifts in arginine/proline metabolism have been associated with cellular plasticity and stress adaptation, mechanisms that may help preserve islet function and prevent apoptosis^41^.

Together, these findings demonstrate how deep proteomic profiling resolves endocrine cell heterogeneity, capturing metabolic demands, signaling networks, and stress adaptation mechanisms that shape pancreatic islet function.

### Exploring the spatial proteome of rare and bi-hormonal endocrine cell populations

To assess whether polyhormonal populations represent genuine biological states rather than technical artifacts, we first examined publicly available CODEX imaging data from three additional donors via the Human Pancreas Analysis Program. Co-expression of markers from distinct endocrine lineages was consistently observed across donors processed by independent laboratories using independent staining protocols (Supplementary Fig. 7a–c). The reproducibility of these populations across entirely independent cohorts and workflows argues against a protocol-specific or laboratory-specific technical origin. Within our own dataset, several methodological features further argue against a stacked-cell contamination scenario as described by Wang et al.^42^, tissue sections were cut at 8 µm thickness with membrane-aware watershed segmentation constraining cell boundaries to individual nuclei, and fluorescence imaging before and after laser capture microdissection confirmed precise contour isolation with minimal adjacent-cell signal (Supplementary Fig. 4c; Supplementary Movie 1). The precision of single-cell excision is shown directly in Supplementary Movie 1, in which individual annotated contours are dissected at high magnification while neighboring cells remain intact.

To further exclude the possibility that polyhormonal proteomic signatures arise from contamination by adjacent alpha and beta cells, we generated synthetic polyhormonal profiles by computationally mixing alpha and beta proteomes at varying ratios (25:75, 50:50, 75:25) using 1000 bootstrap replicates. Real polyhormonal cells differed from all synthetic mixes in 1427 proteins (FDR < 0.05 across all ratios), including significant upregulation of INSM1, IGF2BP3, PCNA, and CADM1, markers of endocrine differentiation, progenitor-like state, proliferation, and cell adhesion, as well as secretory vesicle components paired with reduced mature beta-cell and alpha-cell markers (SLC18A2, CPLX2, CADPS) and stress-response proteins (WFS1, STAT1, MX1, PDK4), none of which can arise from linear combination of classical cell type profiles (Supplementary Fig. 9c, d).

**Fig. 5:**
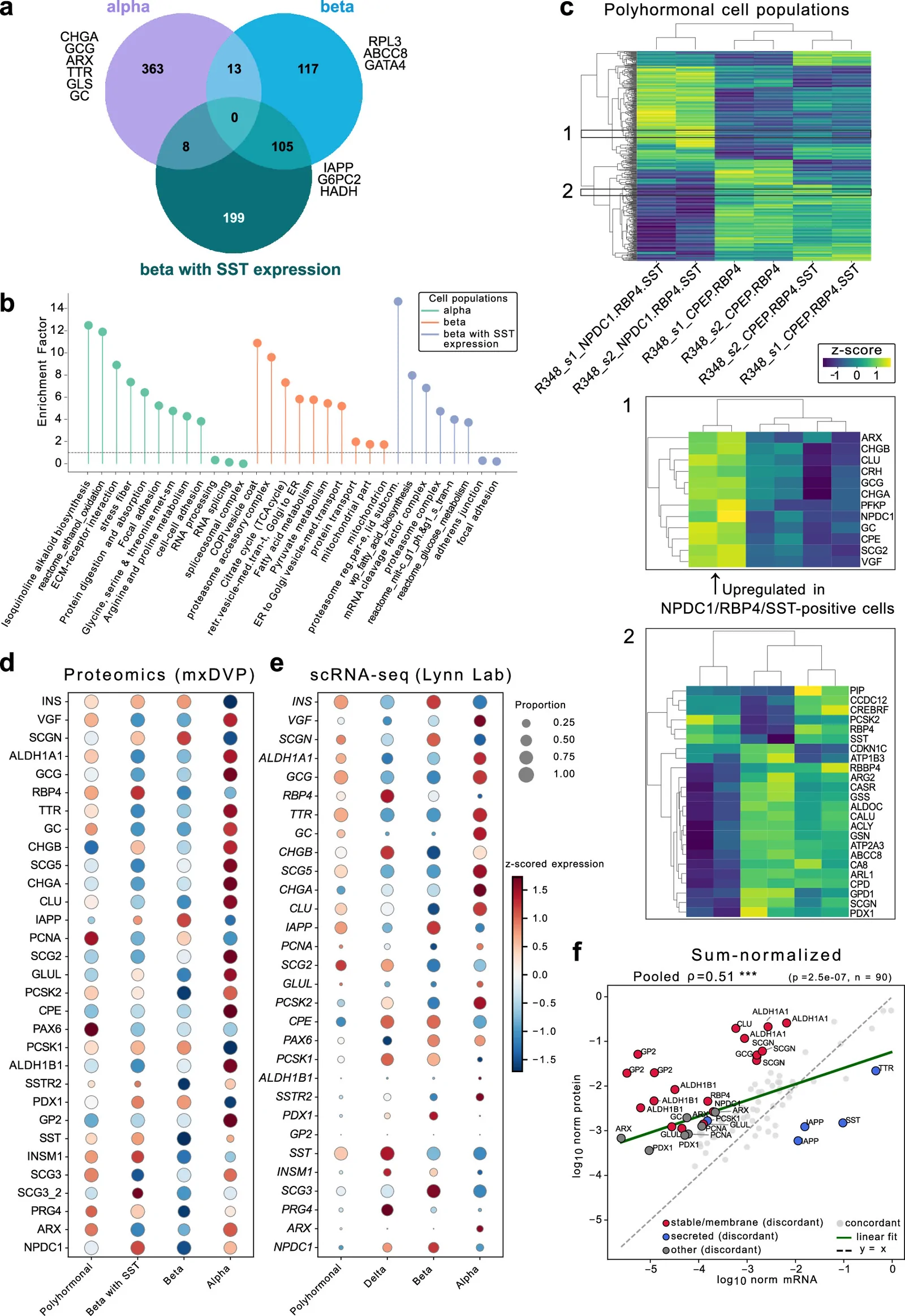
Deep proteomic profiling of bi-hormonal and polyhormonal cells characterizes rare dynamic endocrine cell states. **a** Venn diagram displaying proteomic differences between α-cells, β-cells, and bi-hormonal cells. Statistical significance of expression differences was determined by ANOVA test (*p*-value < 0.05) followed by PostHoc Tukey’s HSD test (two-sided; FDR < 0.05). **b** Enrichment analysis of cellular processes associated with enriched proteins, comparing cellular processes enriched in α-cells, β-cells, and bi-hormonal beta cells with SST expression. **c** Heatmap showing all differentially expressed proteins identified by mass spectrometry in selected RBP4-positive cell populations across the cohort (top). The first highlighted region (bottom left) illustrates representative α-cell-associated markers. The second highlighted region displays representative β-cell- and δ-cell-associated markers, along with several differentially expressed prohormones. **d** Dot-plot of mxDVP MS-based proteomic data visualizing the protein expression and proportion across polyhormonal, bi-hormonal, β-cells, and α-cells for key islet cell markers. **e** Dot-plot of single-cell transcriptomic data from a publicly available dataset^64^ showing the gene expression and proportion across polyhormonal, δ-cells, β-cells, and α-cells for key islet cell markers. **f** Correlation between protein and transcript abundance across the shared feature set (*n* = 30 genes), each normalized internally over the sum of the shared set and log₁₀-transformed, pooled across alpha, beta and polyhormonal cell types (Spearman ρ = 0.51, two-sided *p* = 2.5 × 10⁻⁷, *n* = 90). Points are colored by concordance and, where discordant, by protein class: secreted (blue), stable/membrane (red), other (gray); concordant features are shown in light gray. Discordance was defined as an absolute inter-modality difference >1 log₁₀ unit. The green line indicates the linear fit. The dashed line indicates the identity relationship and is shown for reference only, as the two modalities are normalized independently and are not on a common absolute scale. Source data are provided as a Source Data file.

Next, we focused on rare endocrine cell populations and started by investigating proteomic differences between classical subtypes and bi-hormonal cells (three subtypes). An ANOVA test (*p* < 0.05) followed by a PostHoc Tukey’s HSD test (FDR < 0.05) identified hundreds of upregulated proteins distinguishing these populations (Fig. 5a; Supplementary Data 1). Markers characteristic of the β-cells, including IAPP, G6PC2, SCGN, PICK1, and HADH, were detected in both classical β- and bi-hormonal (beta with SST expression) cells. Additionally, bi-hormonal cells exhibited co-expression of markers commonly shared between β- and δ-cells, such as CADM1, PDX1, and PCSK1^26^. PDX1, a master regulator of pancreatic development and β-cell function, plays a crucial role in both the formation and maintenance of insulin-producing β-cells, including their transdifferentiation from non-β-cells^43,44^.

Enrichment analysis revealed a strong metabolic signature in both classical β- and bi-hormonal subtypes, with pathways involved in the TCA cycle, fatty acid metabolism, and glucose metabolism (Fig. 5b). Notably, bi-hormonal cells also displayed enrichment for pathways associated with cell cycle regulation, mitotic G1 and G1-to-S transition, proteasome complexes, acetylation, and adhesion (Supplementary Fig. 10a). Cell adhesion, which plays a key role in insulin processing and exocytosis, has also been found to be enriched in bi-hormonal cell populations in zebrafish^45^. Furthermore, acetylation plays a critical role in pancreatic progenitor differentiation into mature β-cells by modulating key transcription factors such as PDX1 and MAFA, which govern insulin production and β-cell identity^46^. Notably, β-cells undergoing cell cycle entry often exhibit transient downregulation of key maturation markers, resulting in a less differentiated state that may temporarily reduce insulin production^47^. Given that bi-hormonal cells display both cell cycle activity and shared β- and δ-cell characteristics, their identity may reflect an intermediate or transdifferentiating state within the dynamic landscape of β-cell plasticity.

Expanding our analysis from bi-hormonal to the rarer polyhormonal cell populations (five subpopulations), we identified subtype-specific markers whose expression patterns aligned with multiplexed imaging data (Supplementary Fig. 10b). Among 23 overlapping proteins detected in NPDC1-positive polyhormonal cells, we observed a hybrid signature, with co-expression of α-cell markers (GCG, CHGA, PGR, MAOB) and β-cell markers (MAOA, ABCB10, VIL1) (Supplementary Fig. 10c). Interestingly, these polyhormonal cells also expressed DPP4, typically described as an α-cell marker in humans, consistent with our data (Fig. 4f), but reported to be expressed in β-cells in rats^48^. Notably, these cells also expressed ALDH1A1 and ALDH1B1, markers previously associated with endocrine progenitor states and retinoic acid signaling during islet differentiation^49,50^. Additionally, the prohormone VGF, which regulates insulin granule formation and β-cell function, was enriched in this population, supporting the idea that polyhormonal cells may contribute to islet cell replenishment and adaptation^51^.

Comparison of different RBP4-positive polyhormonal cells revealed hundreds of differentially expressed proteins (Fig. 5c). Notably, NPDC1/RBP4/SST-positive cells exhibited upregulation of α-cell markers (GCG, NPDC1, GC), alongside prohormones (SCG2, VGF, CPE) and ARX, a transcription factor that directs pancreatic endocrine progenitors toward the α/PP developmental pathway. Loss of ARX has been shown to trigger the reprogramming of adult α-cells into β-cells, underscoring a potential role for these polyhormonal populations in islet plasticity and adaptive endocrine remodeling^52^. Importantly, all markers identified through multiplexed staining were detected in our proteomic analysis. Additionally, we identified the prohormone VGF, ALDH1A1, as well as PCSK2, a marker of immature endocrine cells in CPEP/RBP4/SST-positive cells^53^. In contrast, CPEP/RBP4-positive cells exhibited upregulation of CDKN1C, a cell cycle inhibitor known to be expressed in mature β-cells, where it plays a critical role in limiting proliferation.

### Proteomic insights into hybrid polyhormonal endocrine states in pancreatic islets

Next, we examined the expression patterns of key islet cell markers, progenitor-associated factors, and proliferation markers across different cell populations (Fig. 5d). As expected, α-cells predominantly expressed GCG, ALDH1B1, and GLUL, whereas β-cells showed high levels of IAPP, INS, and SCGN. Polyhormonal cells exhibited a hybrid expression pattern, co-expressing islet markers while also displaying elevated proliferation markers and SCG3 expression. Beta with SST expression cells mirrored β-cell marker expression but showed higher SST levels. Notably, SCG3 isoform 2 was more highly expressed in beta with SST expression cells than in other cell types, suggesting potential functional differences. Interestingly, both beta with SST expression and polyhormonal cells demonstrated increased expression of distinct SCG3 isoforms. Stridsberg et al.^54^ previously reported Secretogranin co-expression in adult human islet cells, but its functional significance remains unclear. More recent studies have implicated SCG3 in carbohydrate metabolism, diabetes pathogenesis, and its role as a pro-angiogenic factor in diabetic retinopathy^55^. Additionally, SCG3 expression has been linked to NKX2-2 regulation and is potentially involved in β-cell mass loss through mature β-cell dedifferentiation and diabetes progression^56^. Another notable finding was the elevated expression of INSM1 in polyhormonal and beta with SST expression cells. INSM1 is a transcription factor known to play a crucial role in islet cell differentiation, β-cell maturation, and transdifferentiation from acinar to β-cells^57,58^. However, the precise mechanisms underlying its regulation and potential interactions with other non-conventional transcription factors in islet cell maturation and disease progression remain poorly understood.

To assess whether the polyhormonal proteomic signatures we identified are reflected in transcriptomic data, we compared our mxDVP measurements against the single-cell RNA-seq dataset from Yoon et al.^59^, which provides cell type annotations including a polyhormonal population. We arranged a shared marker set in identical order in both panels (Fig. 5d, protein; Fig. 5e, transcript) to enable side-by-side comparison across the annotated endocrine cell types (alpha, beta, delta, polyhormonal).

Canonical hormones and their associated processing enzymes (INS, GCG, SST, IAPP, CPE, CHGA, CHGB, SCG2, SCG5) showed consistent enrichment patterns at both the protein and transcript level across the corresponding endocrine cell types^59^. SCG3 and SCG3 isoform 2 showed significantly stronger enrichment in polyhormonal cells at the protein level than in the matched transcriptomic subtypes (Kruskal–Wallis *p* = 0.0012, comparing protein-level enrichment across cell types). INSM1, a transcription factor associated with endocrine differentiation, was elevated at the protein level in polyhormonal and beta with SST cells but did not show the same pattern in the corresponding scRNA-seq subtypes (Kruskal–Wallis *p* = 0.0233). Conversely, NPDC1 and ARX showed stronger differential expression patterns at the transcript level than at the protein level. To quantify this concordance, we correlated log-transformed expression of the shared gene set (*n* = 30 genes) across matched endocrine cell types, with each gene normalized internally over the sum of the shared set (Fig. 5f; Supplementary Data 5). Because not all populations had a reliable one-to-one cross-platform correspondence, we restricted the correlation to α, β, and polyhormonal cells; beta with SST proteomic cells were grouped with β cells, and delta and PP cells were excluded. Protein and transcript abundance were positively correlated in every matched cell type (Spearman ρ = 0.61, 0.45 and 0.46 for α, β and polyhormonal cells, respectively; pooled ρ = 0.51, *p* = 2.5 × 10⁻⁷), indicating broad concordance of relative expression between the two orthogonal datasets (Supplementary Fig. 10d). The canonical SCG3 isoform (Q8WXD2) was used for the correlation to match the single transcriptomic SCG3 measurement; both isoforms are shown separately in the dot plot (Fig. 5d).

These protein/transcript discordances are consistent with post-transcriptional regulation of secretory granule contents (SCG3, SCG2, VGF), which depend on regulated secretion rather than steady-state transcript abundance, and with the long protein half-life of endocrine transcription factors (INSM1, PDX1, PAX6) relative to their transcripts^60^. Crucially, the polyhormonal protein enrichment of SCG3, INSM1, and ALDH1A1 was not accompanied by a corresponding transcript enrichment in the same cell type: these genes were readily detected in the transcriptomic data but were not preferentially expressed in the polyhormonal transcriptomic population. This protein-level identity is therefore not predictable from steady-state transcript abundance, consistent with post-transcriptional regulation.

We note that the cross-platform comparison reflects concordance of relative expression patterns between independent datasets rather than a sample-matched comparison, and that dissociated single-cell RNA-seq is not by itself a definitive test of hormone co-expression; the transcriptomic data are therefore used here to compare relative feature abundance between modalities rather than as independent validation of these populations.

These findings underscore the power of our mxDVP pipeline in resolving complex cellular landscapes and identifying rare cell states within highly heterogeneous human tissues such as pancreatic islets. By integrating high-resolution multiplexed imaging with Deep Visual Proteomics, mxDVP enables in-depth characterization of rare endocrine subpopulations, including polyhormonal and intermediate-state cells, while providing protein-level insights that complement and extend transcriptomic analyses. The identification of distinct prohormone expression patterns, transcriptional regulators, and proliferation-associated factors in polyhormonal cells suggests a potential role in islet cell plasticity and adaptation. Notably, proteins such as SCG3 and INSM1, which are implicated in endocrine differentiation and β-cell function, exhibited differential expression in polyhormonal and beta with SST expression hybrid cells, highlighting avenues for further investigation into islet cell fate dynamics. Together, mxDVP showed promise to detect and characterize rare cell populations in a complex spatial context, providing a powerful and scalable framework to study cellular diversity across different organ systems in both health and disease.

## Discussion

Our study introduces multiplexed Deep Visual Proteomics (mxDVP) as an integrated, accessible approach for investigating pancreatic islet cell heterogeneity with high spatial and molecular resolution. By integrating multiplexed imaging with ultra-sensitive mass spectrometry, mxDVP provides insights into the complex cellular architecture of pancreatic islets as well as deep proteomic profiles to understand cellular activity, revealing rare endocrine states and spatial relationships that were not previously resolved. Wang et al.^42^ detected polyhormonal cells at ~4% frequency in IMC data but filtered them from analysis, noting that IMC’s Z-resolution of 4–8 µm prevented distinction between true co-expressing cells and stacked endocrine cells. Our approach differs from IMC in ways that constrain, but do not fully eliminate, this ambiguity. Membrane-aware single-cell segmentation restricts signal to individual cell footprints, and laser capture microdissection carefully isolates single annotated cells rather than ablating the full tissue section. These technical differences, alongside the greater protein sensitivity of mass spectrometry relative to IMC, provide both the spatial precision and molecular depth needed to characterize these rare populations. These findings are consistent with a growing body of evidence supporting bi-hormonal cells as genuine biological populations in adult human islets, including their documentation across the lifespan by immunofluorescence^8^, protein-level detection by single-cell western blot confirmed by confocal imaging^7^, and independent identification of a polyhormonal transcriptional trajectory across 18 scRNA-seq datasets comprising over 266,000 cells^61^.

A limitation of any section-based imaging approach, including ours, is that cells stacked along the axial dimension can contribute to an overlapping signal within a single optical section. Our tissue sections were cut at 8 µm, which is thinner than the mean diameter of a human endocrine cell (~9–13 µm)^62,63^. A section thinner than a cell body precludes the capture of two fully overlapping cells within a single plane, but it does not exclude partial axial overlap between the upper and lower portions of vertically adjacent cells, and this residual possibility cannot be ruled out on the basis of section thickness alone. Membrane-aware segmentation constrained to individual nuclei, and fluorescence imaging of the tissue before and after laser capture microdissection, reduce the contribution of adjacent cells, and the synthetic mixing analysis (Supplementary Fig. 9c, d) shows that the polyhormonal proteomes are not reconstructable as linear combinations of alpha- and beta-cell proteomes. Together these observations argue against simple two-cell stacking as the sole origin of the signal, but they do not exclude a contribution of stacked cells to the polyhormonal feature we describe. Definitive demonstration that individual cells co-express multiple hormones will require sensitive in situ single-molecule transcript imaging, ideally performed on dissociated cells, where the axial ambiguity inherent to tissue sections is removed. We regard the populations described here as protein-level co-expression states that are consistent with, but not proof of, genuine polyhormonal cells.

The identification of rare polyhormonal cells in normal pancreatic tissue underscores the functional plasticity of endocrine cells, challenging traditional views of islet composition. These rare cell populations, exhibiting co-expression of markers from multiple endocrine lineages, may represent transient states or early stages of transdifferentiation, possibly as an adaptive response to metabolic demands^64^. Although the current donors show low BMI and relatively healthy insulin secretion, these findings could indicate latent plasticity that may become more pronounced under conditions of metabolic stress, such as in older individuals, those with obesity, or those in a pre-diabetic state. Indeed, a broader comparison with these populations would be needed to test the hypothesis that these rare cell types are involved in the islet response to stress. Such findings would align with previous studies linking cellular plasticity to β-cell dedifferentiation and highlight potential mechanisms for maintaining islet function under stress^65^. Furthermore, the discovery of distinct endocrine subtypes suggests that cellular heterogeneity within islets extends beyond classical α-, β-, and δ-cell categorizations, revealing a spectrum of cellular identities with unique metabolic and regulatory profiles^66,67^. The proteins that define these populations, in particular SCG3 and INSM1, have not been validated in our own donor tissues by an orthogonal in situ method. Direct smFISH or immunofluorescence for SCG3 and INSM1 in the same tissue, combined with in situ single-molecule transcript imaging on dissociated cells, would establish both the cellular specificity of these markers and the existence of individual cells co-expressing multiple hormones. We regard this as the necessary next step for these populations.

We note that 9.4% of segmented cells (81,620 cells) remained unannotated, reflecting the constraints of our 15-marker panel, which did not include markers for all expected pancreatic populations such as PP cells (requiring PPY), epsilon cells (requiring ghrelin), or resident immune and stromal subsets beyond those captured by HLA-DR, VIM, and αSMA. These unannotated cells were distributed across both islet and exocrine compartments, suggesting they represent a mixture of missing endocrine and non-endocrine populations rather than a single overlooked cell type. While some of these populations may include rare or transitional states of biological interest, their characterization requires expanded marker panels. The modular design of PIPΣX and the scalability of CODEX to panels exceeding 50 markers make these populations accessible targets for future studies.

Spatial analysis further elucidates the organizational principles underlying islet biology. We observed that larger islets, predominantly composed of classical endocrine subtypes, exhibit reduced vascularization compared to smaller, polyhormonal cell-enriched islets. This differential vascularization may support distinct metabolic states or microenvironmental adaptations, such as hypoxic stress in larger islets. The correlation between islet size and cellular composition suggests that islet architecture may be dynamically regulated, influenced by local nutrient availability and metabolic demands^68,69^. Notably, the size-dependent enrichment of polyhormonal populations in smaller islets is consistent with the 3D whole-pancreas characterization by Lehrstrand et al.^22^, who demonstrated that islet composition and architecture are strongly size-dependent. mxDVP extends these structural observations by adding proteomic resolution within each size-stratified population.

Proteomic profiling of endocrine subtypes revealed unique pathway enrichments, with β-cells showing enhanced metabolic pathways and α-cells enriched for hormone secretion-related pathways^28^. The presence of transcription factors associated with progenitor states in polyhormonal cells suggests these populations may contribute to islet regeneration or plasticity^70^. Moreover, the differential expression of prohormones and immune-related proteins within these cells highlights their potential roles in local immune modulation and metabolic adaptation, providing new avenues for exploring islet cell resilience in diabetes and other metabolic disorders.

Importantly, we demonstrate that even when constrained to a limited panel of gold-standard markers typically used in routine immunofluorescence, our findings align with known endocrine cell distributions and heterogeneity trends reported in prior studies. However, the use of expanded panels incorporating additional endocrine subtype-specific markers allows more refined stratification of transient and rare endocrine states. When this refined spatial stratification is coupled with deep proteomic profiling, it enhances the resolution with which we can characterize intermediate or plastic states, offering mechanistic insights that are otherwise inaccessible through conventional marker sets alone.

Beyond pancreatic islets, the adaptable mxDVP pipeline provides a powerful framework for studying tissue heterogeneity across diverse physiological and pathological contexts. Recent applications of the standard Deep Visual Proteomics workflow have led to key translational biomedical discoveries, including the identification of novel targets in Hodgkin lymphoma, such as IRF4, proteasome subunits, and IL-signaling pathways^3^, as well as targetable JAK signaling in toxic epidermal necrolysis^1^. These studies underscore the potential of DVP to uncover disease mechanisms, inform targeted interventions, and advance precision medicine.

Concurrent with this work, Zheng et al. introduced mipDVP, integrating MACSima-based multiplexed imaging with DVP for tumor-immune profiling in colorectal and tonsil cancer^4^. While both approaches share the principle of coupling cycling-based high-plex imaging with spatially guided mass spectrometry, mxDVP addresses distinct methodological priorities. mipDVP depends on the proprietary BIAS software for its core image analysis and LMD export workflow. In contrast, PIPΣX provides a fully open-source, no-code alternative that can operate as a complete standalone pipeline, from preprocessing through segmentation, annotation, and LMD export, while also supporting seamless integration with BIAS, QuPath, and other established platforms when users prefer to retain familiar workflows. This dual-path design ensures that laboratories can adopt spatial DVP without proprietary software dependencies, while those already invested in existing tools can incorporate PIPΣX modules selectively. Additionally, mxDVP demonstrates whole-slide scalability exceeding 860,000 cells, quantification designed for low-abundance spatially restricted signals, and optimized tissue sample preparation achieving >6000 protein identifications from 100 cells, extending proteome depth beyond prior multiplexed DVP implementations. Similarly, openDVP^71^ has recently proposed a community-driven framework integrating MCMICRO, QuPath, and Napari with a QuPath-to-LMD web application. While openDVP emphasizes modularity across multiple established tools, PIPΣX consolidates these functions within a single environment with guided parameters and quality control checkpoints, reducing the coordination overhead inherent in multi-tool workflows. Together, these concurrent efforts reflect the growing recognition that accessible, integrated computational infrastructure is essential for bringing spatially resolved proteomics beyond specialist laboratories.

In this context, the integration of high-plex platforms like CODEX (Phenocycler System) is particularly valuable, as its DNA-barcoded antibody technology supports panels of over 50 markers in a single tissue section. This flexibility allows rapid adaptation of panels to distinct tissues and pathological states, including cancer, necrosis, and fibrosis, while its one-pot antibody staining, compatibility with FFPE tissue samples, automated microfluidics, and gentle tissue-clearing steps preserve tissue morphology and signal fidelity. Together with the open-source PIPΣX software, this high-plex imaging capability ensures that the mxDVP workflow remains versatile, reproducible, and widely applicable. This methodology facilitates the direct correlation of spatial organization with molecular signatures, enhancing our understanding of how cellular microenvironments influence function and pathology.

By offering an integrated end-to-end solution for high-resolution spatial proteomics, mxDVP bridges the gap between imaging and deep molecular profiling, enabling targeted in-depth characterization of rare cell populations that would otherwise remain undetected. These findings lay the groundwork for future investigations into islet cell fate dynamics and provide a blueprint for extending spatial proteomic approaches to other complex tissues. As spatially resolved proteomics continues to evolve, its application in understanding tissue microenvironments, disease progression, and therapeutic response is poised to drive advances in regenerative medicine and precision therapeutics.

## Methods

### Human sample procurement

Formalin-fixed paraffin-embedded (FFPE) tissue samples from the pancreas tail were obtained from two female donors by the Alberta Diabetes Institute (ADI) IsletCore (https://www.bcell.org)^72^. All donors’ families gave informed consent for the use of pancreatic tissue in research (University of Alberta Human Research Ethics Board approval Pro00013094). The research was approved by Etikprövningsmyndigheten (Dnr 2020-01690). The donors 1 and 2 (R322 and R348) were of the same sex, belonged to the same 40–49 age decade, and had no previous history of either diabetes mellitus or pancreatitis. Donor characteristics are described in Supplementary Table 1; all this data and full phenotyping information for this tissue can also be found at www.humanislets.com.

### Development of custom spatial proteomic pancreatic antibody assay

To allow visualization of key cell types and hormonal states in pancreatic tissue using multiplexed imaging, we developed and validated a panel of DNA-barcoded antibodies.

### Antibody validation by immunostaining

The targets included in the antibody panel were selected based on a combination of literature and patch-seq data^73,74^. Antibodies toward targets not supplied by Akoya Biosciences were sourced from commercial vendors as detailed in Supplementary Data 2. Clones were selected based on formulation (carrier-free), concentration (>0.5 mg/mL) per conjugation kit compatibility recommendations from Akoya Biosciences (CODEX User Manual Rev C ®), and validation data supplied by vendors, ensuring that the antibodies had been validated for immunohistochemistry (IHC) in FFPE tissue.

To ensure the specificity of the antibodies to be included in the assay, each antibody was validated by indirect immunofluorescence staining, using the protocol described by Martinez Casals et al.^75^. In brief, single sections of FFPE human, non-diabetic, adult pancreatic tissue were sectioned at 5 µm thickness and mounted onto positively charged microscope slides (VWR, Epredia, Cat. No. 76406-502). The samples were deparaffinized, rehydrated, and underwent heat-induced epitope retrieval (HIER) treatment using pH 6 citrate buffer (Sigma, Cat. No. C9999-1000mL) diluted to 1X in ddH2O in a pressure cooker (Bio SB TintoRetriever, Cat. No. BSB-7087) set to high pressure at 114–121 °C for 20 min. Following the HIER treatment, the samples were photobleached to reduce tissue autofluorescence. After four PBS washes, antibodies (all antibodies labeled as “Custom” in Supplementary Data 2) were diluted in antibody diluent containing 0.3% Triton (Sigma Aldrich, Cat. No. T8787) 1X PBS and subsequently added onto the samples and incubated at 4 °C overnight. The next day, the samples were washed three times with TBS-T (1X TBS (Medicago, Cat. No. 09-7500-100) 0.1% Tween (Sigma, Cat. No. P1379) and blocked using TNB (0.5% TSA blocking reagent (Akoya Biosciences, Cat. No. FP1012) in 1X TBS (Medicago, Cat. No. 09-7500-100) for 30 min. Next, secondary antibodies and nuclear stain diluted in TNB (1:800 secondary antibody as detailed in Supplementary Data 2 and 1:400 Hoechst (ThermoFisher, Cat. No. H3570), were added to the sections and incubated for 90 min at room temperature. Following three washes with TBS-T, the samples were mounted with Fluoromount-G (ThermoFisher, Cat. No. 00-4958-02) prior to image acquisition.

### Custom antibody conjugation

Pre-conjugated antibodies supplied by Akoya Biosciences were purchased with their respective barcodes as detailed in Supplementary Data 2. Antibodies that had been validated were custom-conjugated to their respective barcodes as described in Supplementary Data 2 following the conjugation protocol in Akoya Biosciences’ CODEX User Manual Rev C ® using the Conjugation Kit (Akoya Biosciences, Cat. No. 7000009). In brief, 50 µg of antibody was added onto a 50 kDa MWCO filter (EMD Millipore, Cat. No. UFC505096) and thereafter concentrated by centrifugation. Next, the antibodies underwent a reduction step through incubation with a reduction master mix at room temperature for 30 min. Following the reduction step, the buffer of the antibodies was exchanged for the Conjugation Solution (Akoya Biosciences, Cat. No. 7000009) by centrifugation. Next, lyophilized barcodes were resuspended in the Conjugation Solution, added to each respective antibody, and incubated for 2 h at room temperature. Following three washes by buffer exchange to the Purification Solution (Akoya Biosciences, Cat. No. 7000009), 100 µL of Antibody Storage Solution (Akoya Biosciences, Cat. No. 7000009) was added to the filter, and the purified, conjugated antibody was eluded and stored at 4 °C.

### Antibody conjugate validation by tissue staining

To validate whether the conjugation reaction was successful, the custom-conjugated antibodies were stained individually in FFPE human, non-diabetic, adult pancreatic tissue and visualized by manual addition of the complementary fluorescently labeled oligonucleotide sequence, also referred to as a reporter, as detailed in the CODEX User Manual Rev C ® by Akoya Biosciences. Largely the same protocol for tissue staining was used as described by Martinez Casals et al., with a few modifications to the end of the protocol to allow for the manual addition of reporters^76^. In brief, the same protocol for dewaxing, rehydration, antigen retrieval, and photobleaching was used as described above for immunostaining of tissues. Once the photobleaching was complete, the samples were washed twice in Hydration Buffer (Akoya Biosciences, Cat. No. 7000017) before being equilibrated in Staining Buffer (Akoya Biosciences, Cat. No. 7000017) for 30 min. Next, custom-conjugated antibodies diluted in a solution of Staining Buffer N, J, G, and S blockers (Akoya Biosciences, Cat. No. 7000017) were added to the samples and incubated at 4 °C overnight. The next day, the samples were washed twice in Staining Buffer before being fixed with 1.6% paraformaldehyde (ThermoFisher, Cat. No. 043368) diluted in Storage Buffer (Akoya Biosciences, Cat. No. 7000017) through a 10-min incubation at room temperature. Following three washes with 1X PBS, the samples were fixed in ice-cold methanol (Sigma-Aldrich, Cat. No. 322415) for 5 min. After three washes with 1X PBS, the samples were fixed with Fixative Solution (Akoya Biosciences, Cat No. 7000017) diluted 1:50 in 1X PBS through a 20-min incubation at room temperature before being washed three times with 1X PBS. Once the post-fixation was complete, the samples were washed three times with Screening Buffer (20% dimethylsulfoxide (DMSO) (Sigma-Aldrich, Cat. No. 472301) in 1X CODEX Buffer (Akoya Biosciences, Cat. No. 7000001). Next, reporters diluted 1:40 in Reporter Stock Solution (5% Assay Reagent (Akoya Biosciences, Cat. No. 7000002) and 0.25% Hoechst (ThermoFisher, Cat. No. H3570) in Screening Buffer) were added onto the samples and incubated for 5 min at room temperature before the samples were washed three times with Screening Buffer. Lastly, the samples were washed once with 1X CODEX Buffer (1:10 dilution of 10X CODEX Buffer (Akoya Biosciences, Cat. No. 7000001) in ddH2O) before they were mounted with Fluoromount-G before image acquisition.

### Image acquisition

Images were acquired using a Leica DMi8 widefield microscope equipped with a digital CMOS camera (ORCA-Flash 4.0 V3, Hamamatsu) and SOLA-SM-II light source at 20× magnification (20×/0.75 NA dry objective with a resolution of 0.325 µm/pixel) at 16-bits through the Leica Application Suite X (LAS X, v. 3.7.5.24914). Acquired images were merged and analyzed in ImageJ (v. 1.53) to determine the signal-to-noise ratio (SNR). A cutoff of 3 was used for the SNR for both primary antibody and antibody conjugate validations. The staining of each antibody was evaluated by comparing it to reference images provided by the manufacturer and the Tissue Atlas section of the Human Protein Atlas (HPA) to validate the specificity. The developed antibody panel has previously been published as an Organ Mapping Antibody Panel (OMAP) as part of the Human BioMolecular Atlas Program (HuBMAP)^77^. A representative dataset of CODEX images in human pancreas defining the main cell types and anatomical structures of human pancreatic tissue is accessible through OMAPs^78^.

### Highly multiplexed staining and imaging on membrane slides

#### Multiplexed immunofluorescence staining (CODEX)

For the highly multiplexed (CODEX) staining of the samples included in the cohort, single sections of FFPE human, non-diabetic, adult pancreatic tissue were sectioned at 8 µm thickness and mounted onto 22 mm × 22 mm × 4 µm custom-designed metal-framed PPS membrane coverslips (Microdissect). To dewax the samples, the membrane coverslips were placed onto glass slides with 100 µL ddH2O underneath and into a sealed packet of aluminum foil before being baked on a hot plate at 55 °C for 30 min to promote tissue adherence to the membrane and prevent excess dehydration. Once dewaxing was complete, the same protocol for CODEX tissue staining as described by Martinez Casals et al. was followed, with the exception that no photobleaching protocol was performed^76^. After the three post-fixation steps were completed, the samples were placed in Storage Buffer (Akoya Biosciences, Cat. No. 7000017) and stored at 4 °C.

#### Membrane slide testing

To select the LMD membrane, human spleen sections mounted on PEN and PPS membranes were stained with Hoechst and imaged on the Leica DMi8. For each membrane, signal-to-noise ratio was quantified in Fiji/ImageJ as (mean signal − mean background)/standard deviation of the background, with signal measured over five nuclear regions and background over five tissue-free membrane regions. Values represent one representative field per membrane.

#### CODEX image acquisition

Reporters were prepared according to the instructions of the CODEX User Manual Rev C ® by Akoya Biosciences and added to 96-well plates (Akoya Biosciences, Cat. No. 7000006). Prior to image acquisition, samples were equilibrated to room temperature in 1X CODEX Buffer (Akoya Biosciences, Cat. No. 7000001). The experimental template was prepared in CODEX Instrument Manager (CIM, v. 1.30.0.12), a software that automates the fluidic cycles and image acquisition, where the order of addition of reporters and exposure times were specified as detailed in Supplementary Table 2. Images were acquired with the same Leica DMi8 widefield microscope as described above at 20× magnification and 16-bit depth. To allow for visualization of the samples and CODEX image acquisition set-up, the samples were incubated with Hoechst (ThermoFisher, Cat. No. H3570) diluted 1:1000 in 1X CODEX Buffer for 3 min at room temperature before being washed once with 1X CODEX Buffer. A region of interest (ROI) was defined manually for each sample (ranging between 77 and 160 tiles, each tile 2048 × 2048 pixels and with a 10% overlap between tiles), and the focus was set automatically by LAS X for all manually placed focus points evenly distributed across the ROI. Each tile in the ROI was imaged as a 14.99 µm Z-stack, where each Z-step was 1.50 µm resulting in a total of 11 images in the Z-axis.

Images were processed and stitched in the CODEX Processor version 1.7.0.6 using background subtraction, deconvolution (vectorial, 25 iterations) and shading correction. The extended field feature was not enabled during processing.

#### Tissue storage and polychromatic staining pre-laser microdissection

Post-imaging tissue preservation conditions were optimized to maintain structural integrity. Once the CODEX imaging was completed, each sample was immediately removed from the microscope and washed twice for 1 min in ultrapure water (ThermoFisher, Cat. No. 51140) before being dehydrated through a series of 1-min incubations in increasing concentrations of ethanol (SigmaAldrich, Cat. No. 1009832500) (30%, 50%, 70%, 80%, and 90%) and a final 2-min incubation in 100% ethanol. Lastly, the samples were stored at 4 °C in 100% ethanol to preserve the tissues. Prior to laser capture microdissection samples were hydrated for one minute at 100%, 95% and 70% Ethanol followed by ultrapure water for 2 min. Finally, the sample was stained with Toluidine blue (Paradise Plus, ThermoFisher, Cat. No. KIT0312S) for 45 s and washed for 10 s with 75% ethanol before being loaded on the LMD7 system. A pseudocoverslipping step, involving the addition of 10 µL of 70% ethanol to the tissue section before imaging, enabled high-quality reference image acquisition for precise contour import. The tissue sections were dried for 2 min before dissecting contours. Microdissected samples were collected directly into a protein low-bind 384-well plate and stored dry at 4 °C after cutting.

#### PIPΣX: an integrated image analysis pipeline for all image processing steps

We centralized the image analysis pipeline by implementing an in-house developed software, PIPΣX, which allows performing all image processing steps i.e. pre-processing, segmentation, post-processing, and analysis in one place. PIPΣX (Pipeline for Image Processing and Exploration) is a modular, GUI-driven software framework developed in Python for the automated analysis of multiplexed tissue images (https://github.com/CellProfiling/pipex). It supports the complete image analysis workflow from raw image input to downstream data exploration, including preprocessing, segmentation, quantification, clustering, and visualization. The pipeline operates within a controlled software environment and is deployable across local and institutional computing systems. Image preparation steps, including the conversion of.qptiff files to individual.tif images and the extraction of single tissue crops from TMAs, were performed using utility scripts available at https://github.com/CellProfiling/ell_code_template/tree/master/examples.

### Image preprocessing

To address acquisition-related artifacts commonly present in whole-slide, multiplexed immunofluorescence images, PIPΣX includes a dedicated preprocessing module. This module provides a suite of configurable operations designed to improve image quality by correcting background noise, light gradients, and tiling inconsistencies. All preprocessing steps can be applied independently or in combination, and intermediate results are saved in JPEG format for visual inspection.

### Intensity thresholding

Minimum and maximum intensity thresholds (-threshold_min, -threshold_max) are used to remove background fluorescence and high-intensity artifacts such as tissue folds or precipitates. These thresholds are defined as percentiles of the global intensity distribution and allow for rapid suppression of outliers while preserving signal within the informative range.

### Otsu-based binning and filtering

Users can optionally define multi-level Otsu thresholding (-otsu_threshold_levels) to classify intensity values into discrete segments. Thresholding can be applied either with a default setting (e.g., 3, corresponding to three intensity classes with default bin filtering of 1:2) or with explicit bin specifications (e.g., 5:1:2), which can be used to generate masks or remove specific intensity bands from the original image.

### Tile correction and homogenization

To mitigate illumination inconsistencies and visible tile boundaries in large mosaic images, the module includes several corrective steps:

Gradient Light Correction (-light_gradient), where each tile is subdivided into smaller kernel patches, and local normalization is applied to correct for directional illumination artifacts and vignetting. Gradient complexity can be tuned with a value from 1 to 4, where higher values allow for finer-grained correction.

Brightness Homogenization (-balance_tiles), where tile-to-tile intensity variation is reduced through histogram-based scaling and standard deviation comparisons, harmonizing the brightness distribution across the entire image.

Stitch Smoothing (-stitch_size), where the interface regions between adjacent tiles are smoothed over a user-defined pixel width to eliminate visible seams and improve continuity.

### Exposure rescaling

The final preprocessed image can be globally brightened or dimmed using the -exposure parameter, which rescales pixel intensities relative to the original image. This is offered as a convenient (and optional) quality of life final step to avoid the need for an intensity rescaling in other visualizing software.

### Example configurations

The module has been used to successfully process diverse sample types with a range of acquisition challenges: Light gradients and uneven tile intensities were corrected using -threshold_min=1, -otsu_threshold_levels=5:1:2, -light_gradient=2, and -exposure=200.

In cases with strong background and pronounced tiling artifacts, the same parameters were sufficient to suppress noise and enhance contrast.

For more challenging gradients and poorly balanced tiles, a stronger correction (-light_gradient=3) and histogram normalization (-balance_tiles=yes) were applied.

Autofluorescent tissues were handled using more selective intensity binning (-otsu_threshold_levels=5:2:4) and moderate exposure boosting (-exposure=100).

Together, these preprocessing options enable users to flexibly salvage and improve low-quality images, supporting accurate segmentation and robust downstream analysis.

### Cell segmentation

Cell segmentation in PIPΣX is initiated using StarDist, a deep-learning algorithm for nuclei detection based on DAPI/Hoechst signals. The default pipeline includes nuclear segmentation using StarDist. Optional cell expansion from nuclei masks to approximate whole-cell morphology and membrane-aware refinement, activated when membrane marker channels are provided, using a watershed-based approach constrained by morphological priors. To manage large, whole-slide images, the pipeline includes automatic downscaling and reintegration steps for memory efficiency. Final segmentation outputs are saved as labeled NumPy arrays, and JPEG previews of each stage are generated for visual quality control.

External segmentation masks (e.g., from Cellpose, DeepCell, or CellSAM) can be imported into the pipeline at user-defined stages^79–81^. This ensures compatibility with custom models and varied segmentation strategies.

### Signal quantification and feature extraction

Quantitative feature extraction is performed for each segmented cell using the following steps: marker image normalization and multi-level Otsu thresholding, per-cell metric computation, including mean marker intensity, 90th percentile intensity of non-zero pixels, fraction of pixels above threshold, and normalized Otsu separation score. These features are compiled into a structured CSV table with spatial coordinates. Optional marker-based filtration excludes outlier cells (e.g., top 1% of DAPI/CDH1/CTNNB1 intensities) to reduce segmentation artifacts.

### Unsupervised clustering and annotation refinement

The pipeline supports unsupervised clustering (e.g., K-means, Leiden) and integrates a rule-based refinement module for biological annotation. Clusters are matched to user-defined marker expression rules and assigned hierarchical labels (cell group, type, subtype) based on a confidence score. Parameters such as rank_filter and min_confidence are user-adjustable to control annotation stringency.

### Export and interactive visualization

PIPΣX supports the automatic generation of multiple output formats: FCS files with per-cell data for flow-style visualization or external analysis tools, and GeoJSON overlays for QuPath visualization. The.h5ad files for TissUUmaps integration, including segmentation masks, marker intensities, cluster annotations, and spatial coordinates. HTML exports for browser-based interactive exploration using custom plugins (e.g., Spot Inspector, Feature Space, InteractionV&QC). All results are organized into a standardized output directory structure for reproducibility and ease of sharing.

### Batch processing

For multi-sample workflows, PIPΣX supports batch-mode processing. Each sample can be configured with its own preprocessing, segmentation, and analysis settings, enabling parallel processing of large, heterogeneous datasets (e.g., multiple CODEX panels or tissues, TMAs).

### PIPΣX segmentation and cell annotation used in pancreatic tissue samples for the whole mxDVP pipeline

TIFF images were processed locally in PIPΣX (version 1.0) for automated automated segmentation, filtration and clustering. Segmentation, filtration, and clustering analyses were performed using the following parameters: Nuclear segmentation was conducted with Hoechst staining, applying a nucleus diameter of 20 pixels (equal to 6.5 µm) and a definition threshold of 0.5 via the integrated StarDist algorithm. Membrane refinement was performed following nuclear segmentation, leveraging CDH1 intensity. The refinement parameters were set to a membrane diameter of 25 pixels (equal to 8.125 µm) and a compactness value of 0.9. The membrane refinement confidence score, calculated as the proportion of cell contours ultimately defined by CDH1 membrane staining during watershed expansion, reached 96%. PIPΣX generated quantification output as a CSV table, reporting marker intensities as the mean pixel intensity per region of interest (each cell contour). These steps were executed using a locally installed beta version of PIPΣX. To remove artifacts, cells were filtered based on size, excluding the bottom 1% and top 5% of the size distribution. TIFF images with the segmentation masks and analysis outputs were uploaded to the PIPΣX web platform (version 1.0) for interactive exploration, cell annotation and segmentation assessment.

### Semi-automated clustering analysis

First, K-means clustering was applied to non-normalized data, with K = 25, for each tissue section separately. A subsequent cluster refinement algorithm was implemented, incorporating ranking scores for each marker. The algorithm evaluates each cluster by comparing its ranked markers to predefined rules (annotations), assigning a confidence score based on alignment. In the final refinement step, the cluster annotation with the highest confidence is selected. Only markers with expression levels above zero were considered for refinement.

### Clustering of endocrine cell populations

To annotate endocrine cell populations, output tables from each tissue section were merged and normalized using log1p transformation and quantile normalization within PIPΣX (Supplementary Data 3). Endocrine cells were subsetted and further stratified by tissue section before applying K-means clustering to the normalized data. Various K-values were tested, with K = 7 yielding the most robust results. Because clustering was performed separately for each tissue section, the K = 7 clusters per section resolved to 12 distinct endocrine subpopulations once annotated clusters were matched across sections and pooled. Clusters were annotated based on Spearman rank correlation scores, calculated using only endocrine cell marker intensities. This approach identified 12 distinct endocrine cell subpopulations with diverse receptor coexpression patterns. Supplementary Data 3 reports all 864,485 segmented cells with their marker intensities, cluster assignments, and endocrine subtype annotations, and includes an additional column flagging the cells that were subsequently exported to BIAS and isolated by laser microdissection for proteomic analysis. Cells were selected for LMD based on their endocrine subtype annotation, prioritizing balanced representation of each of the 12 identified subpopulations across both donors and tissue sections, with all annotated cells of the target subtypes within accessible tissue regions exported for contour optimization.

To validate segmentation accuracy and cell annotation, the annotated clusters were exported to the PIPΣX-integrated TissUUmaps software for visualization, facilitating dynamic exploration of annotations, cellular neighborhoods, spatially reduced data, and segmentation masks^82,83^. Segmentation masks and cluster assignments were reviewed against the original images to ensure fidelity.

### Spatial analysis

The integrated dataset of annotated cell populations was used to analyze spatial associations. Mean intensities for each cell population were calculated, followed by Euclidean distance analysis to assess spatial relationships across all donors and annotated cell types.

To further investigate the spatial organization, we used TissUUmaps to visualize the spatial neighborhoods and quantify islet sizes with the Ellipse Drawing plugin. Islet diameters were extracted from the detailed report generated by TissUUmaps. To examine the immediate microenvironment, we expanded the islet perimeter by a factor of 1.5 and performed repeated measurements within the adjusted boundary. To assess associations between islet size and cellular composition, we performed a Chi-square test using the scipy.stats library in Jupyter Notebook (Python v3.9.21).

### Direct laser microdissection export (pyLMD integration)

PIPΣX includes an embedded module based on the open-source pyLMD library for laser microdissection (LMD) targeting. This feature allows users to export selected cell contours as LMD-compatible XML files for downstream proteomics or transcriptomics. The export process includes the identification of calibration points, shape dilation for precise contour cutting, transformation and optimization of laser paths, and XML file generation with merged and labeled targets. This integration supports multiplexed calibration and iterative refinement of cut regions within a single section.

### Segmentation mask creation and processing for BIAS integration

Cell segmentation masks were generated by PIPΣX as TIFF files. For integration into BIAS software, each mask was tiled into 2048-pixel sections without overlap. A modified PIPΣX tiling feature ensured that the final tile in each row was extended to accommodate non-standard dimensions. The number of resulting tiles varied based on the original mask size.

### Contour processing in BIAS

Image files of Hoechst staining and tiled segmentation masks were uploaded into the software BIAS (Version 1.1.0, Single-Cell Technologies Ltd.). A data frame based on the imported segmentation mask was generated in BIAS containing cell area as well as x and y coordinates and exported to be aligned with the PIPΣX data frame matching cell IDs and incorporating an additional column containing the cluster number for each ID. The modified data frame was reimported into BIAS and clusters were selected individually to process contours. We dilated cell masks by half of the size of the laser impact line (3 µm) to improve target precision and merged (melded) some of the contours based on their spatial landscape to avoid sample loss (Supplementary Fig. 2c). The contours were modified by melding touching contours, closing sharp edges at 5 µm and expanding contour borders by 3 µm. The melding feature allowed to minimize the subsequent sample isolation time on the LMD.

Alignment points were placed in BIAS on peripheral nuclei and exported as an xml file containing cluster contour coordinates.

### Laser microdissection

For contour isolation, we used the Leica LMD7 system with software version 8.3.745. All contours were excised with a 63× objective and collected in low-binding 384 well plates (Eppendorf, Cat. No. 0030129547) at a temperature range from 19 °C to 23 °C. To document the precision of single-cell excision, dissection of individual annotated contours was recorded through the Leica LMD7 63× objective (Supplementary Movie 1).

Power and aperture were adjusted dynamically in the given range based on tissue topography and contour size, while larger contours were cut with increased power and aperture. Pulse frequency was decreased to 300 Hz when humidity reached below 30%. See Supplementary Table 3 for detailed laser settings. After each collection, contours were centrifuged at 2000 × *g* for 2 min.

### Tissue titration

To determine the pooled input that best balances protein yield with sample efficiency, we performed a contour-dilution titration. Pooled inputs of 100, 250, 500, 750, and 1000 contours (equivalent to 40, 100, 200, 300, and 400 cells) were processed in parallel; protein group identifications, inter-replicate coefficient of variation (CV), and inter-replicate Pearson correlation were quantified per bin. Based on this titration, 250 contours (~100 cell equivalents) per pooled MS injection were selected as the operating point representing the lowest input at which protein yield approaches saturation while inter-replicate CV remains below 10% (8.48%) and replicate Pearson correlation reaches ≥0.95 (0.950). Because protein yield is sensitive to the combination of tissue type and mass-spectrometer configuration, whenever any aspect of the workflow that can influence yield is changed, titration should be rerun.

### Sample preparation for mass spectrometry

Each sample consisted of 250 individually-cut, pooled contours (equivalent to the volume of ~100 cells) derived from cells of the same imaging-annotated subpopulation, isolated from multiple islets across the tissue section, regardless of their islet of origin. Samples were semi-automatically prepared on a Bravo pipetting robot (Agilent), similar to what was described previously^11,84,85^. Briefly, extracted contours were centrifuged at 2000 × *g* for 2 min, washed down with 7 µL 100% acetonitrile on each side of the well (in total 28 µL) and dried in a Vacuum concentrator (SpeedVac) at 45 °C for 20 min. Wells were visually quality controlled. In the following incubation steps, plates were tightly sealed with two stacked aluminum lids to avoid evaporation (Thermo Fisher Scientific, Cat. No. AB0626). The contours were resuspended in 6 µL lysis buffer (60 mM triethylammonium bicarbonate buffer (pH 8.5, Sigma) with 0.013% DDM (Sigma)), and heated for 60 min at 95 °C in a 384-well thermal cycler (Eppendorf Mastercycler) at a lid temperature of 110 °C. After the addition of 1 µL of 80% ACN (final concentration 10%), samples were incubated for 60 min at 75 °C and cooled briefly. Proteins were digested overnight at 37 °C using 4 ng LysC and 6 ng trypsin (1 μL in lysis buffer) at a lid temperature of 50 °C. The next day, digestion was stopped by adding trifluoroacetic acid (TFA, final concentration 1% v/v). Samples were loaded onto prepared Evotip Pure (Evosep) using the Bravo robot (Agilent).

### Peptide loading onto C-18 tips

C18 tips (Evotip Pure, Evosep) were activated in 1-propanol, washed two times with 50 µL buffer B (99.9% ACN, 0.1% FA), activated again in 1-propanol and washed twice with 50 µL buffer A (99.9% H2O, 0.1% FA). In between Evotips were spun at 700 × *g* for 1 min. Evotips were prepared with 70 µL buffer A and a short spin at 700 × *g* for sample loading. Samples were loaded into the remaining buffer A on the tip. Evotips were spun at 700 × *g* for 1 min to load the sample, washed with 50 µL buffer A and stored at 4 °C with buffer A on top until liquid chromatography–mass spectrometry (LC–MS) analysis.

### LC-MS/MS analysis

Samples were loaded onto Evotips Pure and measured with the Evosep One LC system (Evosep) coupled to an Orbitrap Astral mass spectrometer (Thermo Fisher Scientific). The Whisper40 SPD (samples per day) method was used on a commercial analytical column Aurora Elite TS of 15 cm length and 75 μm inner diameter (ID), packed with 1.7 μm C18 beads (IonOpticks). The column temperature was maintained at 50 °C using a column heater (IonOpticks). The mobile phases comprised 0.1% FA in LC–MS-grade water as buffer A and 99.9% ACN/0.1% FA as buffer B. The Orbitrap Astral mass spectrometer was equipped with a FAIMS Pro interface and an EASY-Spray source (both Thermo Fisher Scientific) at electrospray voltage of 1900 V. A FAIMS compensation voltage of −40 V and a total carrier gas flow of 3.5 L/min were used. The Orbitrap Astral was operated in DIA (data independent acquisition) mode with variable window widths at a positive spray voltage of 1900 V. The Orbitrap analyzer was utilized for full MS1 analyses with a resolution setting of 240,000 within a full scan range of 380–980 m/z. The automatic gain control (AGC) for the full MS1 was adjusted to 500% with a maximum injection time of 100 ms. The Astral analyzer was used for MS/MS acquisition with isolation windows of 6 Th (100 windows), a maximum ion injection time (IT) of 10 ms, an AGC target of 800%, and the MS/MS scanning range of 150–2000 m/z. Selected ions were fragmented by higher-energy collisional dissociation (HCD) at a normalized collision energy (NCE) of 25%.

The dilution series samples (Supplementary Fig. 4) were measured with the Evosep One LC system (Evosep) coupled to a timsTOF SCP mass spectrometer (Bruker). The Whisper20 SPD (samples per day) method was used on an Aurora Elite CSI third generation column with 15 cm length and 75 μm ID (AUR3-15075C18-CSI, IonOpticks). All other chromatography settings were the same as described above. The timsTOF SCP was operated in dia-PASEF mode with variable window widths. Optimal dia-PASEF methods cover the precursor cloud highly efficient in the m/z – ion mobility (IM) plane while providing deep proteome coverage. The optimal dia-PASEF method generated with py_diAID^86^ consisted of one MS1 scan followed by twelve dia-PASEF scans with two IM ramps per dia-PASEF scan, covering a m/z range from 350 to 1200 and IM of 0.7–1.3 Vs cm^−2^ ^84^. The mass spectrometer was operated in high sensitivity mode, the accumulation and ramp time was specified as 100 ms, capillary voltage was set to 900V and the collision energy was a linear ramp from 20 eV at 1/K0  =  0.6 Vs cm^−2^ to 59eV at 1/K0  =  1.6 Vs cm^−2^.

### Raw data analysis with DIA-NN

Orbitrap Astral raw files were first converted to the mzML file format using the MSConvert software (https://proteowizard.sourceforge.io/, version 3.0.23216) from Proteowizard, keeping the default parameters and selecting ‘Peak Picking’ as filter. Raw files (mzML or Bruker.d) were searched using the DIA-NN software^87,88^ (version 1.8.1) using the FASTA (2022, UP000005640_9606, with 20,137 gene entries) from the UniProt database and a library-free approach. A deep-learning module, match-between-runs (MBR) and heuristic protein inference (‘--relaxed-prot-inf’) was enabled. N-terminal methionine excision was set as fixed modification and oxidation as variable with maximum one allowed. ‘IDs, RT & IM profiling’ was used for library generation, ‘robust LC (high accuracy)’ for quantification and ‘RT-dependent’ for cross-run normalization. One miss cleavage was allowed and precursor charge range was set from 2 to 4. The false discovery rate (FDR) was controlled at less than 1% for peptide spectrum matches and protein group identifications. All other parameters were set as default. The ‘pg_matrix.tsv’ output file was used for further data analysis (Supplementary Data 4).

### Bioinformatic data analysis

All statistical analyses were performed using Perseus (v2.0.10), Python (v3.9.4 unless otherwise stated), and R (v4.2.2). Initial data processing included filtering out proteins from common contaminants, followed by log2 transformation.

For differential expression analysis, Welch’s t-test with a permutation-based false discovery rate (FDR) of 5% was conducted in Perseus. Enrichment analyses were performed using Fisher’s exact test with an FDR cutoff of 0.02, using the full dataset of 6,391 proteins as background. Supervised subtype-based analysis was performed using one-way ANOVA followed by post hoc tests, with a permutation-based FDR cutoff of 0.05 in Perseus. No imputation was applied for supervised comparisons.

Data visualization was carried out in Python using the seaborn package. Protein interaction networks were constructed using STRING output and visualized in Cytoscape (v3.8 and v3.10.3). One-dimensional (1D) annotation enrichment analysis was performed on score values using Perseus, applying a Benjamini–Hochberg FDR threshold of 0.05.

For principal component analysis (PCA), samples with fewer than 4000 identified proteins were excluded. Missing values were imputed using a normal distribution, downshifted by 1.9 standard deviations with a width of 0.5 relative to the original distribution.

Comparative analyses with annotated single-cell RNA sequencing (scRNA-seq) data were performed using datasets from Yoon et al.^59^ and Tabula Sapiens^89^. Gene expression values for the markers shown were extracted directly from published processed data for the annotated cell types used for comparison.

### Synthetic mixing analysis to assess polyhormonal cell authenticity

To evaluate whether polyhormonal proteomic signatures could arise from contamination by adjacent alpha and beta cells, we performed a computational mixing analysis. Alpha cell samples (*n* = 6; CHGA⁺GCG⁺ and CHGA⁺GCG⁺NPDC1⁺ populations) and beta cell samples (*n* = 4; CHGA⁺CPEP⁺ and CPEP⁺RBP4⁺ populations) were used as input to generate synthetic polyhormonal profiles. For each of three mixing ratios (alpha:beta = 25:75, 50:50, and 75:25), 1000 bootstrap replicates were generated by resampling alpha and beta samples with replacement, computing per-group means, and combining them at the specified ratio. This produced 1000 synthetic profiles per ratio, representing the expected proteomic signatures under a contamination scenario.

Real polyhormonal samples (*n* = 7; CHGA⁺CPEP⁺GCG⁺NPDC1⁺, CPEP⁺GCG⁺CHGA⁺, and CPEP⁺NPDC1⁺RBP4⁺ populations) were compared against synthetic profiles at each ratio. For each protein, an empirical *p*-value was computed as (*k* + 1)/(*n* + 1), where k is the number of synthetic replicates with a mean as extreme as or more extreme than the real polyhormonal mean, and *n* is the number of bootstrap replicates. Mann–Whitney *U* tests were performed as a complementary non-parametric assessment. *P*-values were corrected for multiple testing using the Benjamini–Hochberg method (FDR < 0.05). Proteins significant across all three mixing ratios were identified as consistently differential.

For PCA visualization, proteins present in at least 50% of samples were retained, and missing values were imputed using K-nearest neighbors (KNN) imputation (*k* = 5, scikit-learn v1.x). A subset of 50 synthetic profiles per ratio were projected onto the PCA space fitted on real samples. Volcano plots were generated using Mann–Whitney *U* test FDR values with proportional jitter applied to the y-axis to mitigate banding from discrete rank-based *p*-values. Gene labels were positioned using the adjustText library to avoid overlap.

### Cross-omics comparison with single-cell transcriptomics

Comparative analyses between mxDVP proteomic measurements and single-cell RNA-seq data were performed using an integrated reference dataset comprising the Yoon et al. human pancreatic islet atlas^59^ and the pancreatic compartment of Tabula Sapiens^89^ tab, collectively spanning 52 donors. Both datasets were generated using 10× Chromium and processed through standard scRNA-seq pipelines as described in the original publications. Pre-processed and annotated data were retrieved from CellxGene Discover.

For each endocrine cell type annotation in the reference scRNA-seq data (alpha, beta, delta, PP, polyhormonal), pseudobulk expression profiles were generated by summing transcript counts across all cells of that type, followed by normalization to counts-per-million and log-transformation (log₂(CPM + 1)). Polyhormonal cells in the scRNA-seq reference were identified as cells expressing two or more hormone transcripts (INS, GCG, SST, PPY) above background, with the specific co-expression criterion applied during annotation as described in the source publications.

### Cross-omics visualization

Markers visualized in Fig. 5d (mxDVP proteomics) and Fig. 5e (scRNA-seq) were selected to span canonical endocrine markers, secretory machinery components, and polyhormonal-associated proteins. Both panels were plotted with the same marker order on the y-axis and matched cell-type columns to enable direct visual comparison, using a consistent color scheme across panels. Expression values were z-scored per marker across cell types, such that dot color reflects the relative expression of each marker between cell types (blue, lower; red, higher). For Fig. 5d, color represents the z-scored median log₂ protein abundance per cell-type pool, and dot size represents the proportion of MS samples within that pool with detectable signal. The median was used because it is robust to the limited sample number and missing values inherent to single-cell proteomics. For Fig. 5e, color represents the z-scored mean log-normalized expression, consistent with standard scRNA-seq dot-plot conventions and the original data source (GSE196715), and dot size represents the proportion of cells with detectable transcript. Both SCG3 isoforms are displayed separately in the proteomic panel, whereas the transcriptomic dataset quantifies a single SCG3 gene.

### Statistical testing

For markers of interest at the protein level (SCG3, INSM1), Kruskal–Wallis tests were applied to compare protein abundance across the four imaging-defined cell-type pools (alpha, beta, beta-with-SST, polyhormonal) in the mxDVP data. *P*-values were not adjusted for multiple testing given the small number of pre-specified markers tested. Equivalent Kruskal–Wallis tests were applied to the matched scRNA-seq subtypes for the same markers. All analyses were performed in Python using scipy.stats.kruskal and visualizations were generated using scanpy.pl.dotplot (scRNA-seq) and custom matplotlib code (proteomics).

### mRNA–protein correlation analysis

For cross-platform comparison, we used genes quantified in both the mxDVP proteomic dataset and the single-cell transcriptomic dataset (Lynn Lab, GSE196715/Yoon et al.; *n* = 30 shared genes). Transcriptomic per-cell-type average expression was extracted from the processed Seurat object using log-normalized RNA-assay values. Proteomic intensities (log₂) were exponentiated to linear scale and averaged per cell type across single cells. Two normalization approaches were applied: (i) internal normalization over the sum of the shared gene set followed by log₁₀ transformation, and (ii) per-gene z-scoring across cell types. Beta-with-SST proteomic cells were combined with β cells; delta and PP cells were excluded owing to the lack of reliable cross-platform cell-type correspondence. Spearman rank correlations were computed per cell type and pooled across cell types. Genes with an absolute inter-modality difference >1 (log₁₀ or z-score units) were classified as discordant. Analyses were performed in Python (pandas, numpy, scipy).

### Reporting summary

Further information on research design is available in the Nature Portfolio Reporting Summary linked to this article.

## Supplementary information

Supplementary Information

Description of Additional Supplementary Files

Supplementary Data 1

Supplementary Data 2

Supplementary Data 3

Supplementary Data 4

Supplementary Data 5

Supplementary Movie 1

Reporting Summary

Transparent Peer Review file

## Source data

Source Data

## Data Availability

All supplementary tables and supplementary data associated with this study are available at Zenodo: https://doi.org/10.5281/zenodo.21726555. The high-plex image data is available in EBI BioImage Archive (https://www.ebi.ac.uk/biostudies/BioImages/studies) with the accession number S-BIAD1853 https://doi.org/10.6019/S-BIAD1853. All mass spectrometry raw files and proteomics data analyzed in this study have been deposited to the ProteomeXchange Consortium via the PRIDE partner repository^90^, project accession PXD062166. The deposited dataset additionally includes mass spectrometry data from a third donor that was part of the combined database used for peptide and protein identification; all analyses reported in this study are based on the two donors (R322 and R348) described in Supplementary Table 1. Source data are provided with this paper.

## Code availability

All code for image preprocessing, segmentation, postprocessing and analysis associated with current submission is provided as a part of the initial PIPΣX release (version 1.0). And also available at Zenodo: https://doi.org/10.5281/zenodo.1164237592^91^ And the current version of the software is available at https://github.com/CellProfiling/pipex under the MIT license. The special standalone PIPΣX-BIAS integration script is available at https://github.com/CellProfiling/pipex/tree/main/extra under the MIT license.
