# Supplementary material for: Multiplexed deep visual proteomics resolves spatial heterogeneity and rare endocrine states in human pancreatic islets
Source: Nat Commun. 2026 Sep 25;17:11138. doi: 10.1038/s41467-026-77890-6 (PMC13710848; doi:10.1038/s41467-026-77890-6)
Supplement: Supplementary file 1 [file 41467_2026_77890_MOESM10_ESM.pdf]

nature portfolio

Peer Review File

Multiplexed Deep Visual Proteomics Resolves Spatial Heterogeneity and Rare Endocrine States in Human Pancreatic Islets

Corresponding Author: Professor Emma Lundberg

This file contains all reviewer reports in order by version, followed by all author rebuttals in order by version.

Version 0:

Reviewer comments:

Reviewer #1

(Remarks to the Author)
The authors have invested substantial work in this revision and the manuscript is improved. I appreciate the technical advance presented here, which I think would be relevant to spatial biology studies. The specific finding of polyhormonal cells though is still not convincing. I understand there are technical limitations in setting up smFISH for this particular study, but since this is the ultimate proof for existence of polyhormonal cells and since this is a controversial topic and the evidence presented here is not 100%, the authors must tone down the discussion of this finding.

Specific comments:

- Comments 1.4 - The computational mixing and the independent CODEX datasets are supportive but still not conclusive. The logic of the comparison with Wang et al, who used thinner sections is inverse, the thicker the section the higher the chance to capture signals from stacked cells. The possibility of stacked cells contributing to the polyhormonal feature identified here must be noted in the Discussion it should be stated that future studies using sensitive in-situ single molecule transcript imaging, ideally on dissociated cells, could unambiguously prove their existence.

- Response to comment 1.6 - "We agree that direct smFISH/IF validation specifically for SCG3/INSM1 in our donor tissues would strengthen these findings further; this is acknowledged as a future direction in the revised Discussion." I did not find this written in the Discussion, please explicitly spell out where this was added.

- Comment 1.7 - Figure panels 5e,f are very hard to interpret, please add a scatter plot of the log-expression of proteins vs. the log-expression of mRNAs (both normalized internally over the sum of the set of genes that exist in both mRNA and protein measurements). Please provide Spearman correlation coefficient and highlight genes that are higher in either the mRNA or protein measurements.

- Comment 1.7 - Single cell studies are not suited for identifying polyhormonal cells, fractured cells release RNA that attaches to the outside of all cells, this is why genes like INS appear in all cell types in pancreatic single cell RNAseq atlases, including in immune and mesenchymal cells. This cannot be considered an independent validation for the existence of polyhormonal cells.

Reviewer #2

(Remarks to the Author)
The authors have performed thorough and thoughtful revisions to their manuscript, which substantially recalibrated the biological interpretation to reflect the limited cohort size and now clearly acknowledges that a direct comparison with diseased tissue will be required to support broader clinical or disease-related conclusions.
I appreciate the expanded analyses and validation surrounding the polyhormonal cell populations. The combination of orthogonal datasets, donor-level reporting, and the synthetic mixing analyses provides a much stronger framework for evaluating these rare populations. I believe the computational mixing strategy is a particularly valuable addition that will be

useful beyond the present study and could serve as a helpful benchmark for future deep visual proteomics studies investigating rare or mixed cellular populations.
Finally, the authors have substantially improved the presentation, documentation, and accessibility of the PIPΣX workflow, including the addition of tutorial materials and resources, which should facilitate broader adoption by the community. Overall, I find that my major concerns have been satisfactorily addressed and I have no further substantive comments.

Reviewer #3

(Remarks to the Author)
The author did a great job addressing the comments from all three reviewers. It is clear that the improvement of the DVP workflow and its application to islet cells justifies its publication in Nature Communications.

I read through all the responses and am satisfied with most of them. However, I still cannot find evidence demonstrating the precision of cell segmentation and the laser dissection workflow.

Comment 3.4, Cell segmentation precision and laser dissection. The authors point out that "We provide fluorescence imaging of islet tissue before and after laser microdissection of annotated contours" in Extended Data Fig. 4c. However, Extended Data Fig. 4c is a low-magnification bright-field image of whole islet dissection before/after laser dissection. Such low magnification cannot really assess the precision of single-cell dissection.

I would like to see clear evidence to show the precision of single-cell dissection using fluorescence images, as the author pointed out.

Open Access This Peer Review File is licensed under a Creative Commons Attribution 4.0 International License, which permits use, sharing, adaptation, distribution and reproduction in any medium or format, as long as you give appropriate credit to the original author(s) and the source, provide a link to the Creative Commons license, and indicate if changes were made.
In cases where reviewers are anonymous, credit should be given to 'Anonymous Referee' and the source.
The images or other third party material in this Peer Review File are included in the article’s Creative Commons license, unless indicated otherwise in a credit line to the material. If material is not included in the article’s Creative Commons license and your intended use is not permitted by statutory regulation or exceeds the permitted use, you will need to obtain permission directly from the copyright holder.
To view a copy of this license, visit https://creativecommons.org/licenses/by/4.0/

Point-by-Point Response to Reviewers

Multiplexed Deep Visual Proteomics Resolves Spatial Heterogeneity and Rare Endocrine States in Human Pancreatic Islets

We thank the reviewers for the constructive final assessment. Below we respond to each remaining comment. Reviewer comments are reproduced in italics; new or revised manuscript text is shown in blue, with its location in the revised manuscript specified in each case. Following the editorial request, all items previously presented as Extended Data Figures 1-10 are now Supplementary Figures 1-10, and figure citations below use the revised numbering.

Reviewer #1

We are grateful to Reviewer 1 for continued rigorous engagement with the polyhormonal question. We agree that our evidence, while extensive, is not definitive, and we have recalibrated the manuscript accordingly. We have removed the argument based on section thickness, which the reviewer correctly identifies as running in the wrong direction; added an explicit statement of the stacked-cell limitation to the Discussion; removed the claim that single-cell RNA-seq provides independent validation of polyhormonal cells; and added the requested protein-versus-transcript scatter plot. Polyhormonal language has been toned down throughout the Abstract, Results and Discussion, and the title has been changed from "Unveils" to "Resolves".

Comment 1.4 (follow-up), Stacked cells and the direction of the section-thickness argument

> *Reviewer: The computational mixing and the independent CODEX datasets are supportive but still not conclusive. The logic of the comparison with Wang et al, who used thinner sections is inverse, the thicker the section the higher the chance to capture signals from stacked cells. The possibility of stacked cells contributing to the polyhormonal feature identified here must be noted in the Discussion it should be stated that future studies using sensitive in-situ single molecule transcript imaging, ideally on dissociated cells, could unambiguously prove their existence.*

Response: The reviewer is right and we thank them for catching this. A thicker section increases, rather than decreases, the opportunity for axially stacked cells to contribute overlapping signal, so our previous use of the 8-micron section thickness as an argument against stacking was logically inverted. We have removed that argument and made three changes.

1. Results, section "Exploring the spatial proteome of rare and bihormonal endocrine cell populations". The sentence that presented 8-micrometre sectioning as evidence against a stacked-cell contamination scenario has been deleted. The remaining points, membrane-aware segmentation constrained to individual nuclei and fluorescence imaging before and

after laser microdissection, are now presented as measures that reduce, not exclude, adjacent-cell and stacked-cell contribution.

2. Discussion, paragraph 1. The passage previously stating that our approach "directly addresses this limitation" now reads that our approach "differs from IMC in ways that constrain, but do not fully eliminate, this ambiguity."

3. Discussion. A new limitation paragraph has been added in full, immediately following the Wang et al. comparison.

New Discussion paragraph (Discussion, following the Wang et al. comparison):

A limitation of any section-based imaging approach, including ours, is that cells stacked along the axial dimension can contribute to an overlapping signal within a single optical section. Our tissue sections were cut at 8 micrometres, which is thinner than the reported diameter of a human endocrine cell (approximately 9-13 micrometres). A section thinner than a cell body precludes the capture of two fully overlapping cells within a single plane, but it does not exclude partial axial overlap between the upper and lower portions of vertically adjacent cells, and this residual possibility cannot be ruled out on the basis of section thickness alone. Membrane-aware segmentation constrained to individual nuclei, and fluorescence imaging of the tissue before and after laser capture microdissection, reduce the contribution of adjacent cells, and the synthetic mixing analysis (Supplementary Fig. 9c-d) shows that the polyhormonal proteomes are not reconstructable as linear combinations of alpha- and beta-cell proteomes. Together these observations argue against simple two-cell stacking as the sole origin of the signal, but they do not exclude a contribution of stacked cells to the polyhormonal feature we describe. Definitive demonstration that individual cells co-express multiple hormones will require sensitive in situ single-molecule transcript imaging, ideally performed on dissociated cells, where the axial ambiguity inherent to tissue sections is removed. We regard the populations described here as protein-level co-expression states that are consistent with, but not proof of, genuine polyhormonal cells.

We note for completeness that polyhormonal cells are a documented feature of human endocrine biology in adjacent settings, arising as a transitional population during human pancreatic development and during *in vitro* differentiation of stem cell derived islets, and re-emerging in the context of beta-cell dedifferentiation and metabolic stress. We do not present this literature as validation, since it cannot resolve the axial ambiguity in our data. We cite it only to indicate that co-expression of multiple hormones by a single human endocrine cell is a biologically plausible state rather than an a priori artefact.

Comment 1.6 (follow-up), Location of the smFISH/IF future-direction statement

Reviewer: I did not find this written in the Discussion, please explicitly spell out where this was added.

Response: The reviewer is correct that this statement was absent from the submitted version. The sentence had been drafted in our previous response letter but was not carried into the

manuscript file, and we apologise for the oversight. It has now been added, and we specify its location precisely below.

Location: Discussion, in the paragraph beginning "Furthermore, the discovery of distinct endocrine subtypes suggests that cellular heterogeneity within islets extends beyond classical alpha-, beta-, and delta-cell categorizations". The new text forms the final three sentences of that paragraph.

New text (Discussion, final three sentences of the paragraph identified above):

> The proteins that define these populations, in particular SCG3 and INSM1, have not been validated in our own donor tissues by an orthogonal in situ method. Direct smFISH or immunofluorescence for SCG3 and INSM1 in the same tissue, combined with in situ single-molecule transcript imaging on dissociated cells, would establish both the cellular specificity of these markers and the existence of individual cells co-expressing multiple hormones. We regard this as the necessary next step for these populations.

This statement is also cross-referenced from the stacked-cell limitation paragraph added in response to comment 1.4.

Comment 1.7 (follow-up, first part), Interpretability of the dot plots and the requested scatter plot

> *Reviewer: Figure panels 5e,f are very hard to interpret, please add a scatter plot of the log-expression of proteins vs. the log-expression of mRNAs (both normalized internally over the sum of the set of genes that exist in both mRNA and protein measurements). Please provide Spearman correlation coefficient and highlight genes that are higher in either the mRNA or protein measurements.*

Response: We agree that the paired dot plots are difficult to read against each other, and we have added the requested scatter plot. The analysis was performed exactly as specified. Note that following the editorial reorganisation of Figure 5, the dot plots are now panels 5d (protein) and 5e (transcript), and the new scatter plot is panel 5f.

Method. We restricted the analysis to the 30 features measured in both modalities. For each cell type, protein intensities and transcript counts were each normalised internally over the sum of that shared feature set and then log-transformed, so that both axes represent the fraction of shared-set signal attributable to each feature within that cell type. Each point is one gene in one endocrine cell type. We report Spearman rank correlation coefficients with exact two-sided p values. Features deviating from the diagonal beyond a defined residual threshold are highlighted and coloured by protein class, so that features higher in the protein measurement and features higher in the transcript measurement are separately identified. Because not all populations had a reliable one-to-one cross-platform correspondence, the correlation was restricted to alpha, beta and polyhormonal cells; beta-with-SST proteomic cells were grouped with beta cells, and delta and PP cells were excluded. This is stated in the Results and Methods.

Results. Protein and transcript abundance were positively correlated in every matched cell type: Spearman rho = 0.61 (p = 3.4e-04) for alpha, 0.45 (p = 1.2e-02) for beta and 0.46 (p = 9.7e-03) for polyhormonal cells, with a pooled rho of 0.51 (p = 2.5e-07, n = 90). The pooled scatter is shown as Figure 5f. The per-cell-type scatters, together with an equivalent analysis after per-gene z-scoring, are provided as Supplementary Fig. 10d, so that the reviewer can inspect both normalisations. Panels 5d and 5e are retained because they show the per-cell-type expression and detection structure that the scatter plot collapses, but the interpretation in the Results now leads with the scatter plot.

New Figure 5 legend panel f:

> f) Correlation between protein and transcript abundance across the shared feature set (n = 30 genes), each normalised internally over the sum of the shared set and log10-transformed, pooled across alpha, beta and polyhormonal cell types (Spearman rho = 0.51, two-sided p = 2.5 x 10-7, n = 90). Points are coloured by concordance and, where discordant, by protein class: secreted (blue), stable/membrane (red), other (grey); concordant features are shown in light grey. Discordance was defined as an absolute inter-modality difference greater than 1 log10 unit. The green line indicates the linear fit. The dashed line indicates the identity relationship and is shown for reference only, as the two modalities are normalised independently and are not on a common absolute scale. Source data are provided as a Source Data file.

The genes departing most from overall concordance did so in a directionally interpretable way, and this is now stated in the Results: secreted peptides tended to show higher relative transcript abundance, consistent with regulated secretion depleting the intracellular protein pool sampled by MS, whereas stable and membrane-associated proteins (GP2, ALDH1A1, ALDH1B1) showed higher relative protein.

Comment 1.7 (follow-up, second part), Single-cell RNA-seq cannot validate polyhormonal cells

> *Reviewer: Single cell studies are not suited for identifying polyhormonal cells, fractured cells release RNA that attaches to the outside of all cells... This cannot be considered an independent validation for the existence of polyhormonal cells.*

Response: We accept this point in full. Ambient RNA released from damaged cells during dissociation adsorbs onto intact cells, so hormone transcript co-detection cannot distinguish a genuinely polyhormonal cell from a monohormonal cell carrying ambient transcript. We note that this is a distinct artefact from cell doublets and is not addressed by doublet filtering, since a singlet carrying ambient transcript passes doublet detection. We have removed every claim in the manuscript that used single-cell transcriptomics as validation.

> 1. Discussion, paragraph 1. The clause presenting the meta-analysis across 18 scRNA-seq datasets and over 266,000 cells as independent identification of a polyhormonal transcriptional trajectory has been deleted. The supporting evidence in that sentence is now limited to protein-level and imaging-level evidence.

2. Results, cross-omics section. The sentence stating that the absence of the polyhormonal protein signature at transcript level argued against artifactual cell mixing in scRNA-seq (doublets) has been removed. We accept that if ambient contamination inflates apparent co-detection, the absence of the signature at transcript level cannot be interpreted in either direction.

3. Results. We have also corrected a factual error in the previous version. The manuscript previously stated that the polyhormonal protein signature was "not detectable at the transcriptomic level". This was incorrect: SCG3, INSM1 and ALDH1A1 are all detected in the transcriptomic data. The accurate statement, now in the manuscript, is that their protein enrichment is not accompanied by a corresponding transcript enrichment in the same cell type.

New text (Results, end of the cross-omics section):

We note that the cross-platform comparison reflects concordance of relative expression patterns between independent datasets rather than a sample-matched comparison, and that dissociated single-cell RNA-seq is not by itself a definitive test of hormone co-expression; the transcriptomic data are therefore used here to compare relative feature abundance between modalities rather than as independent validation of these populations.

Reviewer #2

Reviewer: Overall, I find that my major concerns have been satisfactorily addressed and I have no further substantive comments.

Response: We thank Reviewer 2 for the careful review across all rounds and for the suggestions that shaped the revision, in particular the synthetic mixing strategy and the donor-level reporting. No further changes are required in response to this review. We note that the recalibration performed in response to Reviewer 1 in this round further strengthens the framing that Reviewer 2 asked us to adopt.

Reviewer #3

Comment 3.4 (follow-up), Precision of single-cell segmentation and laser dissection

Reviewer: Extended Data Fig. 4c is a low-magnification bright-field image of whole islet dissection before/after laser dissection. Such low magnification cannot really assess the precision of single-cell dissection. I would like to see clear evidence to show the precision of single-cell dissection using fluorescence images, as the author pointed out.

Response: The reviewer is right, and we apologise for the mismatch between our previous response and the panel we cited. That panel shows whole-islet dissection at low magnification in

bright field and does not allow the precision of single-cell dissection to be assessed. We have addressed this with new primary data.

1. Supplementary Movie 1, single-cell laser capture. We provide a video recording of the laser microdissection of individual annotated contours, acquired on the Leica LMD7 during a dissection run of this study. The movie shows the contour outline as imported from PIPΣX, the laser path following the contour, the excision of the single cell, and the tissue immediately after capture, so that the precision and quality of the cut can be assessed directly rather than through a summary panel. The movie is submitted as a separate supplementary file and is cited in the Results and in the Methods.

2. High-magnification brightfield before and after panels. New panels have been added to Supplementary Figure 4c showing the same field at high magnification before and after dissection of individually annotated single-cell contours, with the segmentation mask overlaid. The panels show that the excised area corresponds to the annotated contour and that neighbouring cells remain intact.

New Results text (Results, mxDVP workflow section, following the description of contour transfer):

Within our own dataset, several methodological features further argue against a stacked-cell contamination scenario as described by Wang et al43, tissue sections were cut at 8 µm thickness with membrane-aware watershed segmentation constraining cell boundaries to individual nuclei, and fluorescence imaging before and after laser capture microdissection confirmed precise contour isolation with minimal adjacent-cell signal (Supplementary Fig. 4c; Supplementary Movie 1).

New Methods text (Methods, Laser microdissection subsection):

To document the precision of single-cell excision, dissection of individual annotated contours was recorded through the Leica LMD7 63x objective (Supplementary Movie 1).

Summary of changes made in this round

Manuscript text

1. Removed the section-thickness argument against stacked-cell contamination from the Results and corrected the corresponding Discussion passage (R1, 1.4).

2. Added an explicit stacked-cell limitation paragraph to the Discussion, including the recommendation for in situ single-molecule transcript imaging on dissociated cells (R1, 1.4).

3. Added the smFISH/immunofluorescence future-direction statement for SCG3 and INSM1 to the Discussion, with its location specified (R1, 1.6).

4. Removed the use of single-cell RNA-seq as independent validation from the Discussion and Results, corrected the inaccurate "not detectable" statement, and added an explicit caveat (R1, 1.7).

5. Toned down polyhormonal language throughout, and changed the title from "Unveils" to "Resolves".

Display items

6. New Figure 5f, pooled scatter of log-normalised protein versus transcript abundance across the shared feature set, with Spearman correlation and directional highlighting (R1, 1.7).

7. New Supplementary Fig. 10d, per-cell-type correlations under both sum-normalised and z-scored normalisations (R1, 1.7).

8. New Supplementary Movie 1, single-cell laser microdissection capture (R3, 3.4).

9. Extended Data Figures 1-10 renumbered as Supplementary Figures 1-10 per the editorial request; Figure 4 and Figure 5 panel lettering revised accordingly.
