# Supplementary material for: Multiplexed deep visual proteomics resolves spatial heterogeneity and rare endocrine states in human pancreatic islets
Source: Nat Commun. 2026 Sep 25;17:11138. doi: 10.1038/s41467-026-77890-6 (PMC13710848; doi:10.1038/s41467-026-77890-6)
Supplement: Supplementary file 2 [file 41467_2026_77890_MOESM1_ESM.pdf]

Supplementary Figure 1

a

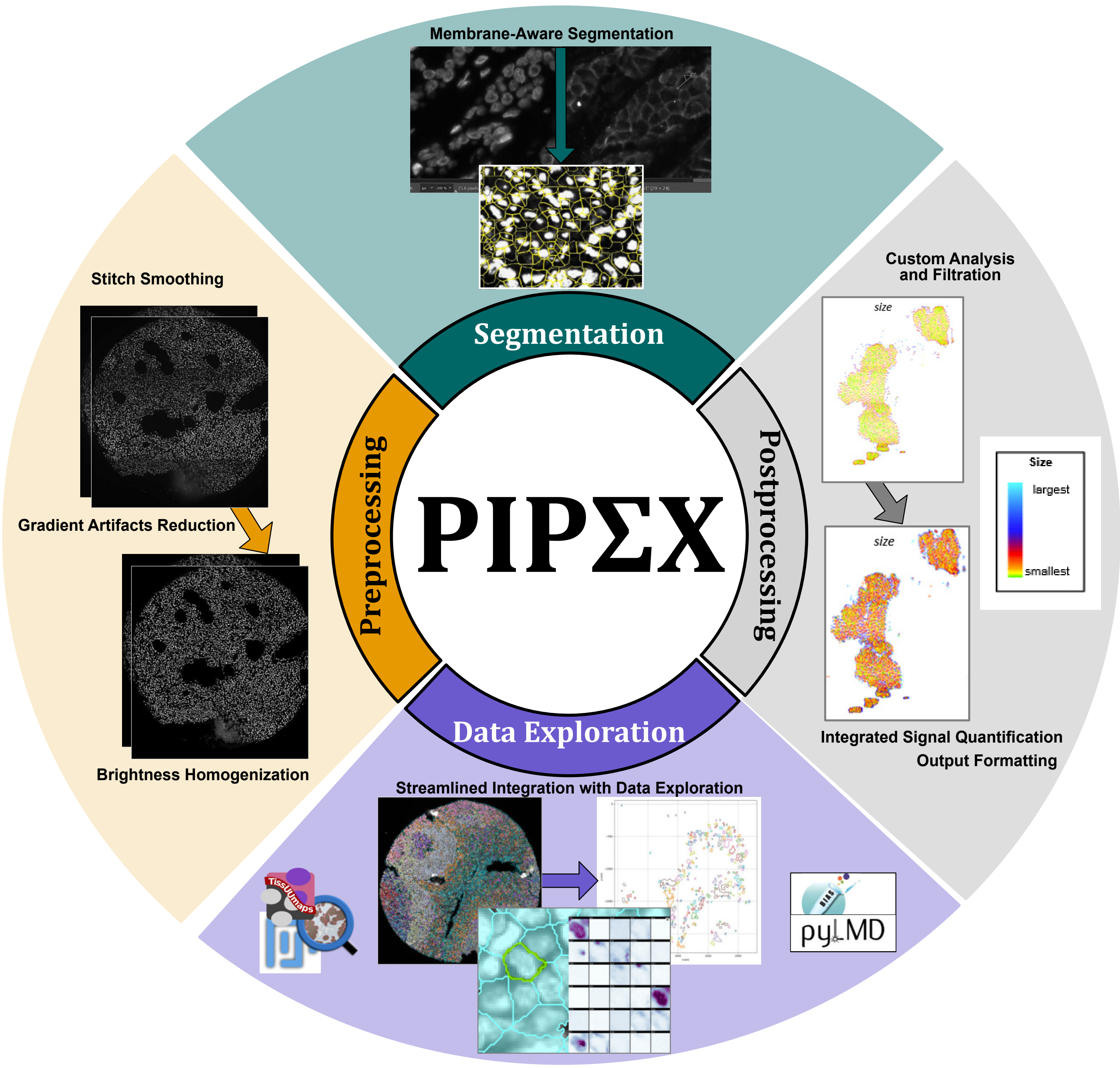

b

Tissue	Type	Tissue section	Number of markers	Total runtime	Number of cells identified
Normal	Salivary gland	Tissue Microarray	49 markers	11 minutes	8938 cells
Normal	Spleen	Tissue Microarray	49 markers	14 minutes	16587 cells
Normal	Tonsil	Tissue Microarray	17 markers	15 minutes	20063 cells
Normal	Thyroid gland	Tissue Microarray	49 markers	9 minutes	6022 cells
Normal	Vagina	Tissue Microarray	49 markers	8 minutes	2578 cells
Cancer	Breast cancer	Whole tissue section	10 markers	89 minutes	257757 cells
Cancer	Gastric cancer	Tissue Microarray	49 markers	13 minutes	9828 cells
Cancer	Pancreatic cancer	Tissue Microarray	49 markers	10 minutes	5441 cells
Cancer	Hepatic cancer	Tissue Microarray	49 markers	13 minutes	10862 cells

Supplementary Figure 1: PIPΣX software capabilities and flexibility with the possibility of independent use of different modules.

a) Overview of the PIPΣX pipeline, illustrating its modular design for flexible and scalable image analysis across diverse tissue types. Each component, preprocessing, segmentation, clustering, visualization, and export, can be run independently or as part of an end-to-end automated workflow. The modular structure supports both standalone use of individual steps and integration into broader spatial analysis pipelines, enabling customization for user-specific applications such as clustering, cell-type annotation, spatial context exploration, or export for laser microdissection (LMD). All schematic elements in this figure were created by the authors using Affinity Designer (Serif). Microscopy and tissue images within the figure were acquired by the authors from the tissue sections analyzed in this study. No generative AI was used to create any element. b) Summary table of the PIPΣX performance across diverse tissue types and biological states. The table reports the number of cells segmented and total runtime per image, highlighting the scalability and consistency of the PIPΣX. Tissue types include both normal and diseased states, spanning epithelial and immune-rich environments.

Supplementary Figure 2

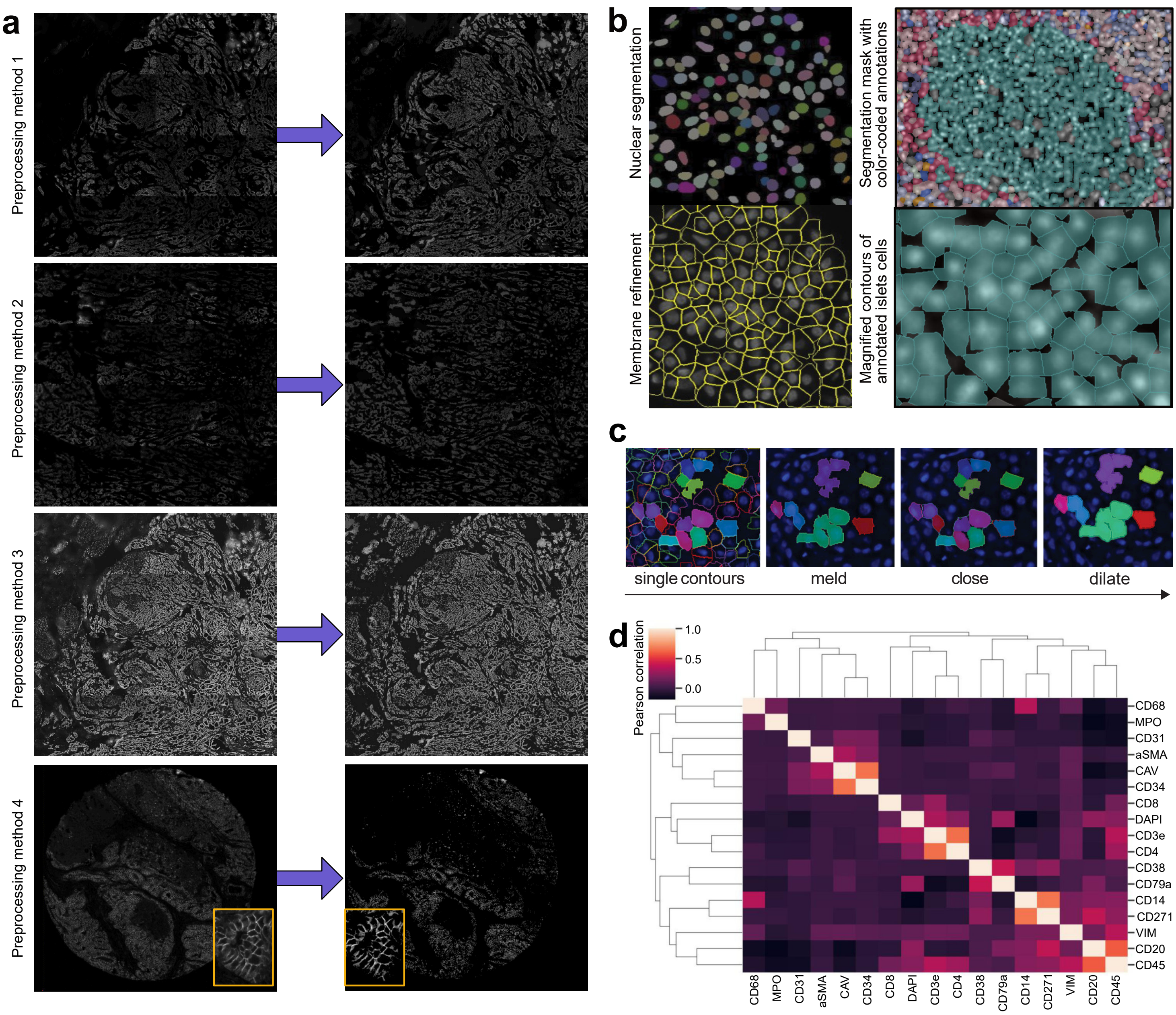

Supplementary Figure 2: PIPΣX-enabled clustering refinement, contour optimization, and workflow validation for spatial proteomics.

a) Representative examples of raw and preprocessed multiplexed images demonstrate the impact of automated correction on image quality. Breast cancer whole-tissue section exhibiting uneven illumination and visible tile boundaries. Preprocessing parameters: threshold minimum = 1, bright levels = 5:1:2, light gradient correction = 2, exposure = 200. b) Example images from the in-house developed image analysis pipeline (PIPΣX), showcasing the nuclear segmentation by StarDist, membrane refinement feature for improved segmentation accuracy, and the full cell segmentation masks for a representative islet. c) Representative images showcasing the developed post-adjustment tool where the exported cell segmentation masks are (1) melded based on assigned identified cell type and spatial organization, (2) gaps between the melded cell contours are closed, and (3) dilated to account for the laser impact. d) Heatmap of marker co-expression across cells on the example of a non-diseased tonsil. Pearson correlation highlights relationships among immune and structural markers.

Supplementary Figure 2

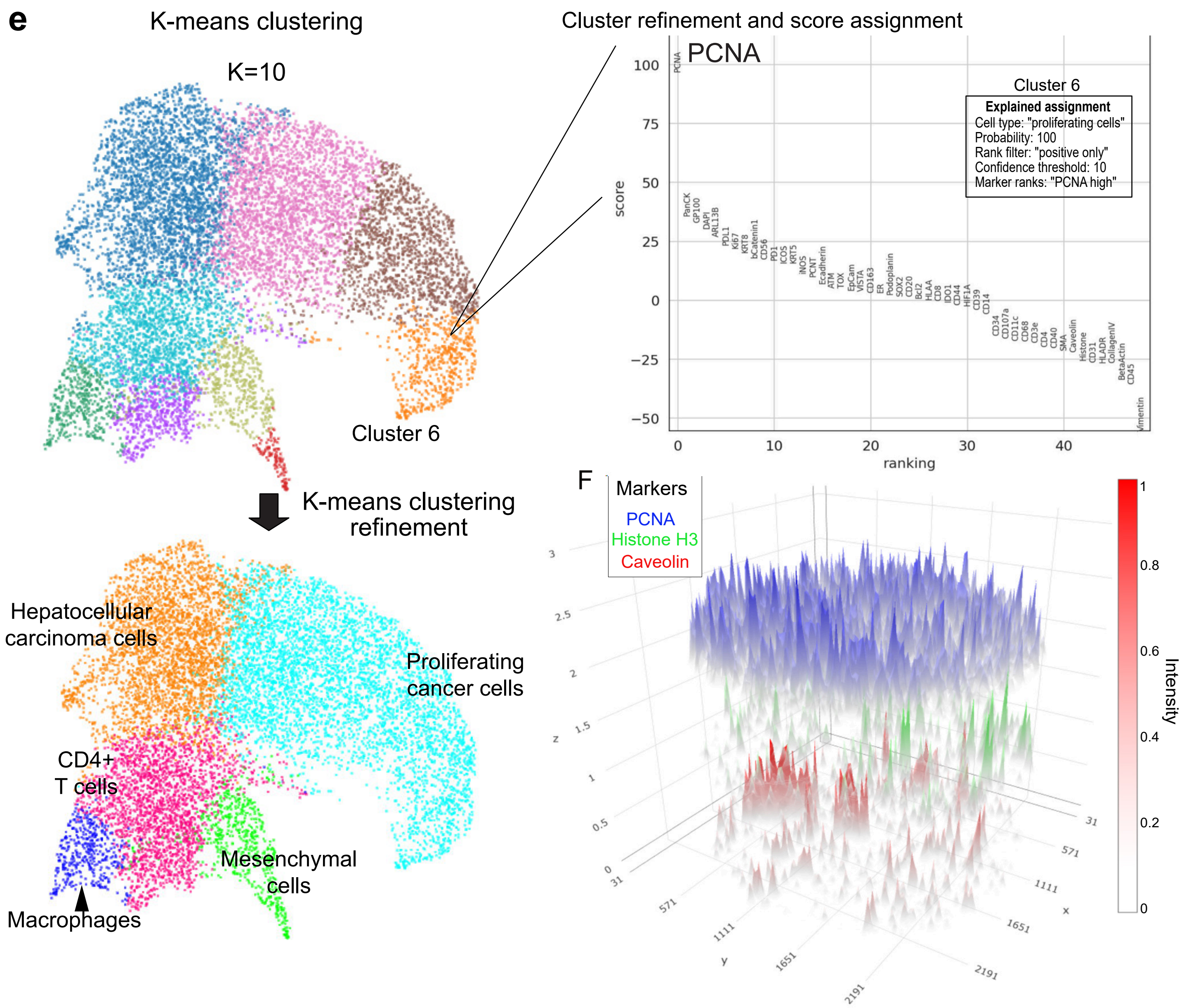

Supplementary Figure 2: PIPΣX-enabled clustering refinement, contour optimization, and workflow validation for spatial proteomics.

e) UMAP embedding of all segmented cells color-coded by K-means clustering output (K = 10, top) and refined clustering output generated using PIPΣX cluster refinement algorithm (bottom) with assignment scores based on marker-specific contributions (right). f) Surface marker plots illustrating expression levels of PCNA, Histone H3, and Caveolin in spatial coordinates (x, y, z), visualizing spatially resolved molecular gradients of marker expressions.

Supplementary Figure 3

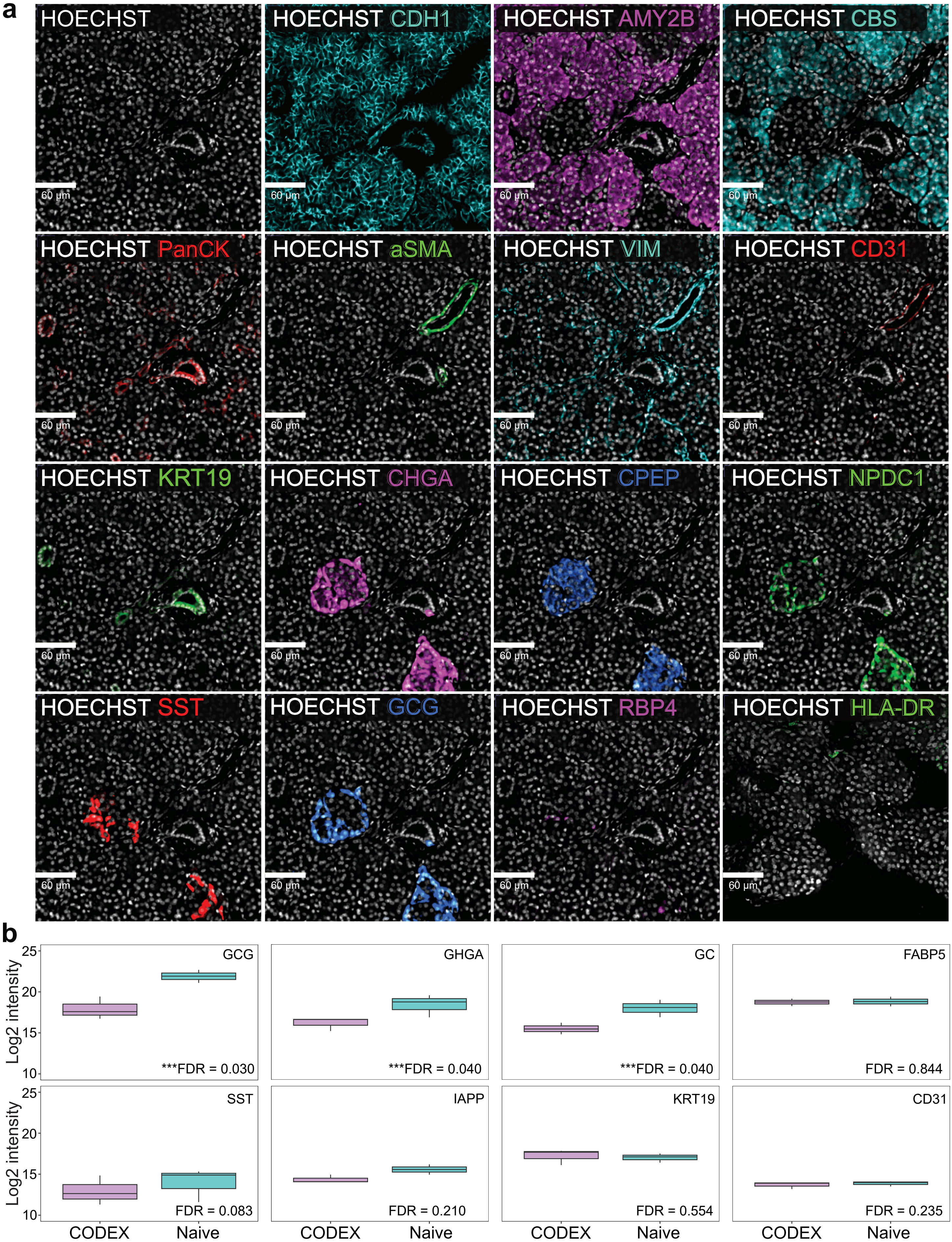

Supplementary Figure 3: Proteomic and spatial analysis reveal endocrine cell state heterogeneity.

a) Representative high-plex images from the custom-developed antibody panel showcasing the immunohistological staining of the markers of different islet cells and surrounding cellular components of the pancreatic tissue microenvironment (scale bar is set at 60 μm). Images organized based on functional categories: exocrine (CBS, AMY2B), ductal (PanCK, KRT19), vascular (VIM, CD31, αSMA), hormone-secreting (CHGA, GCG, NPDC1, RBP4, CPEP, SST), and immune (HLA-DR). b) Bar plots showing influence of CODEX method on marker abundance across specific markers compared to naive tissue by mass spectrometry.

Supplementary Figure 4

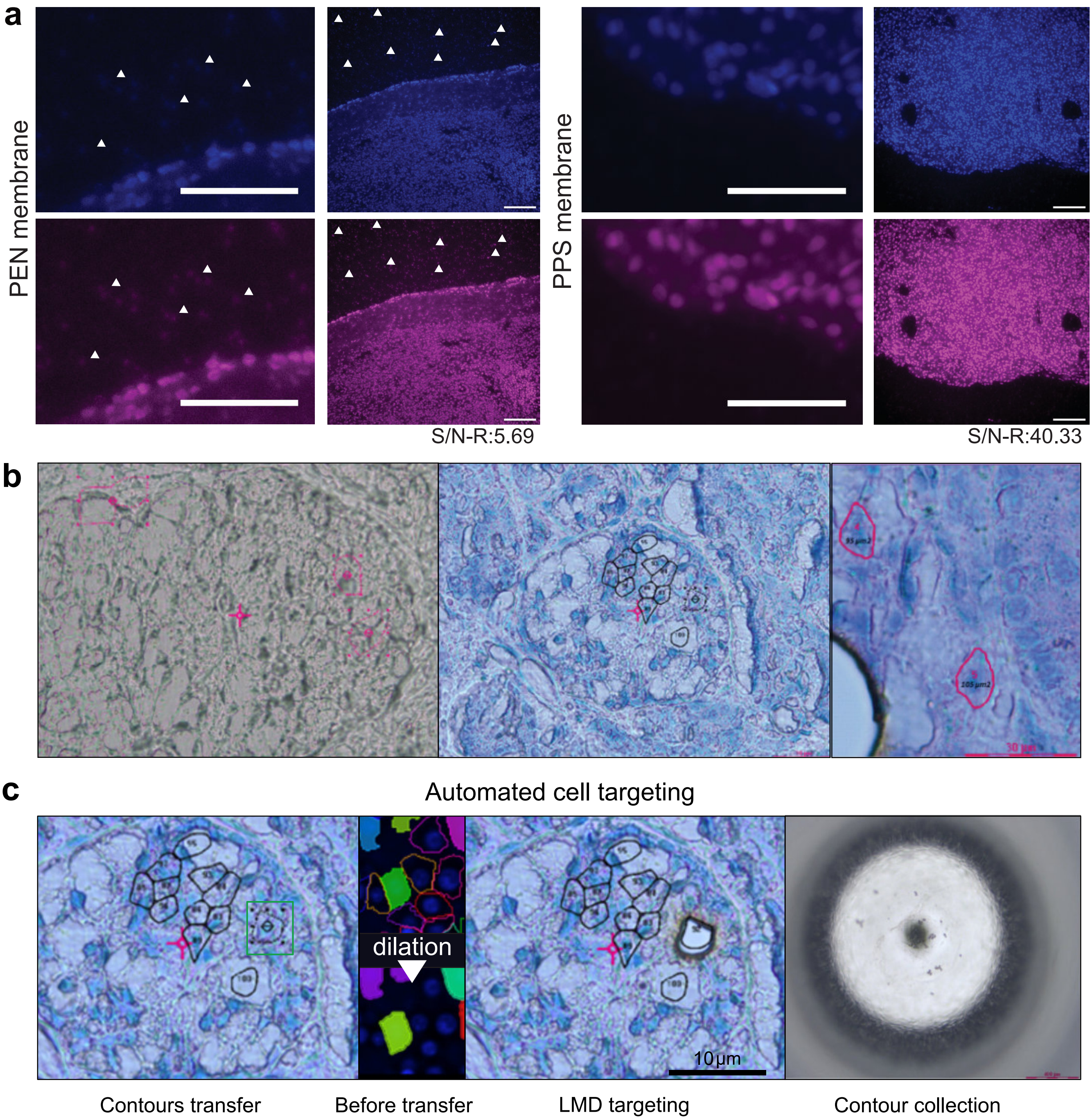

Supplementary Figure 4: Spatial organization and compartmentalization of pancreatic endocrine cell types.

a) Representative images comparing image quality between untreated PEN and PPS membranes, shown as zoomed views (left) and overviews (right). Top panels show the Hoechst nuclear stain; bottom panels show the same stain with a magenta lookup table for better contrast. White arrows indicate granules from the nuclear stain on PEN membranes. Brightness and contrast were adjusted linearly and identically for all panels. Scale bars, 50 µm (left) and 100 µm (right). b) Example of brightfield images with imported contours before polychromatic Toluidine stain (left) and after (right). c) Illustrative images of key steps in the developed pipeline for automated cell targeting by LMD where (1) contours for each identified subpopulation are imported into the LMD software, (2) contours are targeted by LMD, and (3) excised contours are collected into a 384-well plate and visually quality controlled.

Supplementary Figure 4

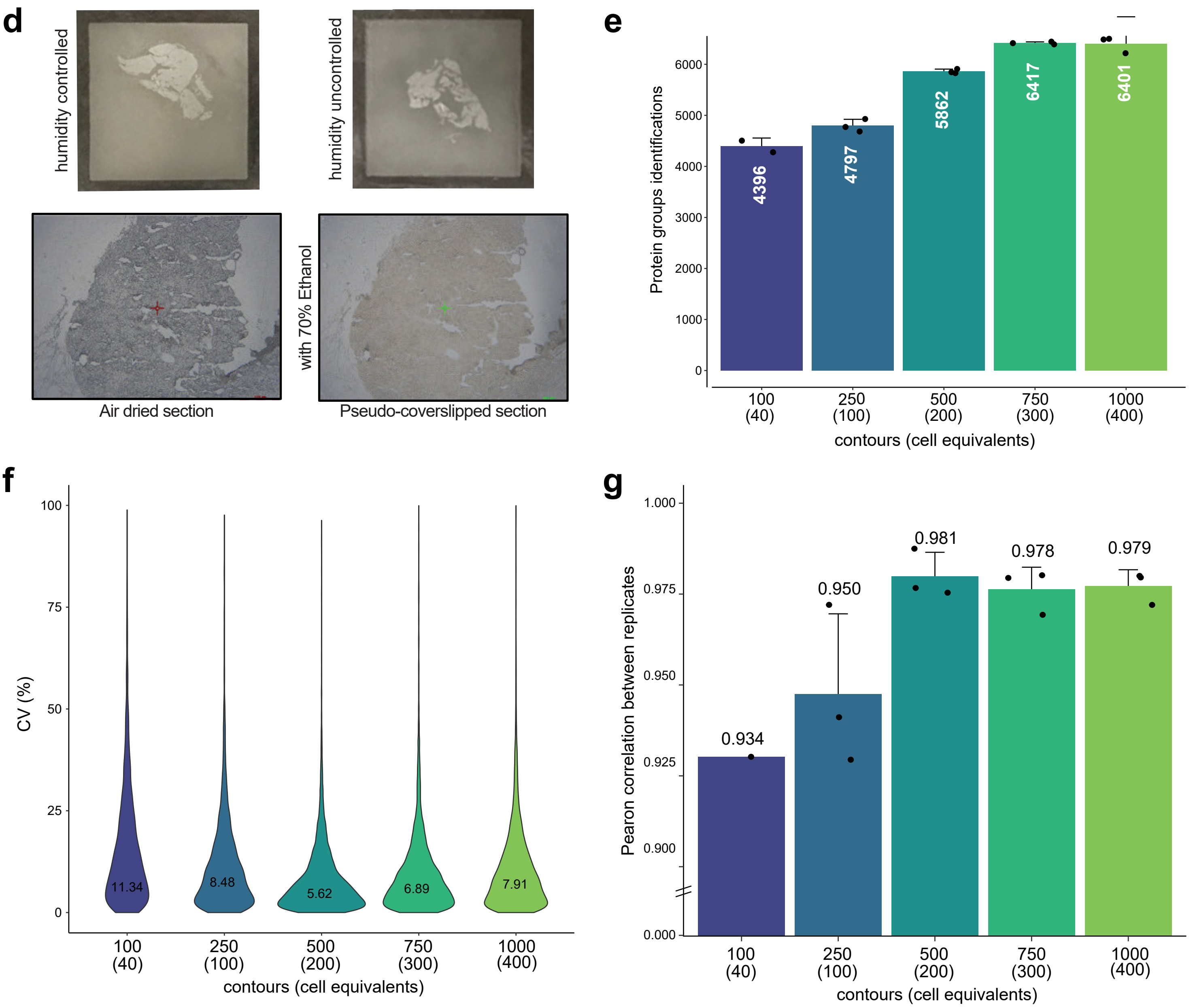

Supplementary Figure 4: Spatial organization and compartmentalization of pancreatic endocrine cell types.

d) Example images of coverslip PPS membranes with pancreas tissue section with controlled humidity during storage before and during tissue processing versus non-controlled humidity (left). Dry section before cutting versus pseudo coverslipped tissue with 10ul of 70% Ethanol added (right). e) Protein group identification of contour dilution series. Bar plot represents the mean of replicates, while the error bars are one standard error of the mean. Workflow replicates, n=3.Source data are provided as a Source Data file. f) Coefficient of variations (CVs) between replicates of contour dilution series. Workflow replicates, n=2. Source data are provided as a Source Data file. g) Pearson correlation between replicates of the contour dilution series. Workflow replicates, n=3. Source data are provided as a Source Data file.

Supplementary Figure 5

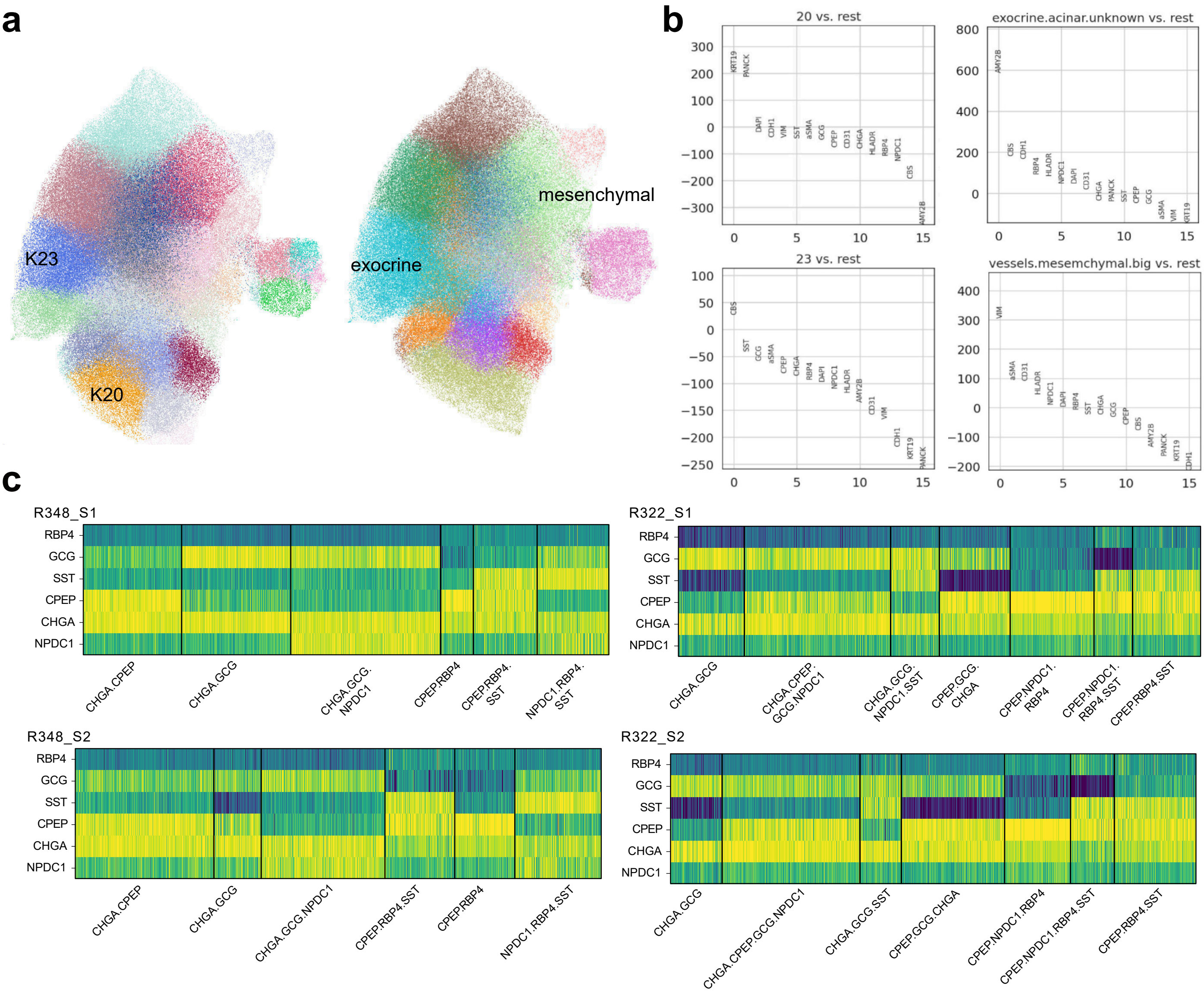

Supplementary Figure 5: Proteomic and spatial analysis reveal endocrine cell state heterogeneity.

a) Example of a UMAP representation of all cells in one sample colored by K-means clustering with clusters 20 and 23 labeled (left). The UMAP is colored by cluster after cluster refinement in PIPΣX with the annotated cell type labeled (right). b) Corresponding plots showcasing ranked protein expression for clusters 20 and 23 before cluster refinement (left) and after cluster refinement (right). c) Heatmaps for each donor and section displaying expression profiles of annotated islet subpopulations as identified by K-means clustering of multiplex image data (log1p-transformed normalized intensity). A multiplexed dataset was used for the comparison.

Supplementary Figure 5

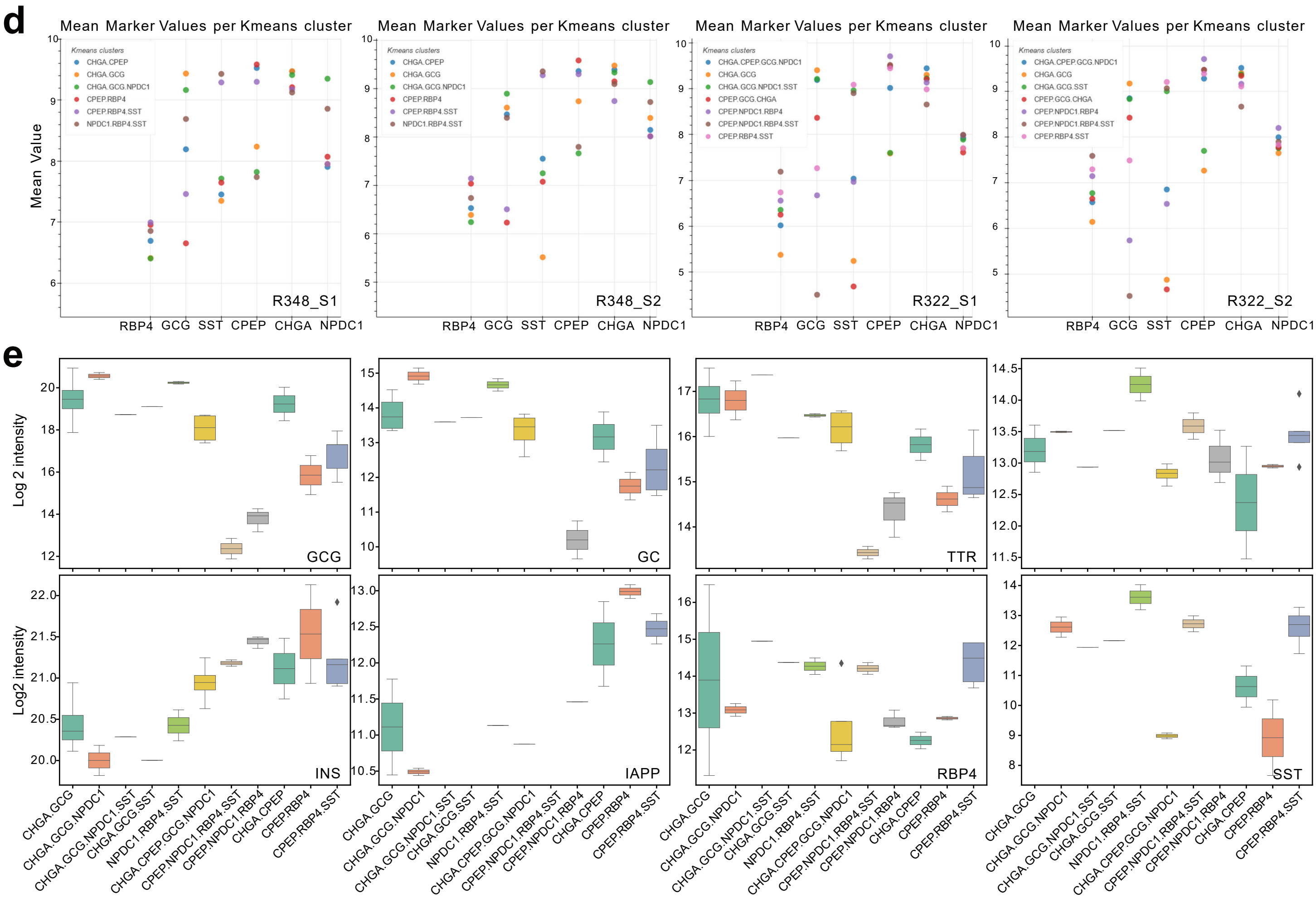

Supplementary Figure 5: Proteomic and spatial analysis reveal endocrine cell state heterogeneity.

d) Scatter plots for each donor and section showing the mean marker value (log1p-transformed normalized intensity) for each annotated cluster identified by K-means clustering across all islet cell markers in the antibody panel. A multiplexed dataset was used for the comparison. e) Mass spectrometry-based proteomic analysis of pancreatic endocrine cell subpopulations. The bar plot displays log2-transformed median expression levels of key endocrine markers (INS, GCG, RBP4, TTR, GC, CHGA, IAPP, SST, INSM1, NPDC1) across distinct cell subgroups. Error bars indicate standard deviations.

Supplementary Figure 6

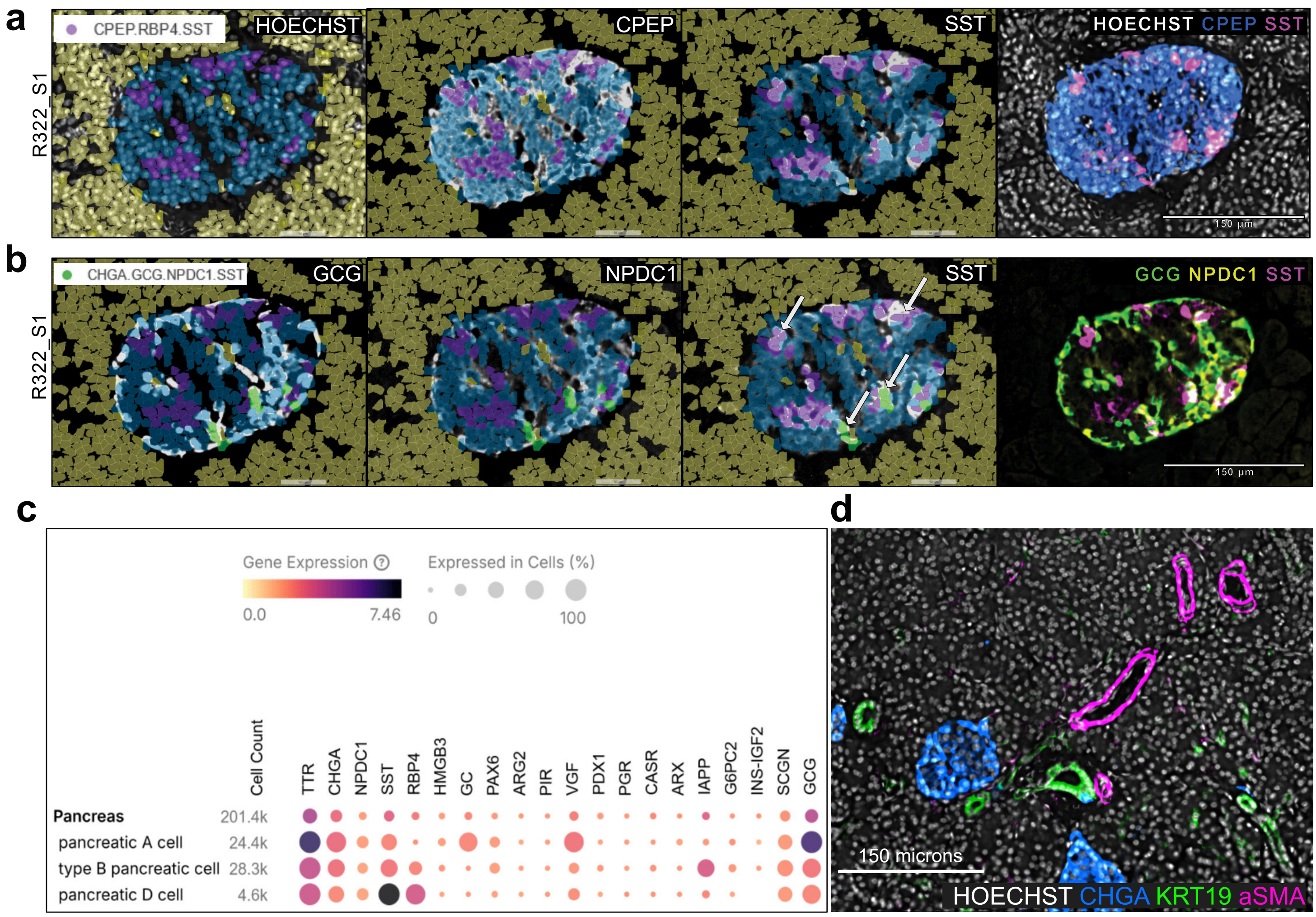

Supplementary Figure 6: Spatial organization and compartmentalization of pancreatic endocrine cell types.

a) Illustrative example of a CPEP-, RBP4-, and SST-coexpressing cell population. Cell segmentation masks are overlaid, with the coexpressing population shown in purple, endocrine cells in blue, and other cell types in yellow. b) Illustrative example of a GCG-, NPDC1-, and SST-coexpressing cell population within the same islet. Cell segmentation masks are overlaid, with the coexpressing polyhormonal population shown in green, endocrine cells in blue, and other cell types in yellow. c) Dot-plot highlighting differentially expressed genes for pancreatic α-cells, β-cells, and δ-cells from a publicly available single-cell annotated transcriptomic dataset (Tabula Sapiens). d) Representative high-plex image showing the observed spatially distinct organization of endocrine, ductal, and vascular compartments.

Supplementary Figure 6

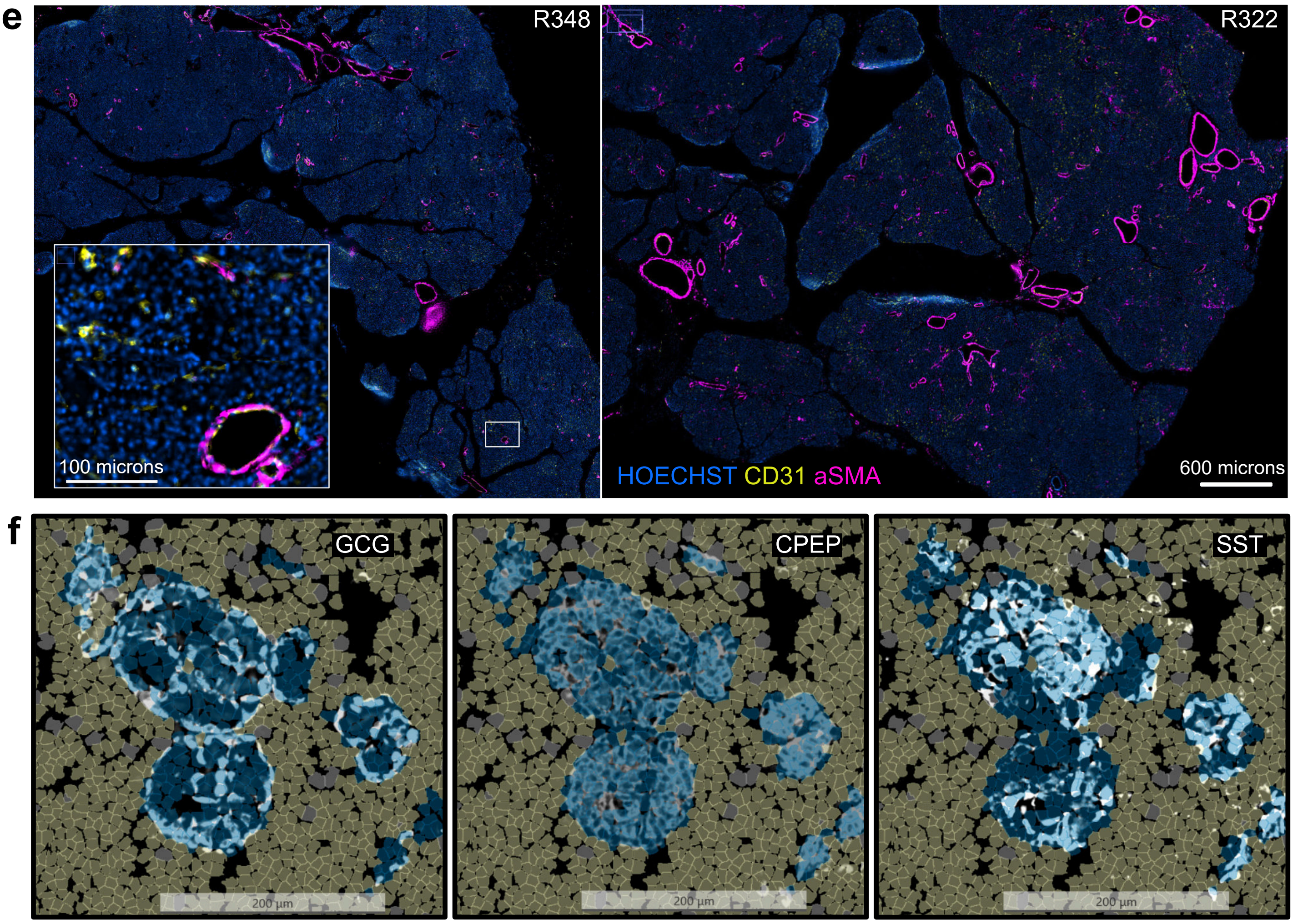

Supplementary Figure 6: Spatial organization and compartmentalization of pancreatic endocrine cell types.

e) Example multiplex images visualizing vascular markers, showcasing observed expression differences between vessels of different calibers. f) Illustrative example images of observed compartmentalization of β-cells (CPEP) and α-cells (GCG). Cell segmentation masks overlaid the images showing endocrine cells (blue) and other cell types (yellow).

Supplementary Figure 7

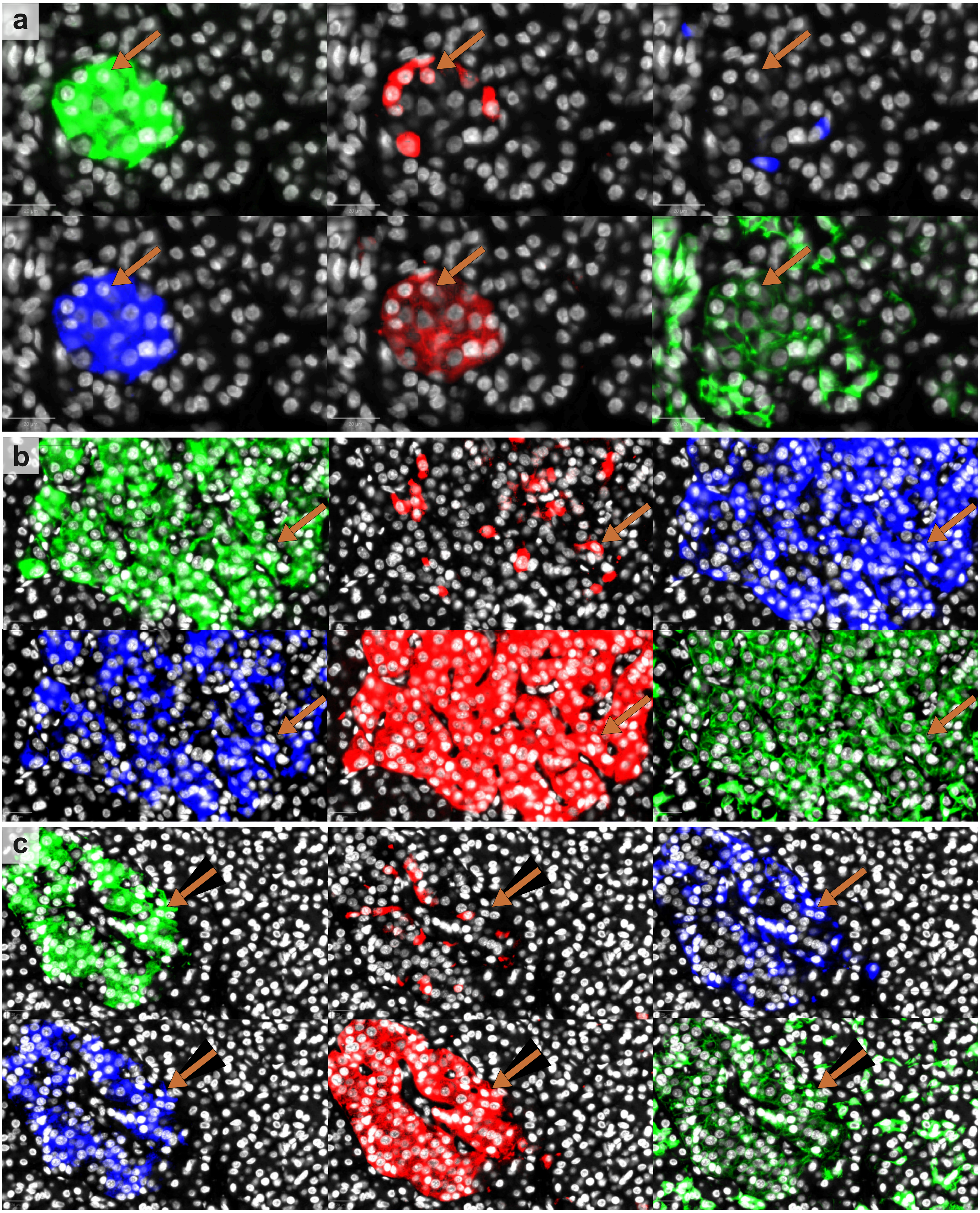

Supplementary Figure 7: CODEX imaging from independent HPAP donors (https://hpap.pmacs.upenn.edu/) confirms polyhormonal endocrine cell populations in healthy human pancreatic islets.

a) Representative CODEX images (FFPE) from donor HPAP-014 showing a small islet. Orange arrows indicate a single cell co-expressing C-peptide (CPEP, β-cell), somatostatin (SST, δ-cell), proinsulin (ProINS), and synaptophysin (SYP, pan-endocrine), but not glucagon (GCG, α-cell). ATP1A1 (Na/K-ATPase) marks cell membranes and allows assessment of signal bleedthrough. The consistent arrow position across channels confirms co-expression of markers from distinct endocrine lineages within a single cell. b) Donor HPAP-035: a single cell co-expressing SST, GCG, and SYP but not CPEP or ProINS, consistent with an α/δ polyhormonal identity. c) Donor HPAP-054: a single cell co-expressing CPEP, GCG, and SYP but not ProINS or SST, consistent with an α/β polyhormonal identity. Co-expression of markers from distinct endocrine lineages across three donors processed by independent laboratories indicates that polyhormonal cells occur in healthy human islets and are not specific to the antibody panel or tissue processing used here.

Supplementary Figure 8

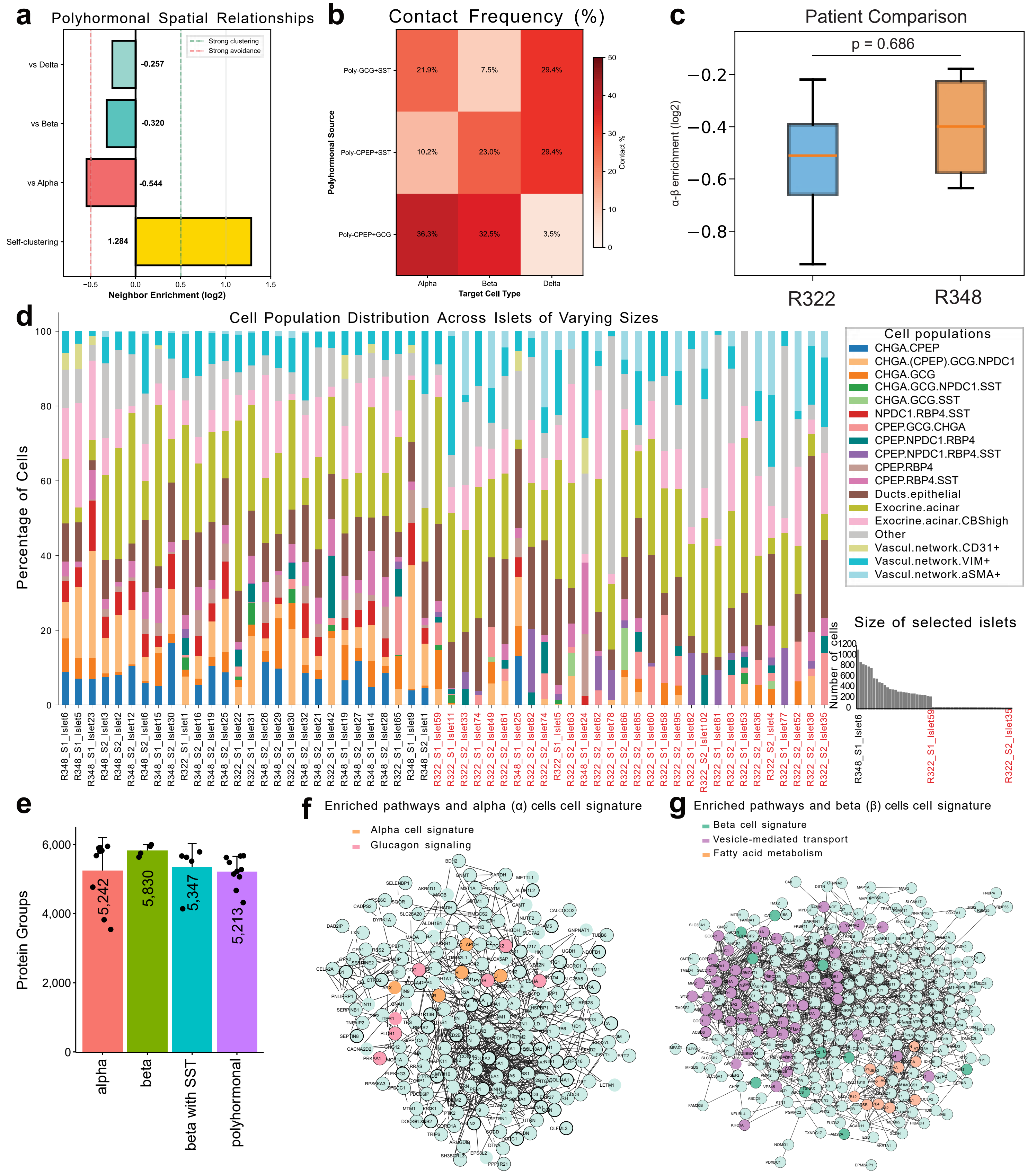

Supplementary Figure 8: Spatial organization and proteomic characterization of polyhormonal and endocrine cell populations across islets.

a) Polyhormonal neighbor enrichment analysis. Bar plot shows spatial enrichment scores (log_2) for polyhormonal cells relative to pure endocrine cell types. Polyhormonal cells exhibit strong self-clustering (enrichment = +1.284) and spatial avoidance of pure α-cells (−0.544), β-cells (−0.320), and δ-cells (−0.257). Vertical dashed lines indicate thresholds for strong clustering (gold) and strong avoidance (red). Enrichment calculated as log_2((observed + 1)/(expected + 1)); permutation test with 1,000 iterations, Benjamini-Hochberg FDR correction. Source data are provided as a Source Data file. b) Contact frequency matrix for polyhormonal populations. Heatmap shows the percentage of polyhormonal source cells in direct contact with target cell types (α, β, δ). Poly-GCG+SST and Poly-CPEP+SST cells show highest contact with δ-cells (29.4% each), while Poly-CPEP+GCG cells preferentially contact α-cells (36.3%). Values represent the percentage of source cells with at least one target-cell neighbor; two-sided binomial test against 10% random-contact expectation, Benjamini-Hochberg FDR correction. Source data are provided as a Source Data file. c) Donor comparison of α-β spatial segregation. Box plots compare α-β enrichment scores between donors R322 (n ≈ 90 islets) and R348 (n ≈ 55 islets); no significant difference (Mann-Whitney U test, two-sided, $p = 0.686$). Box: interquartile range; center line: median; whiskers: 1.5× IQR. Source data are provided as a Source Data file. d) Proportions of annotated islet cell populations and immediately surrounding vasculature across islets of varying size, ordered by islet size (inset, number of cells per islet). Smaller islets shown in red text on the x-axis are those highlighted in the size inset. Source data are provided as a Source Data file. e) Number of protein groups identified by MS for each isolated endocrine cell type, pooled across both donors (see also Fig. 4a). f) Protein-protein interaction network of the α-cell proteomic signature (STRING), coloured by enriched pathway (alpha cell signature, orange; glucagon signalling, pink). g) Protein-protein interaction network of the β-cell proteomic signature (STRING), coloured by enriched pathway (beta cell signature, green; vesicle-mediated transport, purple; fatty acid metabolism, orange). Enrichment for f and g assessed by Fisher exact test with Benjamini-Hochberg correction (FDR $q < 0.02$).

Supplementary Figure 9

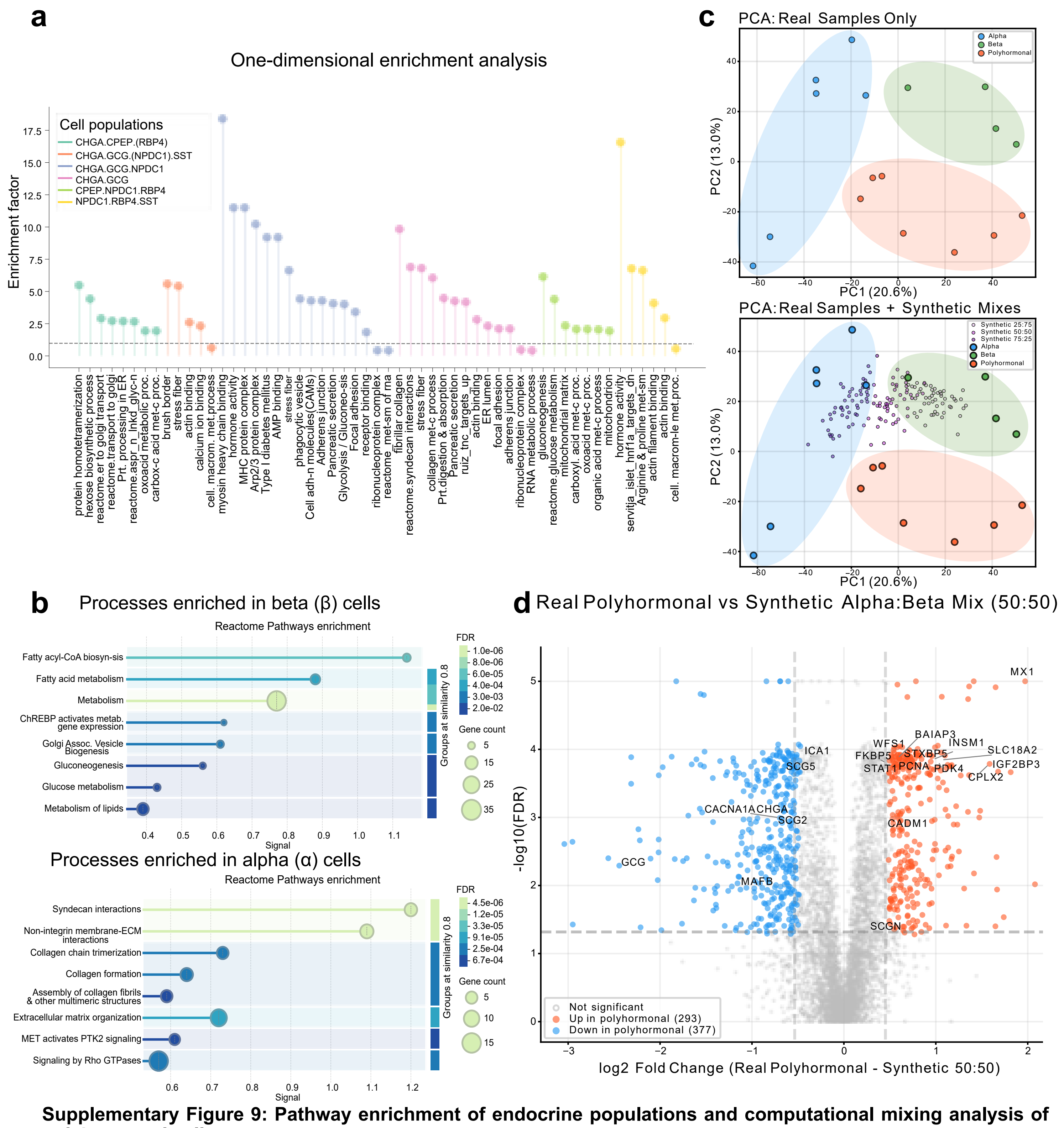

Supplementary Figure 9: Pathway enrichment of endocrine populations and computational mixing analysis of polyhormonal cells.

a) One-dimensional enrichment analysis of cellular processes associated with enriched proteins for each endocrine cell population (FDR < 0.05). Cell populations are indicated by colour; the dashed line indicates an enrichment factor of 1. b) Reactome pathway enrichment of processes enriched in β-cells (top) and α-cells (bottom). Point size indicates gene count and colour indicates FDR; pathways grouped at similarity 0.8, FDR < 0.05. c) Principal component analysis (PCA) of real endocrine cell proteomic profiles and synthetic alpha-beta mixes. Top, PCA of real samples only: alpha cells (blue, n = 6), beta cells (green, n = 4), polyhormonal cells (orange, n = 7). Bottom, the same PCA space with 50 synthetic profiles per mixing ratio projected (25:75, 50:50, 75:25 alpha:beta). Synthetic mixes fall along the alpha-beta interpolation axis, while real polyhormonal cells occupy a distinct region of proteome space not recapitulated by linear combination of classical cell-type profiles. Missing values were imputed by K-nearest neighbours (k = 5) prior to PCA. Variance explained is indicated on the axes. Source data are provided as a Source Data file. d) Volcano plot comparing real polyhormonal cells against synthetic 50:50 alpha-beta mixes. x-axis, $\log_2$ fold change (real polyhormonal minus synthetic mix); y-axis, $-\log_{10}$(FDR) from Mann-Whitney U tests with Benjamini-Hochberg correction. Orange, significantly upregulated in real polyhormonal cells (FDR < 0.05, $\log_2$FC > 0.5); blue, significantly downregulated (FDR < 0.05, $\log_2$FC < −0.5); grey, not significant. Labelled markers include endocrine differentiation factors (INSM1, IGF2BP3), a proliferation marker (PCNA), secretory vesicle components (SLC18A2, CPLX2, BAIAP3), and stress-response proteins (STAT1, MX1). Classical endocrine hormones (GCG, SST, CHGA) are downregulated relative to the synthetic mix. Across all three mixing ratios, 1,427 proteins were consistently differentially expressed (FDR < 0.05), consistent with polyhormonal signatures representing a distinct cell state rather than adjacent-cell contamination. Bootstrap replicates, n = 1,000 per ratio. Source data are provided as a Source Data file.

Supplementary Figure 10

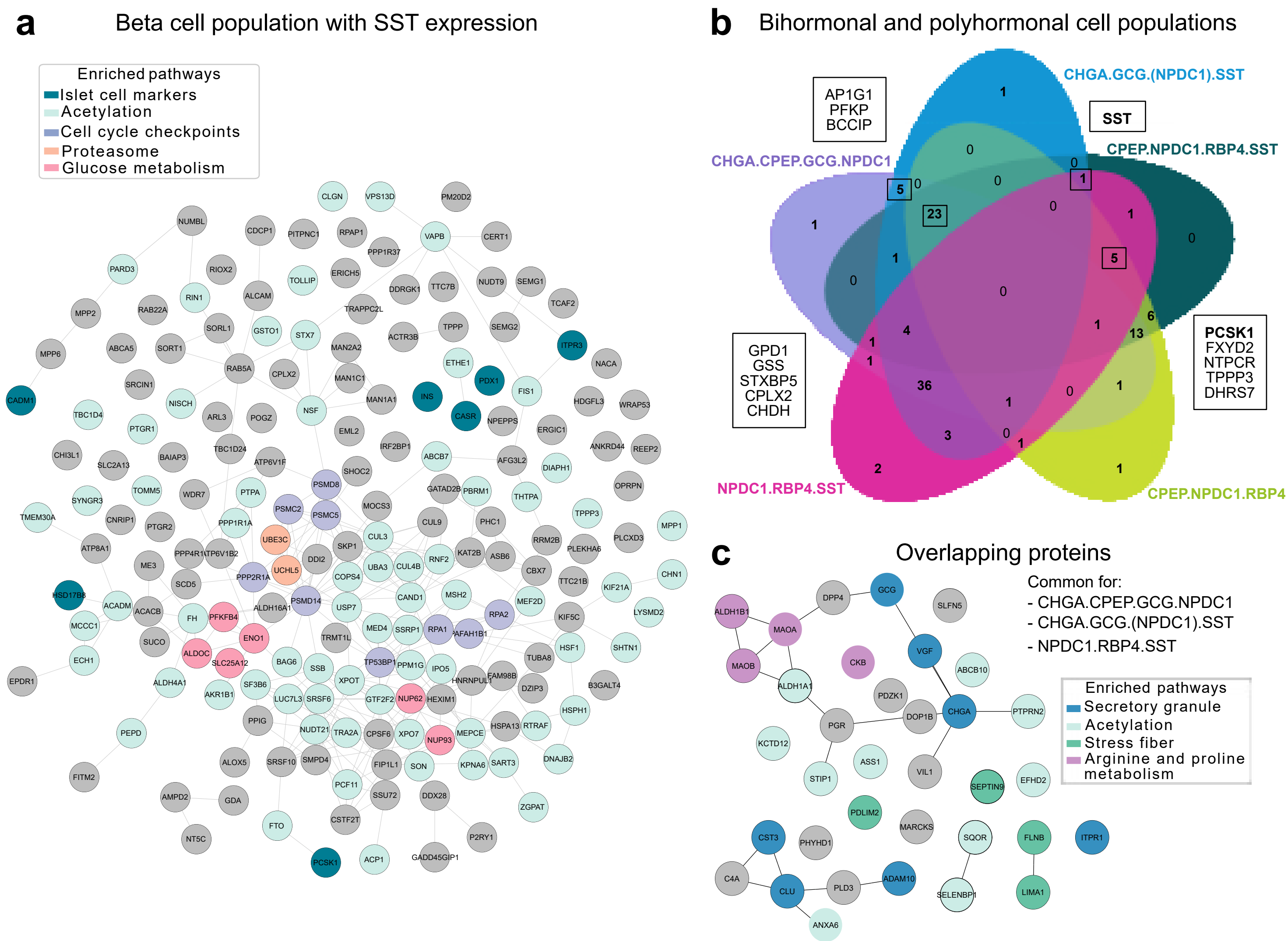

Supplementary Figure 10: Proteomic characterization of bihormonal and polyhormonal cell populations and protein–transcript concordance per cell type.

a) Protein-protein interaction network of the beta cell population with SST expression (STRING), coloured by enriched pathway: islet cell markers (dark teal), acetylation (light teal), cell cycle checkpoints (purple), proteasome (orange), glucose metabolism (pink). b) Venn diagram of proteins detected by MS across the bihormonal and polyhormonal cell populations. Numbers indicate proteins unique to or shared between populations; boxed labels indicate representative proteins, including the 23 proteins common to CHGA.CPEP.GCG.NPDC1, CHGA.GCG.(NPDC1).SST and NPDC1.RBP4.SST. Differential abundance by one-way ANOVA with post-hoc Tukey's HSD (two-sided, Benjamini-Hochberg FDR < 0.05). c) Protein-protein interaction network of the 23 common proteins (STRING), coloured by enriched pathway: secretory granule (blue), acetylation (light teal), stress fiber (green), arginine and proline metabolism (pink). Network enrichment in a and c assessed by Fisher exact test with Benjamini-Hochberg correction (FDR q < 0.02).

Supplementary Figure 10

d

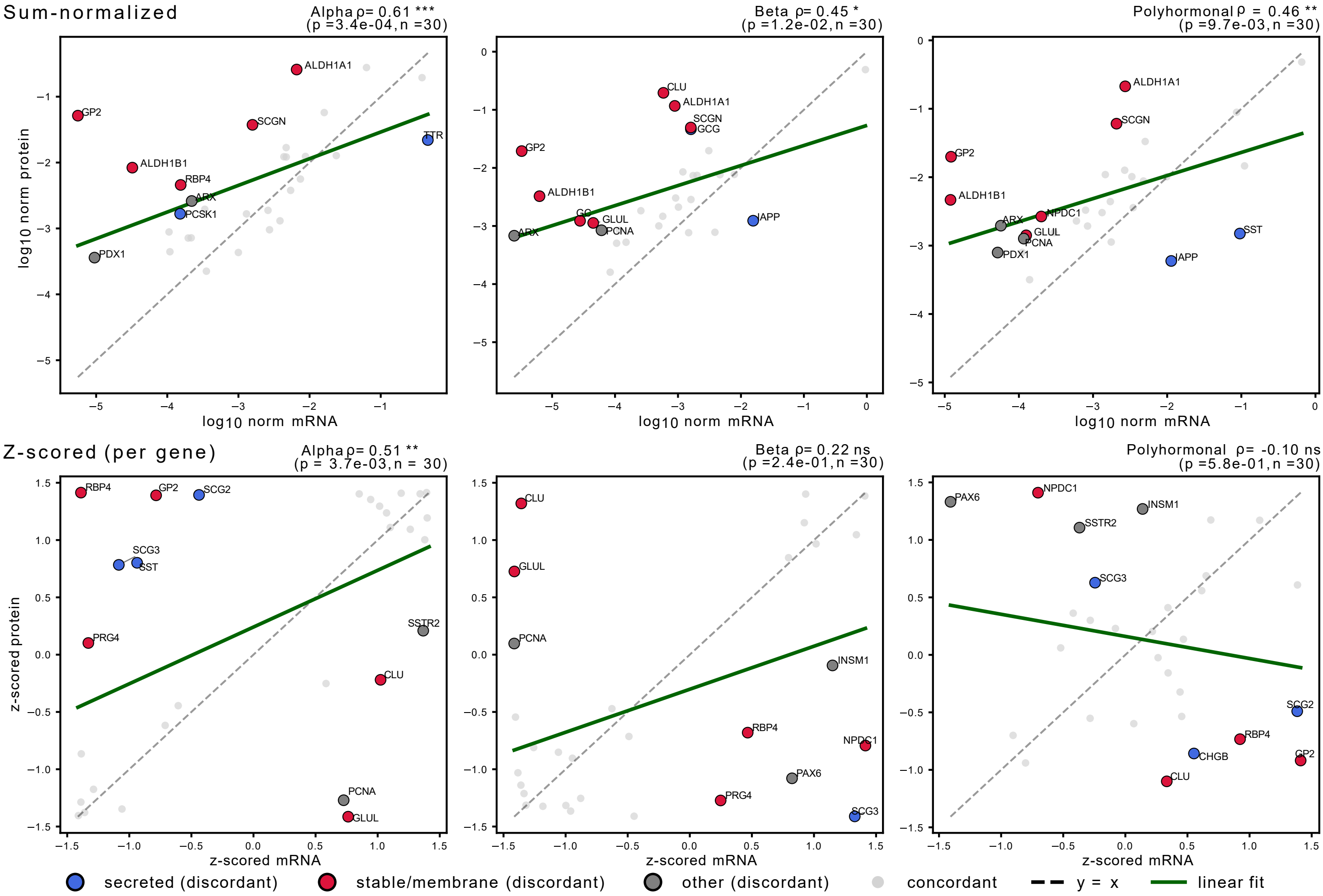

Supplementary Figure 10: Proteomic characterization of bihormonal and polyhormonal cell populations and protein–transcript concordance per cell type.

d) Correlation between protein and transcript abundance per endocrine cell type across the shared feature set (n = 30 genes). Top row, values normalised internally over the sum of the shared set and $\log_{10}$-transformed (alpha, Spearman ρ = 0.61, p = 3.4 × 10^{-4}; beta, ρ = 0.45, p = 1.2 × 10^{-2}; polyhormonal, ρ = 0.46, p = 9.7 × 10^{-3}). Bottom row, the same comparison after per-gene z-scoring across cell types, which removes the abundance axis and isolates cell-type-specific expression pattern (alpha, ρ = 0.51, p = 3.7 × 10^{-3}; beta, ρ = 0.22, p = 0.24; polyhormonal, ρ = −0.10, p = 0.58). All correlations are two-sided Spearman rank correlations, n = 30 genes per panel. Points coloured by concordance and, where discordant, by protein class: secreted (blue), stable/membrane (red), other (grey); concordant features in light grey. Discordance defined as an absolute inter-modality difference > 1 $\log_{10}$ or z-score unit. Green line, linear fit. Dashed line, identity relationship, shown for reference only as the two modalities are normalised independently. Source data are provided as a Source Data file.

Supplementary Information

Supplementary Table 1. Donor and sample characteristics. Demographic and clinical characteristics of the two organ donors (R322 and R348) and the tissue sections analyzed in this study, including sex, age range, and diabetes/pancreatitis history.

** Donor information retrieved from HumanIslets.com, a community resource in support of research in islet biology, transplantation, and diabetes* https://www.humanislets.com/#/donor

ID	Sex	Diagnosis	Age decade	BMI	Adjusted diabetes status	HbA1c (%)	HLA A2
**R348**	Female	None	40-49	16.4	None	5.7	**Positive**
**R322**	Female	None	40-49	23.2	None	4.9	**Negative**

Supplementary Table 2. CODEX imaging cycle and channel configuration. Acquisition layout for the multiplexed CODEX run, listing the antigen imaged in each fluorophore channel (DAPI, Atto550, AF647, AF750) and the corresponding exposure time (ms) for each imaging cycle. Blank cycles at the start and end serve as background/autofluorescence controls.

Cycle	DAPI	DAPI exposure [ms]	Atto550 antigen	Atto550 exposure [ms]	AF647 antigen	AF647 exposure [ms]	AF750 antigen	AF750 antigen exposure [ms]
1	DAPI	40	Blank	100	Blank	200	Blank	750
2	DAPI	40	CDH1	750	RBP4	750	VIM	750
3	DAPI	40	GCG	100	AMY2B	200	CD31	750
4	DAPI	40	SST	100	HLA-DR	750	SMA	750
5	DAPI	40	CPEP	350	CHGA	200	KRT19	750
6	DAPI	40	CBS	750	NPDC1	750	PANCK	750
7	DAPI	40	Blank	100	Blank	200	Blank	750

Supplementary Table 3. Laser microdissection settings. Leica LMD7 laser parameters used for single-cell contour excision, including power, aperture, and pulse frequency.

Parameter	Setting
Power	8-15
Aperture	7-10
Speed	2
Middlepulse count	3
Final Pulse	4
Head current	89
Pulse frequency [Hz]	300-800

Supplementary Table 4. Endocrine populations categorical annotations

Name	Group	Cell Type
R322_section1_E6_CHGA.GCG_cl2	CHGA.GCG	alpha
R322_section2_C3_CHGA.GCG_cl1	CHGA.GCG	alpha
R348_section1_M6_CHGA.GCG_cl8	CHGA.GCG	alpha
R348_section2_K6_CHGA.GCG_cl3	CHGA.GCG	alpha
R322_section1_E8_CHGA.CPEP.GCG.NPDC1_cl9	CHGA.(CPEP).GCG.NPDC1	alpha with CPEP
R322_section2_C8_CHGA.CPEP.GCG.NPDC1_cl8	CHGA.(CPEP).GCG.NPDC1	alpha with CPEP
R322_section2_C9_CHGA.CPEP.GCG.NPDC1small_cl9	CHGA.(CPEP).GCG.NPDC1	alpha with CPEP
R348_section1_M8_CHGA.GCG.NPDC1_cl10	CHGA.(CPEP).GCG.NPDC1	alpha with CPEP
R348_section2_K8_CHGA.GCG.NPDC1_cl9	CHGA.(CPEP).GCG.NPDC1	alpha with CPEP
R322_section1_E18_CHGA.GCG.NPDC1.SST_cl3	CHGA.GCG.(NPDC1).SST	alpha with SST
R322_section2_C18_CHGA.GCG.SST_cl2	CHGA.GCG.(NPDC1).SST	alpha with SST
R348_section1_M3_CHGA.CPEP_cl6	CHGA.CPEP	beta
R348_section2_K3_CHGA.CPEP_cl5	CHGA.CPEP	beta
R348_section1_M11_CPEP.RBP4_cl2	CPEP.RBP4	beta
R348_section2_K11_CPEP.RBP4_cl2	CPEP.RBP4	beta
R322_section1_E11_CPEP.RBP4.SST_cl6	CPEP.RBP4.SST	beta with SST
R322_section2_C11_CPEP.RBP4.SST_cl7	CPEP.RBP4.SST	beta with SST
R322_section2_C12_CPEP.RBP4.SSTsmall_cl6	CPEP.RBP4.SST	beta with SST
R348_section1_M12_CPEP.RBP4.SST_cl3	CPEP.RBP4.SST	beta with SST
R348_section2_K15_CPEP.RBP4.SST_cl4	CPEP.RBP4.SST	beta with SST
R322_section2_C14_CPEP.GCG.CHGA_cl10	CPEP.GCG.CHGA	Polyhormonal
R322_section1_E3_CPEP.NPDC1.RBP4_cl4	CPEP.NPDC1.RBP4	Polyhormonal
R322_section2_C4_CPEP.NPDC1.RBP4small_cl5	CPEP.NPDC1.RBP4	Polyhormonal
R322_section2_C6_CPEP.NPDC1.RBP4_cl4	CPEP.NPDC1.RBP4	Polyhormonal
R322_section1_E17_CPEP.NPDC1.RBP4.SST_cl1	CPEP.NPDC1.RBP4.SST	Polyhormonal
R322_section2_C17_CPEP.NPDC1.RBP4.SST_cl3	CPEP.NPDC1.RBP4.SST	Polyhormonal
R348_section1_M17_NPDC1.RBP4.SST_cl4	NPDC1.RBP4.SST	Polyhormonal
R348_section2_K17_NPDC1.RBP4.SST_cl7	NPDC1.RBP4.SST	Polyhormonal
