# Supplementary material for: Multiplexed deep visual proteomics resolves spatial heterogeneity and rare endocrine states in human pancreatic islets
Source: Nat Commun. 2026 Sep 25;17:11138. doi: 10.1038/s41467-026-77890-6 (PMC13710848; doi:10.1038/s41467-026-77890-6)
Supplement: Supplementary file 3 [file 41467_2026_77890_MOESM2_ESM.pdf]

Description of Additional Supplementary Files

File Name: Supplementary Data 1
Description: Differentially abundant proteins across endocrine cell populations. Proteins significantly enriched between alpha, beta, and beta-with-SST populations, with log2 fold changes and adjusted p values (one-way ANOVA followed by post-hoc Tukey's HSD, two-sided, Benjamini-Hochberg FDR < 0.05).

File Name: Supplementary Data 2
Description: Antibody panel. Full list of antibodies used for high-plex CODEX imaging and validation, including target, supplier, catalogue number, clone, lot number, host species, formulation, working concentration, and assigned CODEX barcode.

File Name: Supplementary Data 3
Description: PIPΣX image quantification matrix with per-cell marker intensities, assigned cell-type annotations, and a flag indicating cells selected for laser microdissection targeting, across all donors and tissue sections.

File Name: Supplementary Data 4
Description: Mass spectrometry protein-group quantification matrix (DIA-NN output) used for downstream proteomic analysis, with protein groups in rows and mass spectrometry samples in columns. Matrix after log-transformation and potential contaminants filtration.

File Name: Supplementary Data 5
Description: Protein and transcript abundance for the shared feature set used in the cross-omics correlation (Fig. 5f). Per-gene log10-transformed abundance, normalized internally over the sum

of the shared feature set within each cell type, for the 30 genes measured in both the mxDVP proteomic data and the Yoon et al. single-cell RNA-seq data (GSE196715). Discordance was defined as an absolute inter-modality difference > 1 log10 unit. The correlation in Fig. 5f is pooled across alpha, beta and polyhormonal cell types (n = 90); delta and PP cells were excluded.

File Name: Supplementary Movie 1

Description: Real-time recording of automated single-cell laser microdissection. The video shows the laser microdissection of individual annotated single-cell contours through the Leica LMD7 63× objective. Contours identified and exported by PIPΣX are imported into the LMD software, targeted by the laser, and excised, demonstrating the precision of single-cell isolation and the integrity of neighboring cells. Duration: 16 seconds, 1× speed.
