# Supplementary material for: Multiplexed deep visual proteomics resolves spatial heterogeneity and rare endocrine states in human pancreatic islets
Source: Nat Commun. 2026 Sep 25;17:11138. doi: 10.1038/s41467-026-77890-6 (PMC13710848; doi:10.1038/s41467-026-77890-6)
Supplement: Supplementary file 4 [file 41467_2026_77890_MOESM9_ESM.pdf]

nature portfolio

Corresponding author(s): Emma Lundberg

Last updated by author(s): Jul 10, 2026

Reporting Summary

Nature Portfolio wishes to improve the reproducibility of the work that we publish. This form provides structure for consistency and transparency in reporting. For further information on Nature Portfolio policies, see our Editorial Policies and the Editorial Policy Checklist.

Statistics

For all statistical analyses, confirm that the following items are present in the figure legend, table legend, main text, or Methods section.

n/a	Confirmed	
☐	☒	The exact sample size (*n*) for each experimental group/condition, given as a discrete number and unit of measurement
☐	☒	A statement on whether measurements were taken from distinct samples or whether the same sample was measured repeatedly
☐	☒	The statistical test(s) used AND whether they are one- or two-sided<br>*Only common tests should be described solely by name; describe more complex techniques in the Methods section.*
☒	☐	A description of all covariates tested
☐	☒	A description of any assumptions or corrections, such as tests of normality and adjustment for multiple comparisons
☐	☒	A full description of the statistical parameters including central tendency (e.g. means) or other basic estimates (e.g. regression coefficient) AND variation (e.g. standard deviation) or associated estimates of uncertainty (e.g. confidence intervals)
☐	☒	For null hypothesis testing, the test statistic (e.g. *F*, *t*, *r*) with confidence intervals, effect sizes, degrees of freedom and *P* value noted<br>*Give P values as exact values whenever suitable.*
☒	☐	For Bayesian analysis, information on the choice of priors and Markov chain Monte Carlo settings
☐	☒	For hierarchical and complex designs, identification of the appropriate level for tests and full reporting of outcomes
☐	☒	Estimates of effect sizes (e.g. Cohen's *d*, Pearson's *r*), indicating how they were calculated

Our web collection on statistics for biologists contains articles on many of the points above.

Software and code

Policy information about availability of computer code

Data collection	CODEX Instrument Manager (CIM, v. 1.30.0.12) for automated fluidic cycles and multiplexed image acquisition. Leica Application Suite X (LAS X, v. 3.7.5.24914) for image acquisition on Leica DMI8 widefield microscope. CODEX Processor (v. 1.7.0.6) for image processing, stitching, background subtraction, and deconvolution. Leica LMD software (v. 8.3.745) for laser microdissection. MSConvert (Proteowizard v. 3.0.23216) for raw file conversion.
Data analysis	PIPΣX (v. 1.0) - in-house developed open-source Python software for image preprocessing, segmentation, quantification, clustering, and annotation (https://github.com/CellProfiling/pipex). StarDist for nuclear segmentation. BIAS (v. 1.1.0, Single-Cell Technologies Ltd.) for contour processing. TissUUmaps for visualization, and image exploration. DIA-NN (v. 1.8.1) for mass spectrometry raw data analysis. Python (v3.9.4), R (v.4.2.2), Perseus (v. 2.0.10) for statistical analysis. Python (v. 3.9.4) with seaborn for visualization. Cytoscape (v. 3.8 and v. 3.10.3) for protein interaction networks. ImageJ (v. 1.53) for image export. py_diAID for dia-PASEF method optimization.

For manuscripts utilizing custom algorithms or software that are central to the research but not yet described in published literature, software must be made available to editors and reviewers. We strongly encourage code deposition in a community repository (e.g. GitHub). See the Nature Portfolio guidelines for submitting code & software for further information.

Data

Policy information about availability of data

All manuscripts must include a data availability statement. This statement should provide the following information, where applicable:

- Accession codes, unique identifiers, or web links for publicly available datasets
- A description of any restrictions on data availability
- For clinical datasets or third party data, please ensure that the statement adheres to our policy

All supplementary tables and supplementary data associated with this study are available at Zenodo: https://doi.org/10.5281/zenodo.21726555. The high-plex image data is available in EBI Image Archive BioImages (https://www.ebi.ac.uk/biostudies/BioImages/studies) with the accession number S-BIAD1853 https://doi.org/10.6019/S-BIAD1853. All mass spectrometry raw files and proteomics data analyzed in this study have been deposited to the ProteomeXchange Consortium via the PRIDE partner repository, project accession PXD062166 (http://www.ebi.ac.uk/pride). The deposited dataset additionally includes mass spectrometry data from a third donor that was part of the combined database used for peptide and protein identification; all analyses reported in this study are based on the two donors (R322 and R348) described in Supplementary Table 1. All code for image preprocessing, segmentation, postprocessing and analysis associated with current submission is provided as a part of initial PIPΣX release (version 1.0) 92. And the current version of the software is available at https://github.com/CellProfiling/pipex under the MIT license. The special PIPΣX-BIAS integration script is available at https://github.com/CellProfiling/pipex/tree/main/extra under the MIT license.

Research involving human participants, their data, or biological material

Policy information about studies with human participants or human data. See also policy information about sex, gender (identity/presentation), and sexual orientation and race, ethnicity and racism.

Reporting on sex and gender	Both donors were female. Sex was considered in study design as donors were matched for sex to reduce biological variability. Sex was determined based on donor records provided by the Alberta Diabetes Institute IsletCore. Individual-level sex data is provided in Supplementary Table 1.
Reporting on race, ethnicity, or other socially relevant groupings	Socially constructed categorization was not taken into consideration. Patient material was provided by the Alberta Diabetes Institute (ADI) IsletCore, Canada (http://www.bcell.org/adi-isletcore.html).
Population characteristics	Two female donors of similar age with no previous history of diabetes mellitus or pancreatitis (donor identifiers: R348 and R322). Low BMI and relatively healthy insulin secretion. Full donor characteristics are described in Supplementary Table 1 and complete phenotyping information is available at www.humanislets.com.
Recruitment	Human pancreatic tissue samples were obtained from deceased organ donors through the Alberta Diabetes Institute IsletCore (www.isletcore.ca) with assistance from the Human Organ Procurement and Exchange (HOPE) program, Trillium Gift of Life Network (TGLN), and other Canadian organ procurement organizations. Donors were selected based on matching criteria (similar age, same sex, no diabetes or pancreatitis history) to minimize biological variability for method development.
Ethics oversight	Pancreas processing was approved by the Human Research Ethics Board at the University of Alberta (Pro00013094). All donors' families gave informed consent for the use of pancreatic tissue in research. The research was also approved by Etikprövningsmyndigheten (Dnr 2020-01690).

Note that full information on the approval of the study protocol must also be provided in the manuscript.

Field-specific reporting

Please select the one below that is the best fit for your research. If you are not sure, read the appropriate sections before making your selection.

[x] Life sciences [ ] Behavioural & social sciences [ ] Ecological, evolutionary & environmental sciences

For a reference copy of the document with all sections, see nature.com/documents/nr-reporting-summary-flat.pdf

Life sciences study design

All studies must disclose on these points even when the disclosure is negative.

Sample size	n=2 donors with two consecutive tissue sections each (4 sections total), yielding 864,485 segmented cells. No formal sample size calculation was performed as this is a methods development study. Sample size was chosen to demonstrate reproducibility across biological replicates while providing sufficient cellular diversity to identify rare endocrine populations. The large number of cells analyzed (>860,000) provides statistical power for identifying rare cell states.
Data exclusions	For PCA analysis, samples with fewer than 4,000 identified proteins were excluded to ensure robust dimensionality reduction. Missing values were imputed using a normal distribution, downshifted by 1.9 standard deviations with a width of 0.5 relative to the original distribution. For image analysis, cells were filtered based on size, excluding the bottom 1% and top 5% of the size distribution to remove segmentation artifacts. These criteria were pre-established based on quality control metrics.
Replication	Two biological replicates (two donors) with two technical replicates (consecutive tissue sections) each were analyzed. Common endocrine subtypes (e.g., $CHGA^{+}GCG^{+}$, $CPEP^{+}RBP4^{+}SST^{+}$) were consistently detected across all samples. Rare subtypes showed donor-specific variation, which is reported in Figure 2f. All mass spectrometry samples were processed with workflow replicates (n=2-3) for the contour dilution series optimization (Extended Data Fig. 4e-g).

Randomization	Randomization was not applicable to this study design. This is a methods development study analyzing all cells within defined tissue regions rather than comparing experimental treatment groups.
Blinding	Cell selection was automated, initially guided by k-means clustering followed by semi-supervised cluster refinement, without manual intervention. Data analysis and final cell annotations were not blinded to ensure rigorous quality control in handling biological samples and interpreting positive controls.

Reporting for specific materials, systems and methods

We require information from authors about some types of materials, experimental systems and methods used in many studies. Here, indicate whether each material, system or method listed is relevant to your study. If you are not sure if a list item applies to your research, read the appropriate section before selecting a response.

Materials & experimental systems

n/a	Involved in the study
☐	☒ Antibodies
☒	☐ Eukaryotic cell lines
☒	☐ Palaeontology and archaeology
☒	☐ Animals and other organisms
☒	☐ Clinical data
☒	☐ Dual use research of concern
☒	☐ Plants

Methods

n/a	Involved in the study
☒	☐ ChIP-seq
☒	☐ Flow cytometry
☒	☐ MRI-based neuroimaging

Antibodies

Antibodies used	A 15-plex DNA-barcoded antibody panel was used for CODEX multiplexed imaging targeting: CHGA, GCG, NPDC1, RBP4, CPEP, SST (endocrine markers); CBS, AMY2B (exocrine); HLA-DR (immune); PanCK, KRT19 (ductal); VIM, CD31, αSMA (vascular); CDH1 (membrane marker for segmentation); and Hoechst (nuclear stain). Pre-conjugated antibodies were purchased from Akoya Biosciences. Custom antibodies were sourced from commercial vendors as detailed in Supplementary Table 3, selected based on carrier-free formulation, concentration (>0.5 mg/mL), and prior validation for IHC in FFPE tissue.
Validation	All antibodies were validated by indirect immunofluorescence staining in FFPE human non-diabetic adult pancreatic tissue prior to use. Signal-to-noise ratio (SNR) cutoff of 3 was used for both primary antibody and antibody conjugate validations. Staining patterns were compared to reference images from manufacturers and the Tissue Atlas section of the Human Protein Atlas (HPA) to validate specificity. The antibody panel has been published as an Organ Mapping Antibody Panel (OMAP-6) as part of the Human BioMolecular Atlas Program (HuBMAP). Full validation details, including supplier names, catalog numbers, clone names, and lot numbers, are provided in Supplementary Table 3.

Plants

Seed stocks	*Report on the source of all seed stocks or other plant material used. If applicable, state the seed stock centre and catalogue number. If plant specimens were collected from the field, describe the collection location, date and sampling procedures.*
Novel plant genotypes	*Describe the methods by which all novel plant genotypes were produced. This includes those generated by transgenic approaches, gene editing, chemical/radiation-based mutagenesis and hybridization. For transgenic lines, describe the transformation method, the number of independent lines analyzed and the generation upon which experiments were performed. For gene-edited lines, describe the editor used, the endogenous sequence targeted for editing, the targeting guide RNA sequence (if applicable) and how the editor was applied.*
Authentication	*Describe any authentication procedures for each seed stock used or novel genotype generated. Describe any experiments used to assess the effect of a mutation and, where applicable, how potential secondary effects (e.g. second site T-DNA insertions, mosiacism, off-target gene editing) were examined.*
